# Global patterns of change in the burden of malnutrition in older adults from 1990 to 2021 and the forecast for the next 25 years

**DOI:** 10.3389/fnut.2025.1562536

**Published:** 2025-03-20

**Authors:** Le Li, Xiao Liu, Yujie Fang, Kailin Guo, Lu Li, Shuhan Cai, Chang Hu, Bo Hu

**Affiliations:** ^1^Department of Critical Care Medicine, Zhongnan Hospital of Wuhan University, Wuhan, China; ^2^Clinical Research Center of Hubei Critical Care Medicine, Wuhan, China

**Keywords:** aging, burden of disease, global burden, GBD study, malnutrition

## Abstract

**Background:**

Malnutrition poses a significant public health challenge, particularly as the global population ages. However, there is a notable lack of comprehensive literature analyzing the global burden of malnutrition among the elderly.

**Methods:**

Data on nutritional deficiencies indicators—prevalence, incidence, and disability-adjusted life years (DALYs)—for individuals aged 70 and older were extracted from the Global Burden of Diseases, Injuries, and Risk Factors Study (GBD) database from 1990 to 2021. The estimated annual percentage change (EAPC) was used to assess trends in malnutrition burden. Predictions for new cases over the next 25 years were also made.

**Results:**

In 2021, over 97.60 million cases of malnutrition among the elderly were reported globally, a 1.2-fold increase from 44.36 million cases in 1990. The global prevalence rate of malnutrition has decreased with an EAPC of −0.32%. Women experienced more cases than men but had a lower prevalence rate. Furthermore, in high socio-demographic index (SDI) regions, both prevalence rate and DALYs rates increased from 1990 to 2021, with EAPCs of 0.33% and 1.34%, respectively. The burden of malnutrition in the elderly was negatively correlated with SDI. Predictions from 2022 to 2046 estimated approximately 29.64 million new cases of malnutrition by 2046, despite a declining incidence rate.

**Conclusion:**

Despite a decline in malnutrition prevalence rate and DALYs rate since 1990, the burden remains high, particularly in high SDI regions where rates have increased. With a projected rise in new cases, effective prevention and management strategies are urgently needed to support the health of older adults.

## 1 Introduction

The global population is aging at an unprecedented rate, with the number of people aged 60 years and older expected to double to 2.1 billion by 2050 (1). This demographic shift presents significant medical and social challenges, particularly in maintaining health and quality of life in later years. The World Health Organization (WHO) has prioritized “healthy aging” in its agenda from 2016 to 2030, advocating for multifaceted actions that enable older adults to maintain health and engage actively in society (2). Nutrition plays a pivotal role in healthy aging, yet malnutrition remains a persistent global health challenge. The United Nations Decade of Action on Nutrition (2016–2025) has identified malnutrition as a primary concern (3). It encompasses various forms, including undernutrition (such as wasting, stunting, and underweight), micronutrient-related malnutrition, as well as overweight, obesity and diet-related noncommunicable diseases (4).

Among older adults, malnutrition persists as a prevalent issue, affecting approximately one-quarter of this demographic or putting them at risk (5). This vulnerability stems from age-related physiological changes, including reduced metabolic function, alterations in body composition, and declining organ function (6). Recognized as a common geriatric syndrome, malnutrition can lead to a range of health issues, including sarcopenia, cognitive decline, depression, increased infection risk, and worsening of chronic conditions (7–10). These factors collectively heighten the risk of adverse clinical outcomes, including increased mortality, longer hospital stays, and higher healthcare costs (5). As the global population ages, the number of malnourished older adults is expected to rise (5). A comprehensive analysis of the global burden and distribution of malnutrition among the elderly can address existing research gaps, reveal regional disparities, and provide a scientific basis for formulating targeted global public health policies.

Grounded in the intersection of population aging and the persistent burden of malnutrition, we used data from the Global Burden of Disease (GBD) database—the most extensive source of global health data. Numerous previous studies have utilized GBD data to assess epidemiological trends in malnutrition. Yu et al. found that the global incidence of malnutrition in 2019 was 2207.71 cases per 100,000, with the highest risk of nutritional deficiency among children under five (11). Studies by MAO et al. and Liu et al. further confirmed that from 1990 to 2019, the situation regarding child malnutrition remained severe, with no significant improvement in health inequalities related to socioeconomic development (12, 13). Additionally, maternal malnutrition continued to pose a substantial health burden globally and across regions (14, 15). Liu et al. also observed that the incidence of malnutrition among individuals over 15 in low- and middle-income countries was still on the rise (16). While existing research primarily focused on malnutrition in vulnerable groups like children and women, particularly in low- and middle-income countries (16), there is currently a lack of comprehensive descriptions of nutritional deficiencies in older adults, and the global burden and distribution of malnutrition in this population remain unclear.

This study aimed to describe the trends in nutritional deficiencies among individuals aged 70 and above globally, regionally, and nationally from 1990 to 2021, alongside forecasting future trends over the next 25 years. It assessed the contributions of aging, population dynamics, and epidemiological changes to the prevalence of nutritional deficiencies across the overall population. By focusing on individuals aged 70 and above, this study supported the WHO’s healthy aging agenda and laid a foundation for long-term health planning and policy development.

## 2 Method

### 2.1 Study data

The GBD 2021 is an extensive international collaboration that systematically evaluates global health status and disease burden through comprehensive data collection and analysis. It integrated data from diverse sources, including population censuses, death registries, hospital records, epidemiological studies, and international organizations. These data underwent standardization (cleaning, adjustment, and imputation) and uniform classification like International Classification of Diseases (ICD) coding, with disease burden estimates generated using statistical models like DisMod-MR. Rigorous verification and expert review ensured data accuracy, and results were published and updated annually via the GBD Compare tool and official website. The GBD study offers epidemiological data from 204 nations and territories, and encompasses a wide range of diseases, injuries and risk factors (17–19). The insights gained from these data serve as a foundation for epidemiological research, aiding countries in formulating public health policies and effectively allocating medical resources.

We utilized the GBD Results tool^1^ to extract key epidemiological indicators for the elderly, including number and rate of incidence, prevalence, and disability-adjusted life years (DALYs). DALYs quantify the burden of disease by reflecting the healthy life years lost due to both premature death (years of life lost, YLLs) and disability (years lived with disability, YLDs). The definition of malnutrition used in this study aligned with the concept of nutritional deficiencies in the GBD. Nutritional deficiencies, categorized based on the ICD codes as outlined in Supplementary Table S1, encompassed the forms of malnutrition defined by the World Health Organization.

The Socio-demographic Index (SDI) is a comprehensive indicator reflecting the social and economic factors impacting regional health statuses (20). Calculated via data on income, education level, and fertility rate, the SDI categorizes 204 countries and regions into five groups: low, low-middle, middle, high-middle, and high. For further details on data sources and the extraction methodology used in this study were displayed in eMethods in Supplementary material.

### 2.2 Statistical analysis

In this study, we analyzed data on nutritional deficiencies in individuals aged 70 and older from GBD 2021, assessing the impact of aging on the overall prevalence of nutritional deficiencies across all age groups from 1990 to 2021. Additionally, we predicted the number of new cases globally over the next 25 years.

We reported cases and rates of prevalence, and DALYs rates. The 95% uncertainty interval (UI) were calculated using the 2.5th and 97.5th percentiles from 1,000 estimates derived from the GBD algorithm. Data cleaning (“dplyr” package), calculations (“tidyverse” package), and visualizations (“ggplot2” package) were performed using R software (version 4.3.1).

Trends in the prevalence of nutritional deficiencies were assessed using the Average Percentage Change (APC) and the Estimated Annual Percentage Change (EAPC). The Joinpoint regression model was used to assess trends in malnutrition prevalence among older adults because it identified significant changes in trends over time. By detecting trend shifts (joinpoints), it segmented data into distinct phases and calculated the annual percentage change (APC) for each interval (21). The EAPC was derived from a regression model that fitted the natural logarithm of the calendar year to determine the long-term trend in prevalence. Detailed equations and models for the joinpoint regression analysis, along with the calculations for the EAPC method, are available in the eMethods of Supplementary material.

We extracted data on the number of nutritional deficiencies across all age groups from 1990 to 2021. The raw case numbers were classified by aging, demographic factors, and epidemiological changes (including age and population standardized prevalence) to assess the impact of population growth, aging, and epidemiological changes on nutritional deficiencies over the past 32 years.

To forecast the global number and incidence of new cases among elderly in different sexes over the next 25 years, we utilized a log-linear age-period-cohort model based on recent trends. For the last three to four 5-year observation periods, we applied a power function to extrapolate growth, predicting that the recent linear trend will decline by 25, 50, and 75% in the second, third, and fourth projection periods, respectively. The 25, 50, and 75% decline targets were chosen to simulate the potential impact of varying intervention intensities on disease burden. The projected count of new cases for 2046 was estimated by calculating a weighted average of the projected incidence rates from the last two projection periods and applying that ratio to United Nations population projections for each year. Population data, both actual and projected, were obtained from the Institute for Health Metrics and Evaluation (22). Functions for this analysis were available in R Studio and the NORDPRED Package (23).

## 3 Results

### 3.1 Global level

**Figure 1:**
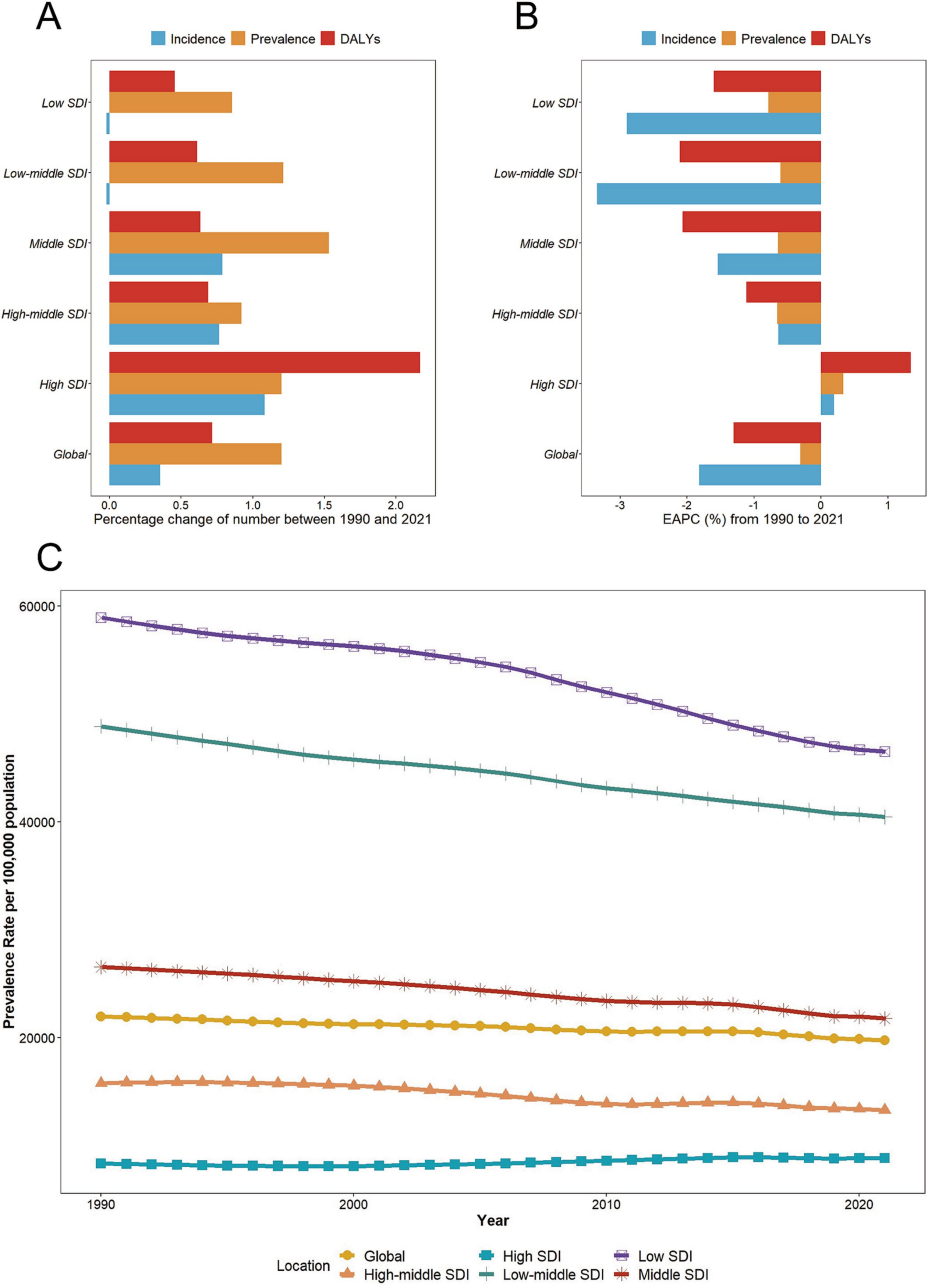
Temporal trend of malnutrition burden among individuals over 70 in global and 5 territories. **(A)** Percentage change in cases of prevalent, incident, and DALYs in 1990 and 2021. **(B)** The EAPC of prevalence, incidence, and DALYs rates from 1990 to 2021. **(C)** The rates of prevalence, incidence, and DALYs from 1990 to 2021. SDI, Socio-demographic Index; EAPC, estimated Annual percentage change; DALYs, disability-adjusted life years.

The participant flowchart is depicted in Supplementary Figure S1. Global malnutrition cases among individuals over 70 increased from 44.36 million (95% UI: 42.99–45.71 million) in 1990 to 97.60 million (95% UI: 94.05–100.99 million) in 2021, reflecting a 1.2-fold rise (Table 1). Additionally, the prevalence rate of nutritional deficiencies per 100,000 elderly decreased from 21,961 in 1990 to 19,742 in 2021, with an EAPC of −0.31 (95% CI: −0.32 to −0.29), indicating a downward trend over 32 years (Figure 1). Significant prevalence rate changes were noted in 1998, 2005, 2010, 2015, and 2019 via joinpoint regression analysis, notably a marked decline from 2015 to 2019 (APC = −0.81%, *p* < 0.05) (Supplementary Figure S2A). Despite increases in incidence and DALYs cases since 1990, the overall incidence and DALYs rate have trended downward (EAPCs < 0) (Figure 1). Both men and women experienced increased prevalence and DALYs cases, yet their rates declined. Women consistently showed higher cases of prevalence and DALYs, while men had higher prevalence rates. Since 2006, men’s DALY rate has fallen below that of women (Supplementary Figure S3).

**Table 1:** The case number and prevalence of malnutrition among individuals over 70 in 1990 and 2021, and its temporal trends from 1990 to 2021.

Location	1990		2021		1990–2021
	Case number (95% UI)	Rate (95% UI)	Case number (95% UI)	Rate (95% UI)	EAPC% (95% CI)
Global	44362384.1 (42989676.3–45713036.8)	21960.8 (21281.3–22629.4)	97596260.3 (94049605.2–100992122.8)	19741.6 (19024.2–20428.5)	−0.31 (−0.32 to 0.29)
High SDI	5766209.7 (5307568.2–6213531.1)	8349.9 (7685.8–8997.7)	12687705.3 (11612723.2–13985171.8)	8842.8 (8093.6–9747.1)	0.33 (0.27–0.40)
High-middle SDI	8113798.9 (7698446–8526403.1)	15766.5 (14959.4–16568.3)	15594864.1 (14801536.8–16442649.7)	13283.3 (12607.6–14005.5)	−0.65 (−0.71 to 0.60)
Middle SDI	12124863.5 (11655910.9–12600946.9)	26534.3 (25508–27576.2)	30682261.1 (29306523.8–31960537.8)	21761.4 (20785.7–22,668)	−0.64 (−0.66 to 0.62)
Low-middle SDI	12811981.7 (12355615–13298662.6)	48855.4 (47115.1–50711.2)	28357425.8 (27324164.8–29309149.2)	40455.6 (38981.5–41813.3)	−0.60 (−0.61 to 0.59)
Low SDI	5499941.6 (5360776.1–5654098.1)	58935.6 (57444.4–60587.5)	10,200,675 (9856974.8–10501035.4)	46506.5 (44939.5–47875.9)	−0.79 (−0.85 to 0.73)
Andean Latin America	172369.2 (148203.5–200,838)	16891.5 (14523.3–19681.3)	374981.5 (325693.8–438165.3)	11431.2 (9928.7–13357.4)	−1.36 (−1.42 to 1.30)
Australasia	102868.1 (84502–123,336)	7058.5 (5798.3–8462.9)	271708.8 (227974.7–322496.6)	7457.6 (6257.2–8851.6)	0.28 (0.22–0.34)
Caribbean	241,428 (217234.8–275051.3)	16355.2 (14716.3–18,633)	466501.6 (418341.7–532582.5)	14580.5 (13075.3–16645.9)	−0.46 (−0.52 to 0.41)
Central Asia	423575.1 (392939.8–457611.6)	18798.2 (17438.6–20308.8)	527173.9 (478135.1–584608.6)	15509.1 (14066.4–17198.7)	−0.83 (−0.93 to 0.72)
Central Europe	1,550,384 (1446940.2–1664806.7)	19627.3 (18317.7–21075.8)	2166315.2 (2032769.3–2305533.9)	14593.4 (13693.8–15531.3)	−1.05 (−1.13 to 0.98)
Central Latin America	663078.6 (621791.2–703408.3)	16413.9 (15391.8–17412.2)	1642540.8 (1558205.1–1,734,853)	12023.3 (11406–12699.1)	−0.89 (−0.94 to 0.85)
Central Sub-Saharan Africa	394713.6 (359903.6–426,682)	48693.9 (44399.6–52637.7)	698921.9 (633290.2–763565.3)	37070.7 (33589.7–40499.4)	−0.78 (−1.05 to 0.50)
East Asia	7963820.5 (7601809.9–8,388,743)	20465.3 (19535–21557.3)	17571374.1 (16611682.4–18638817.3)	14218.7 (13442.1–15082.4)	−1.29 (−1.36 to 1.23)
Eastern Europe	1698669.8 (1462188.9–1972603.9)	11212.4 (9651.5–13020.6)	2239528.1 (1934679.6–2,637,175)	10484.1 (9057–12345.7)	−0.36 (−0.46 to 0.27)
Eastern Sub-Saharan Africa	1,875,190 (1808864.2–1941121.7)	60208.9 (58079.3–62325.8)	2666663.4 (2545000.1–2790301.1)	38702.3 (36936.5–40496.7)	−1.50 (−1.62 to 1.38)
High-income Asia Pacific	1,106,613 (888330.2–1357625.5)	9831.5 (7892.2–12061.6)	3770425.6 (3138228–4637368.3)	10791.4 (8982–13272.7)	0.39 (0.20–0.57)
High-income North America	1482961.6 (1280225.2–1661359.2)	6,386 (5513–7154.2)	3058103.5 (2596079.2–3553974.7)	7061.2 (5994.4–8206.2)	0.56 (0.45–0.66)
North Africa and Middle East	2091304.4 (1990045.3–2211906.7)	28958.8 (27556.7–30628.8)	3948913.9 (3694144.4–4186993.9)	19420.2 (18167.3–20,591)	−1.22 (−1.24 to 1.19)
Oceania	33429.7 (30715.9–36201.2)	32156.4 (29545.9–34822.4)	81711.2 (74453.4–90649.4)	29620.8 (26989.8–32860.9)	−0.08 (−0.14 to 0.02)
South Asia	14109794.2 (13552218.3–14713420.2)	60073.1 (57699.2–62643.1)	38317499.9 (37065714.1–39525418.9)	52335.9 (50626.2–53985.8)	−0.45 (−0.47 to 0.44)
Southeast Asia	3714762.2 (3488889.7–3934289.1)	34027.7 (31958.6–36038.5)	8237010.9 (7496325.4–9,131,518)	27385.7 (24923.2–30359.7)	−0.74 (−0.84 to 0.64)
Southern Latin America	419621.8 (366988.6–477350.2)	15924.1 (13926.7–18114.8)	763839.1 (664728.4–857919.9)	13894.5 (12091.7–15605.9)	−0.42 (−0.56 to 0.28)
Southern Sub-Saharan Africa	233986.6 (205789.4–266234.2)	18024.5 (15852.4–20508.6)	369015.9 (329736.1–408008.2)	13836.9 (12364–15,299)	−0.77 (−0.81 to 0.74)
Tropical Latin America	1329879.4 (1125611.1–1552069.7)	30,458 (25779.6–35546.7)	2595695.4 (2184917.8–3137676.8)	18071.7 (15211.8–21,845)	−1.62 (−1.64 to 1.60)
Western Europe	3065963.9 (2815115.7–3336260.1)	8209.7 (7538–8933.4)	5371748.5 (4925525.3–5841267.9)	8164.2 (7486–8877.8)	0.17 (0.09–0.25)
Western Sub-Saharan Africa	1687970.3 (1591173.9–1781917.9)	42000.9 (39592.4–44338.6)	2,456,587 (2310372.1–2622777.8)	30143.2 (28349.1–32182.5)	−1.03 (−1.06 to 1.01)

EAPC, estimated annual percentage change.

### 3.2 SDI regional level

In 2021, the absolute number of prevalence cases and DALYs from nutritional deficiencies among the elderly peaked in the middle SDI, reaching 30.68 million (95% UI: 29.31–31.96 million) and 1.02 million (95% UI: 0.84–1.26 million), respectively. The Low-middle SDI accounted for the highest number of incidence cases at 5.24 million (95% UI: 4.51–6.12 million). Notably, the low SDI exhibited the highest prevalence, incidence, and DALY rates (Table 1; Supplementary Tables S2, S3; Figure 1C; Supplementary Figure S4). Compared to 1990, the high SDI experienced significant increases in incidence cases and DALYs, with rises of approximately 108 and 217%, respectively, while incidence cases decreased in low SDI and low -middle SDI (Figure 1A). Surprisingly, from 1990 to 2021, the high SDI saw rapid increases in incidence, prevalence, and DALYs rates, with EAPCs of 0.20 (95% CI: 0.03–0.37), 0.33 (95% CI: 0.27–0.40), and 1.34 (95% CI: 1.14–1.54), respectively (Table 1; Supplementary Tables S2, S3; Figure 1B). In contrast, other regions showed a downward trend, with all EAPCs below 0. Joinpoint regression analysis revealed that over the past 32 years, prevalence rate in middle SDI, low-middle SDI, and low SDI consistently declined. However, the high SDI experienced a prolonged increase following an initial decline, a brief decrease, and then another rise, with the most significant growth observed between 2006 and 2015 (APC = 0.74, *p* < 0.05) (Supplementary Figure S2).

### 3.3 GBD regional level

**Figure 2:**
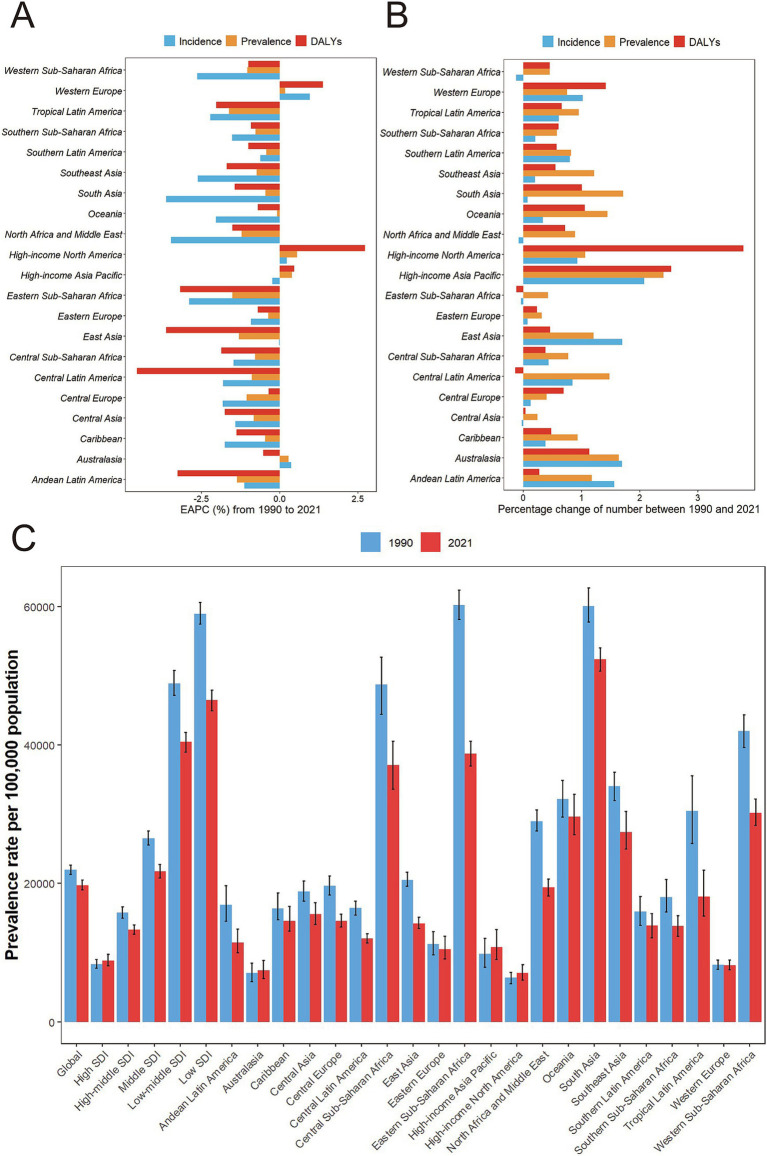
Temporal trend of malnutrition burden among individuals over 70 in GBD regions. **(A)** Percentage change in cases of prevalent, incident, and DALYs in 1990 and 2021. **(B)** The EAPC of prevalence, incidence, and DALYs rates from 1990 to 2021. **(C)** Prevalence of malnutrition among individuals over 70 by regions and year with 95% uncertainty intervals. DALYs, disability-adjusted life years; GBD, the Global Burden of Disease.

In most regions, the prevalence, incidence, and DALYs rate of nutritional deficiencies in older adults have decreased over time. However, certain areas, including Australasia, High-income Asia Pacific, and High-income North America-being more developed regions-have shown an upward trend (Figure 2; Table 1; Supplementary Tables S2, S3). Compared to 1990, the number of prevalence cases in all GBD regions significantly increased by 2021. Notably, high-income North America witnessed the largest increase in DALYs at 378.3% (Figure 2B). Over the past 32 years, most regions have experienced declines in incidence, prevalence, and DALY rates, with the largest reductions in Tropical Latin America and Eastern Sub-Saharan Africa, reflecting EAPCs of −1.62 (95% CI: −1.64 to −1.60) and −1.50 (95% CI: −1.62 to −1.38), respectively. Additionally, Central Latin America and East Asia have seen the most significant declines in DALYs rates, with EAPCs of −4.56 (95% CI: −4.67 to −4.46) and −3.63 (95% CI: −4.36 to −2.89) (Figure 2A). Conversely, prevalence and DALYs rates increased in High-income North America, High-income Asia Pacific, and Western Europe, with EAPCs of 0.56 (95% CI: 0.45–0.66), 0.39 (95% CI: 0.20–0.57), and 0.17 (95% CI: 0.09–0.25) for prevalence rate, and 2.73 (95% CI: 2.41–3.05), 0.46 (95% CI: 0.28–0.64), and 1.39 (95% CI: 1.22–1.55) for DALY rates, respectively (Table 1; Supplementary Tables S2, S3). Overall, while the prevalence of nutritional deficiencies among older adults decreased in most regions, increases were solely observed in Australasia, High-income North America, and Western Europe (Table 1; Figure 2).

### 3.4 Countries level

**Figure 3:**
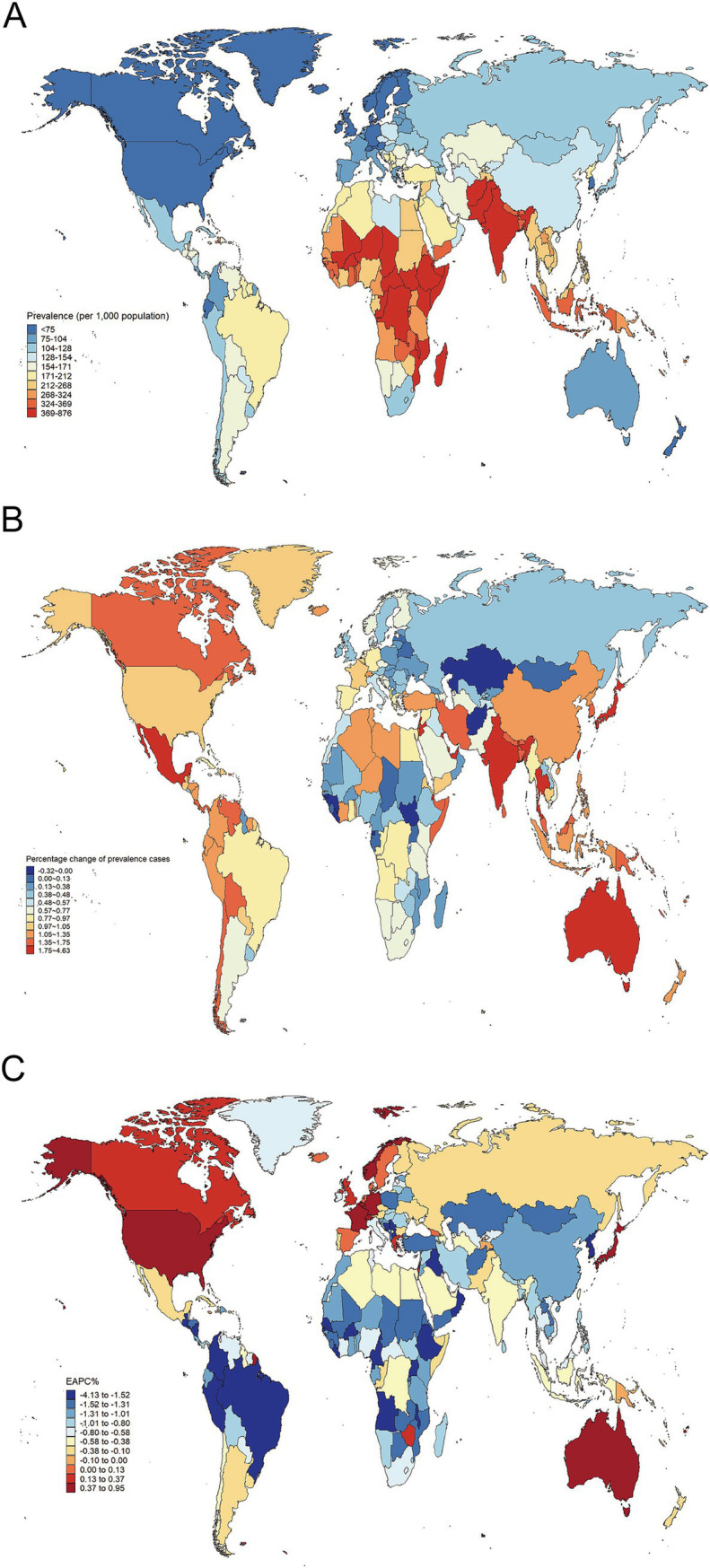
**(A)** The prevalence of malnutrition among individuals over 70 in 2021. **(B)** The relative change in case number of prevalence of malnutrition among individuals over 70 from 1990 to 2021. **(C)** The EAPC of prevalence of malnutrition among individuals over 70 from 1990 to 2021. EAPC, estimated annual percentage change.

In 2021, India had the largest number of incidence cases, prevalence cases, and DALYs in nutritional deficiencies among the elderly, followed by China (Supplementary Tables S4–S6). Countries such as Australia, Jordan, Qatar, Kuwait, Singapore, Northern Mariana Islands, Andorra, Mexico, and the United States Virgin Islands have seen significant increases in both the absolute number of incidence cases and prevalence cases, with percentage changes in incidence cases ranging from 160 to 509% and prevalence cases changes from 175 to 463%. Additionally, Norway, Canada, Qatar, and the United States experienced the largest changes in absolute DALYs, with percentage changes from 172 to 474% (Figure 3; Supplementary Figures S5, S6). All these countries with high percentage changes in epidemiological and burden metrics belong to the high SDI or high-middle SDI nations. Furthermore, in 2021, Somalia (high SDI) had the highest rates of incidence, prevalence, and DALYs rate (92655.9, 87626.4, 3683.6 per 100,000 population respectively) (Supplementary Tables S4–S6).

Over the past 32 years, most countries have shown a downward trend in the incidence, prevalence, and DALYs rates of nutritional deficiencies in the elderly, with Equatorial Guinea experiencing the most significant decreases in both prevalence rate and incidence rate, as reflected in EAPCs of −6.9 (95% CI: −7.22 to −6.57) and −4.13 (95% CI: −4.53 to −3.73), respectively. The Democratic People’s Republic of Korea recorded the largest decline in DALY rates with an EAPC of −8.77 (95% CI −13.09 to −4.23). However, a few countries, including Switzerland, San Marino, Norway, Belgium, Germany, Andorra, Monaco, France, Canada, the United States, Japan, Iceland, and Luxembourg, have observed increases in incidence, prevalence, and DALY rates. The largest increase in prevalence rate was observed in Switzerland (EAPC: 0.95, 95%CI: 0.86–1.04), while Germany experienced the highest rise in incidence rates (EAPC: 4.13, 95%CI: 3.78–4.52). Norway had the highest increase in DALY rates (EAPC: 6.51, 95%CI: 5.06–7.98) (Supplementary Tables S4–S6; Figure 3; Supplementary Figures S5, S6). These findings indicated that countries experiencing a rising burden are more likely to be high SDI nations.

### 3.5 Association with the socio-demographic index

At the regional level, the prevalence and DALYs rates of nutritional deficiencies among older adults decreased with increasing SDI from 1990 to 2021. The burden of nutritional deficiencies in the elderly (DALYs rate) showed a significant negative correlation with SDI (*ρ* = −0.87, *p* < 0.001), indicating that regions with higher socioeconomic development tended to have lower burdens of nutritional deficiencies. Specifically, the burden was higher than expected in South Asia, Southeast Asia, Central Latin America, and Andean Latin America, while it was lower than expected in Western Sub-Saharan Africa, Oceania, North Africa and the Middle East, Central Asia, and Australasia (Supplementary Figure S7A).

At the national level, improvements in social and economic development in 2021 generally led to a decline in the burden of nutritional deficiencies among older adults. Countries such as Somalia, Kiribati, Mali, Sierra Leone, South Sudan, Indonesia, Madagascar, Eritrea, Zimbabwe, and India experienced burdens much higher than expected, whereas Niger, Afghanistan, Chad, Burkina Faso, and Senegal had burdens significantly lower than anticipated (Supplementary Figure S8).

### 3.6 The influential factors for EAPC of incidence

A notable correlation existed between the EAPC of incidence rate and the incidence rate in 1990, as well as the Human Development Index (HDI) in 2022. Incidence rate of nutritional deficiencies among older adults in 1990 reflected baseline disease incidence rate, while the HDI served as a proxy for health care levels and accessibility across countries. There was a U-shaped relationship between EAPC and incidence rate (*ρ* = −0.70, *p* < 0.001), showing a negative correlation when the incidence rate was below 33,360 per 100,000, and a positive correlation above this threshold (Supplementary Figure S7B). Moreover, a significant positive correlation was found between EAPC of incidence rate and HDI (ρ = 0.65, *p* < 0.001). From 1990 to 2021, the incidence rate of nutritional deficiencies among older adults increased more rapidly in countries with higher HDI (Supplementary Figure S7C).

### 3.7 Drivers of nutritional deficiencies epidemiology

**Figure 4:**
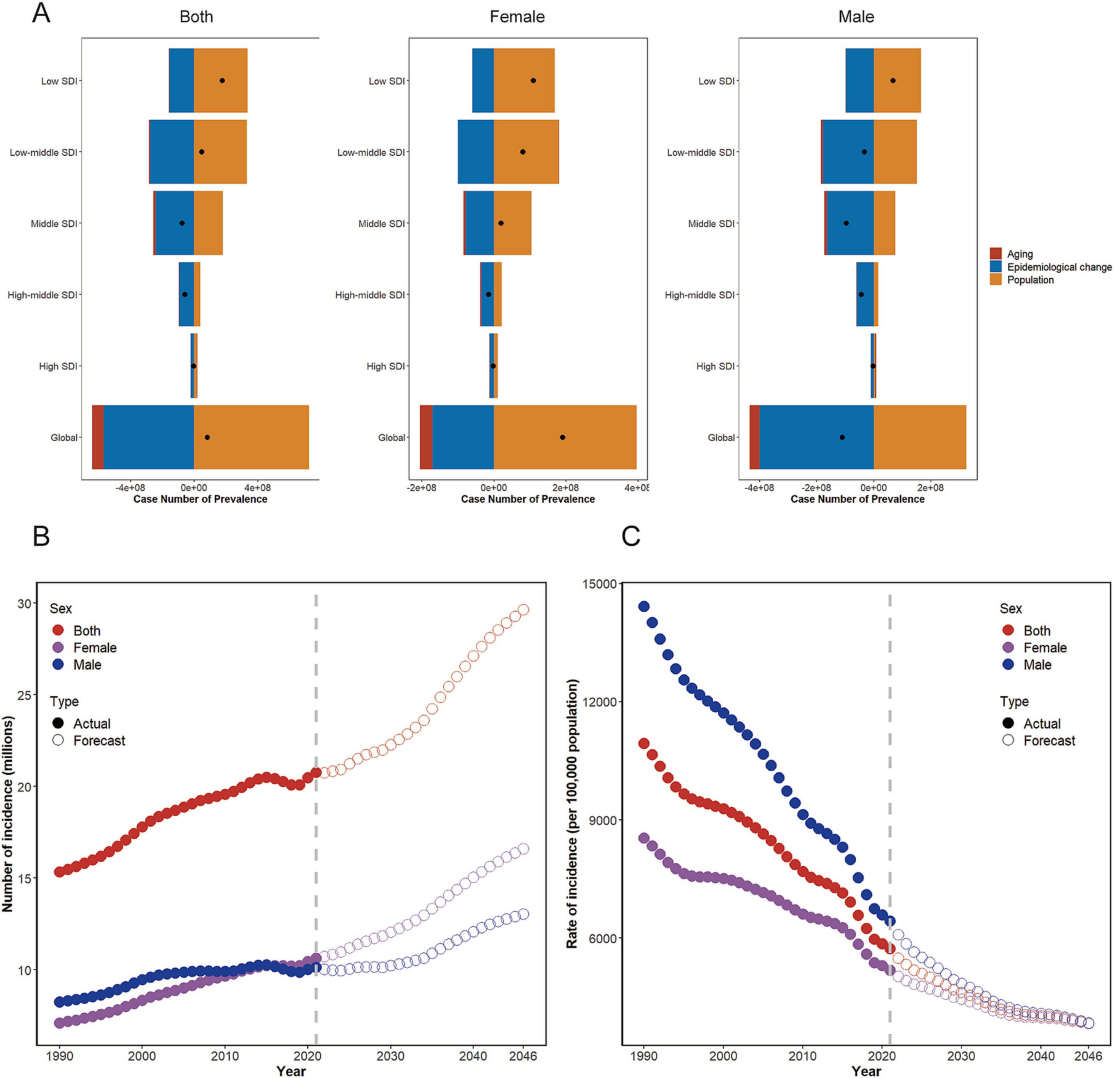
**(A)** Changes in prevalence cases of malnutrition in overall population at all ages at according to population-level determinants including aging, population growth and epidemiological change from 1990 to 2019 at the global level and by SDI quintiles stratified by sexes. **(B,C)** Changes in global **(B)** incidence cases and **(C)** incidence over the next 25 years, based on Nordpred. SDI, sociodemographic index.

To explore the impacts of aging, population changes, and epidemiological changes on nutritional deficiencies, we conducted a decomposition analysis of case numbers by age group, incorporating variables such as population, aging, age and standardized incidence and mortality (collectively termed epidemiological changes). Overall, the number of cases has significantly increased globally, particularly in regions with low and low-middle SDI. From 1990 to 2021, population and aging contributed −87.54 and 898.61% respectively, respectively, to the rise in the prevalence cases of nutritional deficiencies, with population being the primary driver of the overall increase. Aging had the most significant impact on prevalence cases in middle SDI (17.75%) and high SDI (−18.06%). The effects of aging on prevalence cases exhibited notably gender discrepancies globally, especially in regions with high SDI (men: −67.36%; women: 42.73%). Increases in overall and male in high SDI, as well as female in regions with low and low-middle SDI, could be attributed to aging. Changes in nutritional deficiencies epidemiology were primarily driven by population growth in low-middle and low SDI. Over the past 32 years, age-adjusted and population-adjusted epidemiological changes in nutritional deficiencies have increased globally (Supplementary Table S7; Figure 4A).

### 3.8 Projected changes in nutritional deficiencies in older adults based on Nordpred

According to the Nordpred model, the absolute incidence number of nutritional deficiencies among individuals aged over 70 was expected to continue increasing over the next 25 years, while the incidence rate was projected to gradually decrease. From 2022 to 2046, it is expected that there will consistently be more new cases in women than in men worldwide, with the gender gap in incidence gradually narrowing. By 2046, the number of new cases of nutritional deficiencies among the elderly was estimated to reach 29,638,914, with women surpassing men (16,603,423 vs. 13,035,490). Despite the anticipated reduction in the incidence rate, predicted to be 3841.66/100,000, the incidence rate in men remains higher than in women (3850.44 vs. 3834.80, per 100,000 population) (Figures 4B,C, Supplementary Table S8).

## 4 Discussion

The United Nations Nutrition Strategy 2022–2030 emphasizes the importance of improving the nutritional status among the elderly to enhance quality of life, reduce healthcare costs, and foster social well-being (24). Malnutrition is a prevalent and concerning health issue among the elderly, characterized by higher morbidity, and mortality of its complications, as well as medical expenses, thereby resulting in a significant overall disease burden (10). A thorough understanding of the prevalence of malnutrition in this demographic is essential for assessing health progress and promoting healthy aging. However, there is currently a notable shortage of systematic literature regarding the comprehensive analysis of the epidemiology and burden of malnutrition among the elderly. Using data from the Global Burden of Disease Study 2021, this study provided the first comprehensive analysis of malnutrition in individuals aged 70 and older over the past 32 years. Findings indicated that while the prevalence of malnutrition and DALYs rate among the elderly decreased from 1990 to 2021, the total number of affected older adults and the overall burden remained critically high.

Gender differences were significant, with higher absolute numbers of cases and DALYs in women, while prevalence and DALYs rates were greater in men. This disparity was partly attributed to the greater number of women over 70 (Supplementary Table S9). Women’s higher life expectancy (73.8 years in 2021 compared to 68.4 years for men) also played a crucial role (25). Additionally, gender may influence dietary behaviors and responses to nutritional interventions, with women demonstrating better adherence to nutritional interventions (26–28).

The trends in prevalence and burden observed in this study challenged the conventional understanding of the relationship between SDI and malnutrition. Typically, higher SDI levels correlate with more robust health systems and improved health services, leading to a reduced disease burden. Contrarily, this study revealed that the only increasing trends in malnutrition incidence, prevalence, and DALYs among the elderly over the past 32 years were found in high SDI areas. Several factors may contribute to this unexpected outcome: (1) Longevity and complex health needs. The elderly face multifaceted health challenges, including rising rates of non-communicable diseases such as cardiovascular disease, diabetes, and cancer. These conditions necessitate intricate dietary management and can impair nutritional absorption, thereby elevating the risk of malnutrition. (2) Urbanization and lifestyle changes. Rapid urbanization and industrialization have led to significant lifestyle modifications, including sedentary behavior, increased stress, and poor dietary practices. These shifts are closely linked to rising obesity rates and micronutrient deficiencies (29). (3) Impact of the COVID-19 pandemic. The pandemic has intensified the nutrition crisis, with joinpoint regression analysis revealing substantial changes in malnutrition prevalence in high SDI areas since 2019 (APC = 0.24, *p* < 0.05). Numerous studies indicate that individuals infected with COVID-19 (30, 31), particularly the elderly (32), exhibit higher rates of malnutrition. (4) Impact of intra-national socioeconomic disparities. Socioeconomic inequalities within high-SDI countries are a key driver of malnutrition. For instance, in Australia, low-income families and marginalized communities faced a higher risk of malnutrition (33). (5) Policy implementation Challenges. There may be insufficient funding for nutrition programs or a lack of coordination between public health initiatives and local communities. 6. Social safety net deficiencies. According to the World Food Programme, food assistance programs for vulnerable groups, such as children, pregnant women, and the elderly, often face implementation gaps, leaving some at risk of malnutrition (34).

The differences in disease burden across geographic regions are closely tied to social and cultural factors. Africa and South Asia experienced the highest rates of malnutrition and DALYs rate. In Africa, malnutrition was driven by issues such as poverty, rapid population growth, inefficient agricultural practices, poor governance and corruption, the human immunodeficiency virus (HIV) epidemic, malaria, diseases such as the Ebola virus and the COVID-19 pandemic, and armed conflicts (35, 36). South Asia faced increased vulnerability to climate-related malnutrition (37). In contrast, Tropical Latin America has achieved the fastest decline in prevalence of malnutrition through coordinated multisectoral policies (38). Countries in this region have recognized the right to food and health in their constitutions (39), established food security councils, and developed guidelines to combat stunting, wasting, and nutrient deficiencies (40). Dietary improvement initiatives comprised a consumption tax on sugary drinks in Mexico and mandatory food labeling in Chile and Ecuador (41–43). Furthermore, several countries, including Chile and Colombia, have implemented school-based interventions designed to promote healthy eating (40). On the other hand, the burden of malnutrition among the elderly in high-income North America is on the rise. Economic factors and resultant food insecurity are contributing to the rising disease burden faced by this demographic. Following the Great Recession, economic pressures have significantly affected economically vulnerable seniors. In the United States, despite high health spending, the value of spending on health care for older adults has declined (44). Research indicated that three-quarters of malnourished patients experienced care gaps (45). Furthermore, while poverty rates among older adults have significantly declined since 1975, they have stagnated since 2011. Financial adversity stemming from previous economic downturns may adversely affect the financial security of older adults in retirement (46). Unlike the overall population, food insecurity rates among older adults had not returned to pre-Great Recession levels as of 2021 (46). Therefore, effective policies, legal protections, medical education, and economic support are essential for improving malnutrition. Measures should be actively taken to address elderly malnutrition and its related disease burden.

Population growth and aging are significant factors influencing the global, regional, and national prevalence of malnutrition. However, current epidemiological trends—such as improvements in environmental factors and early detection, diagnosis, and treatment of malnutrition—have not sufficiently mitigated the impact of these demographic changes. Regarding aging, with the exception of high SDI areas, aging was surprisingly a driving factor for the reduction in the number of malnutrition cases in the other four areas. This may be due to the study’s decomposition analysis, which encompassed all age groups, as children and pregnant women are also high-risk populations for malnutrition. In 2019, the number of malnourished children reached over 435 million (13), significantly exceeding the 44.36 million elderly affected in our study. Furthermore, the proportion of malnutrition among individuals aged 70 and older increased from 2.5% of total cases in 1990 to 5.3% in 2021 (Supplementary Table S10). Despite this increase, the elderly still represented a small fraction of malnutrition cases. Additionally, aging may have led to greater attention to elderly health, resulting in improved nutrition screening and intervention measures. Research indicated that older adults were more likely than younger individuals to use dietary supplements and to be aware of nutritional risks (28, 47).

Projections based on existing data sets indicated that while the incidence of malnutrition in individuals aged over 70 was expected to decline, the absolute number of new cases among this age group would remain significant due to population growth and continued aging. As life expectancy increases, older adults are likely to spend more time living with diseases and disabilities (48). Chronic wasting diseases, such as chronic obstructive pulmonary disease, heart failure, and chronic renal failure, often lead to malnutrition due to inflammation (5, 49). Proinflammatory cytokines (like TNFα, CRP, IL-1β, and IL-6) impacted the nervous system and reduce appetite by promoting skeletal muscle breakdown, inhibiting gastric emptying, and disrupting appetite hormone function (49–51). Additionally, conditions such as stroke, Parkinson’s disease, and dementia could impair eating and swallowing in the elderly, further contributing to malnutrition (5, 49). Even when nutrient intake appears adequate, factors such as decreased physical performance and drug-nutrient interactions may adversely affect nutrient metabolism (52). Thus, malnutrition, though not immediately fatal, can lead to serious clinical consequences if not addressed promptly. Researches have shown that malnutrition was closely linked to osteopenia, sarcopenia, frailty syndromes, immune dysfunction, delayed postoperative recovery, increased hospitalization and readmission rates, and elevated mortality (10). These factors significantly diminished the quality of life for older individuals and substantially raised healthcare costs. In the United States, the economic burden of morbidity, mortality, and direct medical expenses related to disease-related malnutrition was estimated at approximately $157 billion, with $51.3 billion attributed to those aged 65 and older (53). Therefore, given that the causes of malnutrition can be identified through early screening and prediction, timely interventions often lead to significant improvements in health outcomes and quality of life for older adults.

While the global decline in the prevalence and DALYs rates of malnutrition among the elderly has brought us an optimistic outlook, there are still significant disparities in the burden of malnutrition between geographical regions. Malnutrition in the elderly remains a prominent public health issue. Various factors contribute to malnutrition in older adults, highlighting the need for multifaceted collaboration to address this challenge. At the global level, several international organizations have initiated important frameworks, such as the United Nations Nutrition Strategy 2022–2030 and the Decade of Action on Nutrition. However, specific issues related to malnutrition in the elderly require further attention and tailored guidelines. National responses vary according to socioeconomic conditions. In countries with stronger economies and abundant medical resources, efforts should focus on expanding screening and implementing early targeted interventions for malnutrition among the elderly. Standardized screening tools (such as Nutrition Risk Screening 2002 or Mini Nutritional Assessment Short-Form) should be used for regular assessments, combined with multimodal intervention strategies, including personalized dietary guidance, oral nutritional supplements, and exercise interventions, to improve the nutritional status of older adults. Additionally, these nations should allocate more resources to explore strategies for mitigating the impact of population aging on malnutrition burdens. Conversely, in countries with lower socioeconomic status, priorities should include economic development and addressing poverty and food insecurity. Successful experiences from Tropical Latin America can be adopted, integrating resources and policies across sectors such as health, agriculture, education, and social security through cross-sector collaboration. For example, combining community health programs, nutrition education initiatives, agricultural development, and food assistance programs can help improve malnutrition among older adults. Furthermore, international partnerships can provide technical and financial support, such as funding from mechanisms like the Global Nutrition Fund, while international organizations and non-governmental organizations can assist in establishing effective nutrition monitoring and evaluation systems. At the individual level, cultivating healthy lifestyle habits is crucial. Individuals could adhere to national recommendations, such as the World Health Organization’s healthy eating guidelines, to promote better nutrition. Moreover, fostering good habits in children will ultimately help reduce future disease burdens and healthcare costs, as these children grow into informed and healthier older adults, better equipped to face future nutritional challenges.

Future research focusing on malnutrition in the elderly is crucial, yet significant gaps exist in the current literature. Nations with lower healthcare capabilities face substantial challenges in diagnosis rates and epidemiological data collection, compounded by a lack of standardized diagnostic tools across nations. Additionally, there are insufficient epidemiological studies on different types of malnutrition, such as protein-energy malnutrition and micronutrient deficiencies. The biological mechanisms linking malnutrition and aging, as well as their relationship with multimorbidity, remain unclear. Furthermore, studies on the long-term effects and cost-effectiveness of nutritional interventions, such as supplementation and dietary adjustments, are limited, particularly in resource-constrained areas. The impacts of social isolation, economic hardship, and mental health issues, such as depression, have also been underexplored. Therefore, future research should focus on developing standardized diagnostic tools while enhancing international collaboration and data integration. It should also explore the biological mechanisms linking malnutrition and aging, assess the interactions between malnutrition and multimorbidity along with their health implications, and evaluate the long-term effects and cost-effectiveness of nutritional interventions.

This study also has several limitations. First, the GBD research primarily relies on modeling data, which may introduce inherent biases due to varying data collection methods, techniques, and tools utilized by different countries. This data heterogeneity may skew global comparisons, particularly underestimating the burden of malnutrition in low SDI countries. Second, the diagnosis rate of malnutrition varies significantly based on the level of development and healthcare infrastructure in each country. Countries with lower healthcare capacity may experience underdiagnosis, obscuring the true burden of malnutrition and leading to misguided policy decisions and resource allocation, thereby worsening nutritional issues among the elderly. It is essential to enhance resource support for low-income countries and promote standardized diagnostic tools for malnutrition. Additionally, while this study focuses exclusively on malnutrition, it is important to note that malnutrition encompasses various forms. The GBD database lacks comprehensive prevalence data for other subtypes, limiting our ability to report on specific forms of malnutrition. Despite these limitations, the study offered several advantages. We investigated the incidence, prevalence, and burden of malnutrition among individuals aged 70 and older across 204 countries and regions over the past 32 years. Additionally, we predicted the number of new cases of malnutrition in this age group globally over the next 25 years. These findings contributed to a deeper understanding of the epidemiology of malnutrition in older adults in different countries, providing guidance for the development of personalized health policies to address varying disease burdens worldwide. Furthermore, exploring the relationship between disease burden and the SDI may aid in the rational allocation of limited medical resources, which is crucial for promoting global health.

## 5 Conclusion

Malnutrition among the elderly represents a significant public health challenge that necessitates global attention. Despite a decline in both the prevalence rate of malnutrition and associated DALYs rate among individuals aged 70 and older from 1990 to 2021, the total number of affected elderly individuals and the overall burden remain alarmingly high. Given ongoing population growth and demographic aging, the incidence cases of malnutrition among the elderly are projected to rise further. Notably, while the prevalence rate of malnutrition is biased toward less developed regions, its incidence rate is also increasing in areas with advanced socio-demographic development. Consequently, it is imperative for countries to devise strategies tailored to their specific contexts. A multidisciplinary approach—integrating education, policy, agriculture, and healthcare—is essential to effectively address the underlying causes of malnutrition among the elderly. By implementing such strategies, we can mitigate the burden of malnutrition, enhance the health and well-being of older adults, and advance the goal of healthy aging.

## Data Availability

Publicly available datasets were analyzed in this study. This data can be found here: The data utilized in this study can be found in the Global Burden of Diseases, Injuries, and Risk Factors Study 2021 (https://ghdx.healthdata.org/gbd-2021). Some demographic data and Human Development Index data sources have been described in the Supplementary material.

## Footnotes

^1^ https://vizhub.healthdata.org/gbd-results/

## References

[1] World Health Organization. Ageing and health. (2024). Available online at: https://www.who.int/news-room/fact-sheets/detail/ageing-and-health.

[2] Beard JR Officer A de Carvalho IA Sadana R Pot AM Michel JP. The world report on ageing and health: a policy framework for healthy ageing. Lancet. (2016) 387:2145–54. doi: 10.1016/S0140-6736(15)00516-4, PMID: 26520231 PMC4848186

[3] Nations TU. The United Nations decade of action on nutrition. (2016). Available online at: https://www.un.org/nutrition/zh/about.

[4] World Health Organization. Malnutrition. (2024). Available online at: https://www.who.int/news-room/questions-and-answers/item/malnutrition.

[5] Dent E Wright ORL Woo J Hoogendijk EO. Malnutrition in older adults. Lancet. (2023) 401:951–66. doi: 10.1016/S0140-6736(22)02612-5, PMID: 36716756

[6] Newberry C Dakin G. Nutrition and weight management in the elderly. Clin Geriatr Med. (2021) 37:131–40. doi: 10.1016/j.cger.2020.08.010, PMID: 33213767

[7] Calcaterra L Abellan van Kan G Steinmeyer Z Angioni D Proietti M Sourdet S. Sarcopenia and poor nutritional status in older adults. Clin Nutr. (2024) 43:701–7. doi: 10.1016/j.clnu.2024.01.028, PMID: 38320461

[8] Griffiths J Seesen M Sirikul W Siviroj P. Malnutrition, depression, poor sleep quality, and difficulty falling asleep at night are associated with a higher risk of cognitive frailty in older adults during the COVID-19 restrictions. Nutrients. (2023) 15:2849. doi: 10.3390/nu15132849, PMID: 37447178 PMC10343894

[9] Wang K Zhao Y Nie J Xu H Yu C Wang S. Higher HEI-2015 score is associated with reduced risk of depression: result from NHANES 2005-2016. Nutrients. (2021) 13:348. doi: 10.3390/nu13020348, PMID: 33503826 PMC7911826

[10] Ahmed T Haboubi N. Assessment and management of nutrition in older people and its importance to health. Clin Interv Aging. (2010) 5:207–16. doi: 10.2147/cia.s9664, PMID: 20711440 PMC2920201

[11] Yu Y Li H Hu NX Wu XH Huang XY Lin HT. Global burden and health inequality of nutritional deficiencies from 1990 to 2019. Front Nutr. (2024) 11:1470713. doi: 10.3389/fnut.2024.1470713, PMID: 39385781 PMC11461340

[12] Liu Z Duan Y Yang L Du J Liu H. Global burden of childhood nutritional deficiencies, 1990-2019. Public Health. (2024) 235:26–32. doi: 10.1016/j.puhe.2024.06.027, PMID: 39038426

[13] Mao C Shen Z Long D Liu M Xu X Gao X. Epidemiological study of pediatric nutritional deficiencies: an analysis from the global burden of disease study 2019. Nutr J. (2024) 23:44. doi: 10.1186/s12937-024-00945-1, PMID: 38637763 PMC11027389

[14] Liu R Pi L Leng F Shen Q. Global disability-adjusted life years and deaths attributable to child and maternal malnutrition from 1990 to 2019. Front Public Health. (2024) 12:1323263. doi: 10.3389/fpubh.2024.1323263, PMID: 38304181 PMC10830744

[15] Woldekidan MA Arja A Worku G Walker A Kassebaum NJ Hailemariam A. The burden and trends of child and maternal malnutrition across the regions in Ethiopia, 1990-2019: the global burden of disease study 2019. PLoS Glob Public Health. (2024) 4:e0002640. doi: 10.1371/journal.pgph.0002640, PMID: 39012910 PMC11251601

[16] Liu J Qi X Wang X Qin Y Jiang S Han L. Evolving patterns of nutritional deficiencies burden in low- and middle-income countries: findings from the 2019 global burden of disease study. Nutrients. (2022) 14:931. doi: 10.3390/nu14050931, PMID: 35267908 PMC8912291

[17] Collaborators GBDRF. Global burden and strength of evidence for 88 risk factors in 204 countries and 811 subnational locations, 1990-2021: a systematic analysis for the global burden of disease study 2021. Lancet. (2024) 403:2162–203. doi: 10.1016/S0140-6736(24)00933-4, PMID: 38762324 PMC11120204

[18] Collaborators GBDF. Burden of disease scenarios for 204 countries and territories, 2022-2050: a forecasting analysis for the global burden of disease study 2021. Lancet. (2024) 403:2204–56. doi: 10.1016/S0140-6736(24)00685-8, PMID: 38762325 PMC11121021

[19] Collaborators GBDCoD. Global burden of 288 causes of death and life expectancy decomposition in 204 countries and territories and 811 subnational locations, 1990-2021: a systematic analysis for the global burden of disease study 2021. Lancet. (2024) 403:2100–32. doi: 10.1016/S0140-6736(24)00367-2, PMID: 38582094 PMC11126520

[20] Global Burden of Disease Study (2019). Global burden of disease study 2019 (GBD 2019) socio-demographic index (SDI) 1950–2019. Available online at: https://ghdx.healthdata.org/record/ihme-data/gbd-2019-socio-demographic-index-sdi-1950-2019.

[21] Software JTA. Version 4.9.1.0-April 2022; surveillance research program, division of cancer control & population sciences, National Cancer Institute 2022. (2023) Available online at: https://surveillance.cancer.gov/joinpoint/.

[22] Institute for Health Metrics and Evaluation (IHME). Global fertility, mortality, migration, and population forecasts 2017-2100. (2024)

[23] Moller B Fekjær H Hakulinen T Sigvaldason H Storm HH Talbäck M. Prediction of cancer incidence in the Nordic countries: empirical comparison of different approaches. Stat Med. (2003) 22:2751–66. doi: 10.1002/sim.1481, PMID: 12939784

[24] UN-Nutrition. UN-nutrition strategy 2022−2030: one UN for nutrition. (2022) Available online at: https://www.unnutrition.org/library/publication/un-nutrition-strategy-2022-2030-one-un-nutrition.

[25] Zarulli V Kashnitsky I Vaupel JW. Death rates at specific life stages mold the sex gap in life expectancy. Proc Natl Acad Sci USA. (2021) 118:e2010588118. doi: 10.1073/pnas.2010588118, PMID: 33972417 PMC8157960

[26] Heavey P. Conference on 'Understanding the role of sex and gender in nutrition research'. Proc Nutr Soc. (2024) 83:63–5. doi: 10.1017/S0029665123003749, PMID: 38347816

[27] Kantor ED Rehm CD Du M White E Giovannucci EL. Trends in dietary supplement use among US adults from 1999-2012. JAMA. (2016) 316:1464–74. doi: 10.1001/jama.2016.14403, PMID: 27727382 PMC5540241

[28] Gahche JJ Bailey RL Potischman N Dwyer JT. Dietary supplement use was very high among older adults in the United States in 2011-2014. J Nutr. (2017) 147:1968–76. doi: 10.3945/jn.117.255984, PMID: 28855421 PMC5610553

[29] Wells JC Sawaya AL Wibaek R Mwangome M Poullas MS Yajnik CS. The double burden of malnutrition: aetiological pathways and consequences for health. Lancet. (2020) 395:75–88. doi: 10.1016/S0140-6736(19)32472-9, PMID: 31852605 PMC7613491

[30] Allard L Ouedraogo E Molleville J Bihan H Giroux-Leprieur B Sutton A. Malnutrition: percentage and association with prognosis in patients hospitalized for coronavirus disease 2019. Nutrients. (2020) 12:3679. doi: 10.3390/nu12123679, PMID: 33260603 PMC7761464

[31] Bedock D Bel Lassen P Mathian A Moreau P Couffignal J Ciangura C. Prevalence and severity of malnutrition in hospitalized COVID-19 patients. Clin Nutr ESPEN. (2020) 40:214–9. doi: 10.1016/j.clnesp.2020.09.018, PMID: 33183539 PMC7500887

[32] Damayanthi H Prabani KIP. Nutritional determinants and COVID-19 outcomes of older patients with COVID-19: a systematic review. Arch Gerontol Geriatr. (2021) 95:104411. doi: 10.1016/j.archger.2021.104411, PMID: 33836322 PMC8010373

[33] Organization WH. The state of food security and nutrition in the world 2023. (2023). Available online at: https://www.who.int/publications/m/item/the-state-of-food-security-and-nutrition-in-the-world-2023.

[34] Programme WF. Supporting MALNUTRITION PREVENTION activities for VULNERABLE GROUPS. (2019).

[35] Adeyeye SAO Ashaolu TJ Bolaji OT Abegunde TA Omoyajowo AO. Africa and the Nexus of poverty, malnutrition and diseases. Crit Rev Food Sci. (2023) 63:641–56. doi: 10.1080/10408398.2021.1952160, PMID: 34259104

[36] Group TGNRS. Executive summary – global nutrition report 2022 (2022). Available online at: https://globalnutritionreport.org/a075dc#section-1.

[37] McMahon K Gray C. Climate change, social vulnerability and child nutrition in South Asia. Glob Environ Change. (2021) 71:102414. doi: 10.1016/j.gloenvcha.2021.102414, PMID: 34898861 PMC8653856

[38] Galicia L de Romana DL Harding KB De-Regil LM Grajeda R. Tackling malnutrition in Latin America and the Caribbean: challenges and opportunities. Rev Panam Salud Publica. (2016) 40:138–46. PMID: 27982371

[39] Organization of American States OAS. Additional protocol to the American convention on human rights in the area of economic, social and cultural rights (protocol of San Salvador), 14 November 1988. Annu Rev Popul Law. (1989) 16:234–8. 12344012

[40] Tirado MC Galicia L Husby HM Lopez J Olamendi S Pia Chaparro M. Mapping of nutrition and sectoral policies addressing malnutrition in Latin America. Rev Panam Salud Publica. (2016) 40:114–23. PMID: 27982369

[41] Sanchez-Romero LM Canto-Osorio F Gonzalez-Morales R Colchero MA Ng SW Ramirez-Palacios P. Association between tax on sugar sweetened beverages and soft drink consumption in adults in Mexico: open cohort longitudinal analysis of health workers cohort study. BMJ. (2020) 369:m1311. doi: 10.1136/bmj.m1311, PMID: 32376605 PMC7201935

[42] Quintiliano Scarpelli D Pinheiro Fernandes AC Rodriguez Osiac L Pizarro QT. Changes in nutrient declaration after the food labeling and advertising law in Chile: a longitudinal approach. Nutrients. (2020) 12:2371. doi: 10.3390/nu12082371, PMID: 32784370 PMC7468860

[43] Reyes M Garmendia ML Olivares S Aqueveque C Zacarias I Corvalan C. Development of the Chilean front-of-package food warning label. BMC Public Health. (2019) 19:906. doi: 10.1186/s12889-019-7118-1, PMID: 31286910 PMC6615240

[44] Muennig PA Glied SA. What changes in survival rates tell us about us health care. Health Aff (Millwood). (2010) 29:2105–13. doi: 10.1377/hlthaff.2010.0073, PMID: 20930036

[45] Chambers R Bryan J Jannat-Khah D Russo E Merriman L Gupta R. Evaluating gaps in Care of Malnourished Patients on general medicine floors in an acute care setting. Nutr Clin Pract. (2019) 34:313–8. doi: 10.1002/ncp.10097, PMID: 29701888

[46] Mostafa N Sayed A Rashad O Baqal O. Malnutrition-related mortality trends in older adults in the United States from 1999 to 2020. BMC Med. (2023) 21:421. doi: 10.1186/s12916-023-03143-8, PMID: 37936140 PMC10631109

[47] Bailey RL Dog TL Smith-Ryan AE Das SK Baker FC Madak-Erdogan Z. Sex differences across the life course: a focus on unique nutritional and health considerations among women. J Nutr. (2022) 152:1597–610. doi: 10.1093/jn/nxac059, PMID: 35294009 PMC9258555

[48] Roberts SB Silver RE Das SK Fielding RA Gilhooly CH Jacques PF. Healthy aging-nutrition matters: start early and screen often. Adv Nutr. (2021) 12:1438–48. doi: 10.1093/advances/nmab032, PMID: 33838032 PMC8994693

[49] Cederholm T Barazzoni R Austin P Ballmer P Biolo G Bischoff SC. ESPEN guidelines on definitions and terminology of clinical nutrition. Clin Nutr. (2017) 36:49–64. doi: 10.1016/j.clnu.2016.09.004, PMID: 27642056

[50] Schuetz P Seres D Lobo DN Gomes F Kaegi-Braun N Stanga Z. Management of disease-related malnutrition for patients being treated in hospital. Lancet. (2021) 398:1927–38. doi: 10.1016/S0140-6736(21)01451-3, PMID: 34656286

[51] Dent E Hoogendijk EO Wright ORL. New insights into the anorexia of ageing: from prevention to treatment. Curr Opin Clin Nutr. (2019) 22:44–51. doi: 10.1097/MCO.0000000000000525, PMID: 30394894

[52] Favaro-Moreira NC Krausch-Hofmann S Matthys C Vereecken C Vanhauwaert E Declercq A. Risk factors for malnutrition in older adults: a systematic review of the literature based on longitudinal data. Adv Nutr. (2016) 7:507–22. doi: 10.3945/an.115.011254, PMID: 27184278 PMC4863272

[53] Snider JT Linthicum MT Wu Y LaVallee C Lakdawalla DN Hegazi R. Economic burden of community-based disease-associated malnutrition in the United States. JPEN J Parenter Enteral Nutr. (2014) 38:77S–85S. doi: 10.1177/0148607114550000 25249028

