# Supplementary material for: Global patterns of change in the burden of malnutrition in older adults from 1990 to 2021 and the forecast for the next 25 years
Source: Front Nutr. 2025 Mar 20;12:1562536. doi: 10.3389/fnut.2025.1562536 (PMC11965120; doi:10.3389/fnut.2025.1562536)
Supplement: Supplementary file 1 [file Presentation_1.pdf]

Global Patterns of Change in the Burden of Malnutrition in Older Adults from 1990 to 2021 and the Forecast for the Next 25 Years

Le Li[1,2], Xiao Liu[1,2], Yujie Fang[1,2], Kailin Guo[1,2], Lu Li[1,2], Shuhan Cai[1,2], Chang Hu[1,2*], Bo Hu[1,2*]

[1] Department of Critical Care Medicine, Zhongnan Hospital of Wuhan University, Wuhan 430071, Hubei, China

[2] Clinical Research Center of Hubei Critical Care Medicine, Wuhan 430071, Hubei, China

# Le Li and Xiao Liu contribute equally to the article.

Correspondence to: Bo Hu, MD; Zhongnan Hospital of Wuhan University; Wuhan 430071, Hubei, China; Email: hubozn@whu.edu.cn

And Correspondence to: Chang Hu, MD; Zhongnan Hospital of Wuhan University; Wuhan 430071, Hubei, China; Email: huchang@whu.edu.cn

Supplementary Material

- **eMethods**
- **eTable 1:** List of International Classification of Diseases (ICD) codes mapped to the Global Burden of Nutritional deficiencies list
- **eTable 2:** The incidence cases and incidence of malnutrition among individuals over 70 in 1990 and 2021, and its temporal trends from 1990 to 2021
- **eTable 3:** The DALYs number and DALYs rate of malnutrition among individuals over 70 in 1990 and 2021, and its temporal trends from 1990 to 2021
- **eTable 4:** The prevalence cases and prevalence of malnutrition among individuals over 70 in 1990 and 2021, and its temporal trends from 1990 to 2021, at the national level
- **eTable 5:** The incidence cases and incidence of malnutrition among individuals over 70 in 1990 and 2021, and its temporal trends from 1990 to 2021, at the national level
- **eTable 6:** The DALYs number and DALYs rate of malnutrition among individuals over 70 in 1990 and 2021, and its temporal trends from 1990 to 2021, at the national level
- **eTable 7:** Changes in prevalence cases of malnutrition in overall population at all ages at according to population-level determinants including aging, population growth and epidemiological change from 1990 to 2019 at the global level and by SDI quintiles stratified by sexes

- **eTable 8:** Changes in global incidence cases and incidence over the next 25 years, based on Nordpred
- **eTable 9:** The population of individuals over 70 and overall population in 1990 and 2021
- **eTable 10:** The prevalence cases and prevalence of malnutrition among individuals over 70 and overall population in 1990 and 2021
- **eFigure 1:** The study flowchart of worldwide burden of nutritional deficiencies in individuals aged 70 and older from 1990 to 2021.
- **eFigure 2:** Joinpoint regression analysis of prevalence for nutritional deficiency in the elderly from 1990 to 2021. (A) Global; (B) high SDI; (C) high-middle SDI; (D) middle SDI; (E) low-middle SDI; (F) low SDI.
- **eFigure 3.** Prevalence (A) and DALYs rate (B)of malnutrition among individuals over 70 by year and sex.
- **eFigure 4:** Prevalence (A) and DALYs rate (B)of malnutrition among individuals over 70 by year and regions with 95% uncertainty intervals
- **eFigure 5:** (A) The incidence of malnutrition among individuals over 70 in 2021. (B) The relative change in case number of incidence of malnutrition among individuals over 70 from 1990 to 2021. (C) The EAPC of incidence of malnutrition among individuals over 70 from 1990 to 2021.
- **eFigure 6:** (A) The DALYs rate of malnutrition among individuals over 70 in 2021. (B) The relative change in case number of DALYs of malnutrition among

individuals over 70 from 1990 to 2021. (C) The EAPC of DALYs rate of malnutrition among individuals over 70 from 1990 to 2021.

- **eFigure 7.** (A) DALYs rates for malnutrition among individuals over 70 for 21 GBD regions by Socio-demographic Index, 1990–2021.Expected values based on Socio-demographic Index and disease rates in all locations are shown as the black line. The correlation between EAPC and (B) malnutrition incidence among individuals over 70 in 1990 and (C) HDI in 2022. The circles represent countries that were available on HDI data. The size of circle is increased with the cases of malnutrition. The ρ indices and p values presented in (B) and (C) were derived from Pearson correlation analysis.
- **eFigure 8:** DALYs rates for malnutrition among individuals over 70 for 204 countries and territories by Socio-demographic Index, 1990–2021.Expected values based on Socio-demographic Index and disease rates in all locations are shown as the black line.

eMethods

Data Sources

Data sources can be found through the GBD 2021 Data Input Sources Tool on the Institute for Health Metrics and Evaluation (IHME) website.
The SDI values for each country and region for various years are published on the IHME website: (https://ghdx.healthdata.org/record/ihme-data/gbd-2019-socio-demographic-index-sdi-1950-2019).
The Human Development Index (HDI) for each country in 2022 can be obtained from the United Nations Development Programme (UNDP) Human Development Report website: [https://hdr.undp.org/data-center/human-development-index#/indicies/HDI]
Future population data projections are sourced from a paper published in The Lancet titled "Fertility, mortality, migration, and population scenarios for 195 countries and territories from 2017 to 2100: a forecasting analysis for the Global Burden of Disease Study," which includes the forecast results in its appendix.

Search Methods

The data was downloaded from the Global Health Data Exchange (GHDx) query tool. Specifically, in the GHDx query tool, we selected "Cause" as "Nutritional Deficiencies," "Measure" as "Prevalence," "Incidence," and "Disability-adjusted life years (DALYs)," and "Metric" as "Number" and "Rate." This provided us with prevalence data for 204 countries and regions worldwide, covering the period from 1990 to 2021.

Joinpoint regression analysis

The Joinpoint model is a statistical method used to analyze trends in data over time. It identifies points, known as "joinpoints," where the trend of the data significantly changes. The model divides the data into segments based on identified joinpoints. Each segment represents a period during which the trend is relatively stable. For each segment, a linear regression is performed to estimate the slope (rate of change) of the trend. This helps in quantifying how the trend behaves in each segment. The model uses statistical tests to determine whether a joinpoint exists. It compares models with different numbers of joinpoints to find the best-fitting model.
The analysis was performed utilizing the ‘Joinpoint’ software supplied by the Surveillance Research Program of the US National Cancer Institute

EAPC Calculation Method

The Estimated Annual Percentage Change (EAPC) is calculated based on a regression model fitted to the natural logarithm of the annual incidence rate of malnutrition over calendar years. The model is given by:

$$Y = \alpha + \beta X + \epsilon$$

$$\text{EAPC} = 100 \times (\exp(\beta) - 1)$$

x- year, y- the natural logarithm of rates (such as prevalence and incidence), α- the intercept,β- the slope, ε- the random error. The 95% confidence intervals (CIs) of the EAPC are also derived from this fitted model. The interpretation of trend results is based on the 95% CIs; a lower limit of 95% CIs greater than 0 indicates an upward trend, while an upper limit of 95% CIs lower than 0 suggests a downward trend. If the 95% CIs include 0, it indicates that there is no statistically significant difference in trend changes.

SDI

The Socio-demographic Index (SDI) is a composite measure used to classify countries into development groups based on three key indicators: total fertility rate under age 25 (TFU25), mean years of education for those aged 15 and older (EDU15+), and lagged distribution income per capita (LDI). These indicators are rescaled from 0 to 1 and combined into an overall SDI score ranging from 0 (lowest development) to 1 (highest development).

eTable 1. List of International Classification of Diseases (ICD) codes mapped to the Global Burden of Nutritional deficiencies list

Cause	ICD10	ICD10 Used in Hospital/Claims Analyses	ICD9	ICD9 Used in Hospital/Claims Analyses
Nutritional deficiencies	D50-D53.9, E00-E02, E40-E46.9, E50-E61.9, E63-E64.9, Z13.2-Z13.3	E01-E02, E50.0-E50.7	244.2, 260-269.9, 280-281.2, V12.1, V18.2-V18.3, V77.2, V78.0-V78.1	244.2, 264.0-264.6

eTable 2. The incidence cases and incidence of malnutrition among individuals over 70 in 1990 and 2021, and its temporal trends from 1990 to 2021

Location	1990		2021		1990-2021
	**Case number (95% UI)**	**Rate (95% UI)**	**Case number (95% UI)**	**Rate (95% UI)**	**EAPC% (95% CI)**
Global	15341545.1 (13985419.4-16709385.1)	7594.6 (6923.2-8271.7)	20773129.2 (18756849-23021934.1)	4202 (3794.1-4656.8)	-1.82 (-1.91--1.73)
High SDI	1617224 (1367324.4-1901114.3)	2341.9 (1980-2753)	3369997.4 (2906424.9-3942229.7)	2348.7 (2025.7-2747.6)	0.20 (0.03-0.37)
High-middle SDI	2058632.3 (1890933.4-2254357.5)	4000.3 (3674.4-4380.6)	3633383.1 (3216388.3-4116576.8)	3094.8 (2739.6-3506.4)	-0.64 (-0.74--0.53)
Middle SDI	2891297.2 (2618325.8-3219881.4)	6327.4 (5730-7046.5)	5171787.4 (4560011.2-5830012)	3668.1 (3234.2-4134.9)	-1.54 (-1.64--1.45)
Low-middle SDI	5351166.5 (4672338.9-6161720.7)	20405.4 (17816.8-23496.2)	5239640.6 (4510853.6-6115497.3)	7475 (6435.3-8724.6)	-3.34 (-3.47--3.21)
Low SDI	3401816.7 (3195052.2-3633982.5)	36452.8 (34237.2-38940.6)	3333947.2 (3039302.1-3713737.6)	15200 (13856.7-16931.5)	-2.90 (-3.14--2.66)
Andean Latin America	45607.4 (37366.3-56584.7)	4469.3 (3661.7-5545.1)	116846.3 (97288.8-138138.9)	3562 (2965.8-4211.1)	-1.13 (-1.48--0.77)
Australasia	22220.1 (18557.9-26506.3)	1524.7 (1273.4-1818.8)	59862.9 (52205.6-70715.4)	1643.1 (1432.9-1940.9)	0.37 (0.21-0.52)
Caribbean	88161 (79053.3-97856)	5972.4 (5355.4-6629.1)	121426.4 (108302.9-137191.7)	3795.2 (3385-4287.9)	-1.75 (-1.94--1.56)
Central Asia	136419 (119809.7-156966.1)	6054.3 (5317.1-6966.1)	132815.1 (116424-151656.6)	3907.3 (3425.1-4461.6)	-1.42 (-1.58--1.25)
Central Europe	879254.5 (786654.2-987564.3)	11131 (9958.8-12502.2)	990797.8 (889138.4-1104643.4)	6674.5 (5989.7-7441.4)	-1.82 (-2.03--1.61)
Central Latin America	325483.8 (292990-360089.8)	8057 (7252.7-8913.7)	599678.5 (542307-664308.5)	4389.6 (3969.7-4862.7)	-1.80 (-1.94--1.67)
Central Sub-Saharan Africa	202265.6 (165246.4-240806.7)	24952.5 (20385.7-29707.2)	289813.7 (239031.2-348951.1)	15371.7 (12678.2-18508.3)	-1.47 (-1.98--0.95)
East Asia	1233734.6 (1001462.2-1537729.5)	3170.4 (2573.5-3951.6)	3328303.4 (2757837.4-4033781.7)	2693.2 (2231.6-3264.1)	-0.02 (-0.31-0.28)
Eastern Europe	193247.2 (161609.7-230786.2)	1275.6 (1066.7-1523.4)	206781 (170771.6-249521.7)	968 (799.4-1168.1)	-0.92 (-1.01--0.82)
Eastern Sub-Saharan Africa	1497263.3 (1409557.8-1580578.5)	48074.3 (45258.3-50749.4)	1436762.3 (1323390.4-1573709.7)	20852.3 (19206.8-22839.8)	-2.88 (-3.10--2.67)
High-income Asia Pacific	183902.7 (147642.2-229372.7)	1633.9 (1311.7-2037.8)	565288.4 (475347.8-683339.3)	1617.9 (1360.5-1955.8)	-0.23 (-0.44--0.03)
High-income North America	521873.6 (402026.2-680009.1)	2247.3 (1731.2-2928.3)	1005923.3 (811549.5-1246740.9)	2322.7 (1873.9-2878.7)	0.23 (0-0.46)

North Africa and Middle East	928767.8<br>(857949.1-1013274.2)	12860.9<br>(11880.2-14031.1)	853698<br>(779936.1-938708.2)	4198.4<br>(3835.6-4616.4)	-3.47<br>(-3.53--3.41)
Oceania	11763.7<br>(9898.6-13958)	11315.6<br>(9521.5-13426.4)	15738.9<br>(13164.8-18745.2)	5705.5<br>(4772.3-6795.3)	-2.03<br>(-2.11--1.96)
South Asia	5136461.2<br>(4170686.2-6301938.8)	21868.7<br>(17756.9-26830.8)	5494026.4<br>(4413292.5-6835217)	7504<br>(6027.9-9335.9)	-3.62<br>(-3.82--3.43)
Southeast Asia	845380.9<br>(726262.1-977868.7)	7743.8<br>(6652.6-8957.4)	1012135.8<br>(906974.1-1133601.7)	3365.1<br>(3015.4-3768.9)	-2.61<br>(-2.94--2.28)
Southern Latin America	155423.1<br>(123288.4-193132.8)	5898.1<br>(4678.6-7329.1)	280081.8<br>(228199.5-334190.2)	5094.8<br>(4151-6079.1)	-0.61<br>(-1.01--0.21)
Southern Sub-Saharan Africa	59162.6<br>(50883.5-69065.3)	4557.4<br>(3919.7-5320.3)	71131.3<br>(60897.2-83021.7)	2667.2<br>(2283.4-3113)	-1.51<br>(-1.63--1.39)
Tropical Latin America	848958.8<br>(663237.4-1053838.6)	19443.5<br>(15190-24135.8)	1364988<br>(1106024.8-1712821)	9503.3<br>(7700.3-11924.9)	-2.21<br>(-2.31--2.12)
Western Europe	918299.8<br>(808284.6-1035775.6)	2458.9<br>(2164.3-2773.5)	1856790.4<br>(1593738.5-2188929.7)	2822<br>(2422.2-3326.8)	0.97<br>(0.76-1.17)
Western Sub-Saharan Africa	1107894.6<br>(1036729.3-1195427.3)	27567.2<br>(25796.4-29745.2)	970239.4<br>(894494.8-1050417.5)	11905.2<br>(10975.8-12889)	-2.63<br>(-2.73--2.54)

Abbreviations: EAPC, estimated annual percentage change.

eTable 3. The DALYs number and DALYs rate of malnutrition among individuals over 70 in 1990 and 2021, and its temporal trends from 1990 to 2021

Location	1990		2021		1990-2021
	Case number (95% UI)	Rate (95% UI)	Case number (95% UI)	Rate (95% UI)	EAPC% (95% CI)
Global	1881624.9 (1612399-2215582.9)	931.5 (798.2-1096.8)	3229809.3 (2607370-4002270.6)	653.3 (527.4-809.6)	-1.31 (-1.45--1.16)
High SDI	136474.8 (112957.3-171934.9)	197.6 (163.6-249)	432461.9 (355022-519832.7)	301.4 (247.4-362.3)	1.34 (1.14-1.54)
High-middle SDI	204089.2 (161294.6-261076.8)	396.6 (313.4-507.3)	344619.2 (271671.5-443037.1)	293.5 (231.4-377.4)	-1.11 (-1.25--0.97)
Middle SDI	626785.7 (544510-729252.8)	1371.7 (1191.6-1595.9)	1023977.4 (838664.6-1256626.7)	726.3 (594.8-891.3)	-2.07 (-2.12--2.02)
Low-middle SDI	631212.3 (525778.3-757035.1)	2407 (2004.9-2886.8)	1016636.5 (790474.3-1289184.2)	1450.4 (1127.7-1839.2)	-2.11 (-2.47--1.76)
Low SDI	281830.5 (240732.4-325917.2)	3020 (2579.6-3492.4)	410212.3 (330886-511689.3)	1870.2 (1508.6-2332.9)	-1.60 (-1.76--1.44)
Andean Latin America	22894 (20323.5-25733.9)	2243.5 (1991.6-2521.8)	29130 (24451-34701.2)	888 (745.4-1057.9)	-3.26 (-3.47--3.05)
Australasia	1863.5 (1419.2-2390.3)	127.9 (97.4-164)	3974.6 (2906.9-5444)	109.1 (79.8-149.4)	-0.52 (-0.66--0.39)
Caribbean	8602.8 (7528.3-9986.3)	582.8 (510-676.5)	12693.1 (10556.3-15885.9)	396.7 (329.9-496.5)	-1.38 (-1.62--1.13)
Central Asia	7769.3 (5336.5-10820.8)	344.8 (236.8-480.2)	8048.2 (5408.5-11719)	236.8 (159.1-344.8)	-1.76 (-1.95--1.56)
Central Europe	18360.3 (12774.6-25171.1)	232.4 (161.7-318.7)	31065.6 (22892-41178.3)	209.3 (154.2-277.4)	-0.35 (-0.50--0.20)
Central Latin America	125158.9 (118419.5-129967.5)	3098.2 (2931.4-3217.2)	107542.3 (97428.2-118901.2)	787.2 (713.2-870.4)	-4.56 (-4.67--4.46)
Central Sub-Saharan Africa	19390.3 (15168.5-23685.3)	2392.1 (1871.3-2921.9)	26721.7 (20294.8-33905.4)	1417.3 (1076.4-1798.3)	-1.86 (-2.11--1.60)
East Asia	304497.3 (246158.6-380029.6)	782.5 (632.6-976.6)	443969 (344289.2-574066)	359.3 (278.6-464.5)	-3.63 (-4.36--2.89)
Eastern Europe	28544.8 (18857.8-41430.4)	188.4 (124.5-273.5)	35245 (24341.3-51237)	165 (114-239.9)	-0.70 (-0.86--0.54)
Eastern Sub-Saharan Africa	131811.6 (114998.5-149148.6)	4232.2 (3692.4-4788.9)	116040.2 (101005.1-133736)	1684.1 (1465.9-1941)	-3.18 (-3.59--2.77)
High-income Asia Pacific	18532.8 (14226.9-24665.4)	164.7 (126.4-219.1)	65608.7 (47025.8-91873.4)	187.8 (134.6-263)	0.46 (0.28-0.64)
High-income North America	42181.1 (34153.3-53935.1)	181.6 (147.1-232.3)	201738.6 (167097.8-236522.8)	465.8 (385.8-546.1)	2.73 (2.41-3.05)
North Africa and Middle East	49112.4 (39333.8-61928)	680.1 (544.7-857.5)	84400.7 (65426-111586.6)	415.1 (321.8-548.8)	-1.51 (-1.66--1.36)

Oceania	1478.1<br>(1203.8-1842)	1421.8<br>(1158-1771.8)	3038.1<br>(2369.9-3888.5)	1101.3<br>(859.1-1409.6)	-0.70<br>(-0.75--0.65)
South Asia	653498.3<br>(523177.6-815167.4)	2782.3<br>(2227.5-3470.6)	1309972<br>(953873.5-1731491.6)	1789.2<br>(1302.8-2365)	-1.44<br>(-1.47--1.4)
Southeast Asia	259725.9<br>(221918.6-299821.8)	2379.1<br>(2032.8-2746.4)	402721.2<br>(344754.3-470663.4)	1338.9<br>(1146.2-1564.8)	-1.69<br>(-1.82--1.55)
Southern Latin America	14499.3<br>(12525.3-17114.2)	550.2<br>(475.3-649.5)	22774.4<br>(18474.8-27881.1)	414.3<br>(336.1-507.2)	-1.00<br>(-1.56--0.44)
Southern Sub-Saharan Africa	11046.2<br>(9123.7-13278.1)	850.9<br>(702.8-1022.8)	17705<br>(14792.2-20960.7)	663.9<br>(554.7-786)	-0.92<br>(-1.27--0.57)
Tropical Latin America	43947.2<br>(39126.9-50435.3)	1006.5<br>(896.1-1155.1)	72845.1<br>(62142-87869.7)	507.2<br>(432.6-611.8)	-2.02<br>(-2.29--1.75)
Western Europe	64431.3<br>(52759-79626.1)	172.5<br>(141.3-213.2)	155614<br>(118942-207387)	236.5<br>(180.8-315.2)	1.39<br>(1.22-1.55)
Western Sub-Saharan Africa	54279.5<br>(46441.4-64502.7)	1350.6<br>(1155.6-1605)	78962<br>(64147.7-97798.2)	968.9<br>(787.1-1200)	-1.00<br>(-1.05--0.94)

Abbreviations: EAPC: estimated annual percentage change; DALYs: disability-adjusted life years.

eTable 4. The prevalence cases and prevalence of malnutrition among individuals over 70 in 1990 and 2021, and its temporal trends from 1990 to 2021, at the national level

Abbreviations: EAPC, estimated annual percentage change.

Location	1990		2021		1990-2021
	Case number (95% UI)	Rate (95% UI)	Case number (95% UI)	Rate (95% UI)	EAPC% (95% CI)
Afghanistan	173239.6 (152585.7-197081.8)	55744.7 (49098.7-63416.6)	151356.5 (130406-177030.9)	37414.2 (32235.4-43760.7)	-1.37 (-1.58~-1.16)
Albania	25534.9 (22057.3-29950.8)	25579.8 (22096-30003.4)	51301.9 (44224.3-59556.8)	18917.3 (16307.5-21961.3)	-1.34 (-1.5~-1.19)
Algeria	158560.2 (132451.2-196149.9)	25459.6 (21267.3-31495.2)	364025.3 (301843.2-452830.1)	21039.7 (17445.7-26172.3)	-0.55 (-0.58~-0.53)
American Samoa	260.8 (213.7-322.8)	29704.2 (24343.2-36762.6)	692 (561.3-888.8)	31247.8 (25348-40137.2)	0.24 (0.21~0.27)
Andorra	189.7 (151.3-229.2)	5924.2 (4724.9-7156.2)	615.1 (467-795)	6590.4 (5004-8519)	0.58 (0.44~0.71)
Angola	58571.7 (51701.8-65464.2)	45671.1 (40314.3-51045.5)	112236.7 (98643.8-128648.3)	27969.1 (24581.8-32058.8)	-1.72 (-1.92~-1.53)
Antigua and Barbuda	603.7 (469.5-779.5)	16594.1 (12905.6-21426.4)	800.7 (611.1-1092.8)	14256.4 (10880.9-19457)	-0.56 (-0.66~-0.47)
Argentina	319635.8 (271916.6-376634.3)	17284.6 (14704.1-20366.8)	549621.5 (463963.5-638122.9)	15422.8 (13019.2-17906.3)	-0.32 (-0.48~-0.15)
Armenia	15933.3 (12368.5-19457.7)	13337.5 (10353.5-16287.7)	27297.7 (20640.3-34541.2)	11166 (8442.8-14128.9)	-1 (-1.15~-0.85)
Australia	84462.3 (66453.5-103090.5)	6980.7 (5492.3-8520.3)	233191.2 (189136.6-283149.2)	7561.5 (6133-9181.4)	0.37 (0.3~0.44)
Austria	53138.4 (44203.4-63405.3)	6969.9 (5798-8316.6)	78178.8 (61205.6-97604.1)	6284.8 (4920.3-7846.4)	-0.1 (-0.19~-0.01)
Azerbaijan	29632.3 (24419-35588.8)	13190.9 (10870.2-15842.5)	46961.1 (36384.6-60685.3)	12255.5 (9495.3-15837.1)	-0.48 (-0.73~-0.23)
Bahamas	1081.8 (769.9-1506.4)	13381.8 (9523.1-18633.3)	2347 (1678.1-3362.5)	12109.7 (8658.3-17349.1)	-0.43 (-0.52~-0.35)
Bahrain	1214.7 (998-1557.3)	21231.7 (17444.5-27221)	4413.4 (3470.5-5653)	17518.8 (13776-22438.9)	-0.62 (-0.7~-0.53)
Bangladesh	1084051.1 (1002951-1180549.8)	47644 (44079.7-51885.1)	2658876.7 (2365969.3-3090719.9)	36078.7 (32104.2-41938.5)	-0.86 (-0.89~-0.83)
Barbados	2755.9 (2159.4-3335.6)	12895.6 (10104.2-15608)	4113.6 (3177.3-5526.3)	12836.6 (9914.9-17245)	-0.09 (-0.16~-0.03)
Belarus	79778.1 (66711.1-97617)	11467.1 (9588.9-14031.2)	81516.3 (65922.8-100379.8)	8543.2 (6909-10520.2)	-1.14 (-1.22~-1.06)
Belgium	73917.6 (62850.9-85505.3)	7692.4 (6540.7-8898.3)	150924 (124719.5-189586)	9386.5 (7756.8-11791)	0.81 (0.74~0.89)
Belize	1164.4	22102.3	2498.1	17892.8	-0.69

	(955.4-1407.2)	(18136.7-26711.9)	(1877.4-3340)	(13447.2-23922.8)	(-0.75~-0.63)
Benin	54578.4	53204.4	80197.4	35833.4	-1.19
	(49353.8-59916.3)	(48111.3-58407.9)	(69810.8-90665.3)	(31192.5-40510.6)	(-1.22~-1.17)
Bermuda	322.2	9274.4	728.3	8084.1	-0.54
	(246.4-436.8)	(7092-12571)	(544.4-1055.2)	(6042.9-11712.2)	(-0.63~-0.45)
Bhutan	3863.3	43086.5	10566.3	32776.5	-0.95
	(3412-4343.2)	(38052.8-48438.4)	(9626.7-11655.9)	(29861.7-36156.3)	(-1.02~-0.89)
Bolivia	31925.9	21268.5	77864.3	16826.4	-0.84
(Plurinational State of)	(26277.8-38780.9)	(17505.8-25835.2)	(58939.3-102581.4)	(12736.7-22167.7)	(-0.91~-0.77)
Bosnia and	41504.2	24754.8	69466	17876.4	-1.64
Herzegovina	(35043.9-48385.8)	(20901.6-28859.3)	(58895.9-80336.5)	(15156.3-20673.9)	(-1.84~-1.44)
Botswana	5782.8	25175.8	9136.6	15604.3	-1.4
	(4817.7-6855.4)	(20974.3-29845.5)	(7585.1-11255.8)	(12954.6-19223.7)	(-1.46~-1.34)
Brazil	1310001.6	30821.2	2555603.9	18174.5	-1.64
	(1106777.1-1532698.6)	(26039.8-36060.8)	(2149563-3092903.4)	(15286.9-21995.6)	(-1.66~-1.62)
Brunei	610.4	13705.9	1474.6	10806.5	-0.77
Darussalam	(527.9-697.4)	(11853.1-15660.1)	(1200.9-1791.9)	(8800.5-13131.6)	(-0.81~-0.72)
Bulgaria	121050.5	18354.8	173670.6	16870.8	-0.19
	(102920.2-143909.5)	(15605.7-21820.9)	(151311-198746.8)	(14698.7-19306.7)	(-0.35~-0.03)
Burkina Faso	122850.9	62576.6	159052.6	37761.9	-1.63
	(111365.5-133563.7)	(56726.2-68033.3)	(135937.9-184070.4)	(32274.1-43701.6)	(-1.68~-1.59)
Burundi	65170.4	57598.2	76813.9	42640.4	-1.05
	(57747.7-72506.5)	(51037.9-64081.9)	(66274.8-88347.1)	(36790-49042.6)	(-1.12~-0.97)
Cabo Verde	5427.4	36298.1	4465.2	21799.1	-1.72
	(4863.5-6075.9)	(32526.6-40634.9)	(3816.8-5124)	(18633.9-25015.6)	(-1.85~-1.6)
Cambodia	66661.4	35745.6	134912.6	24661.4	-1.28
	(58917-76449.3)	(31592.9-40994.1)	(113625.2-165318.8)	(20770.2-30219.5)	(-1.35~-1.21)
Cameroon	118652.1	64449.6	160099.3	32001	-2.04
	(109642-128350.1)	(59555.5-69717.5)	(137953.1-185077.2)	(27574.4-36993.6)	(-2.25~-1.84)
Canada	124757.8	6202.8	317658.3	6592.1	0.16
	(103311.2-150180.8)	(5136.5-7466.8)	(249962.6-398241.1)	(5187.2-8264.3)	(0.04~0.27)
Central African	17414.9	46871.6	24139.2	37833.6	-0.7
Republic	(15014.8-19789.1)	(40411.9-53261.6)	(20592-27442.3)	(32273.9-43010.5)	(-0.74~-0.66)
Chad	101587.5	67450.5	106036.9	44493.4	-1.35
	(92617.2-109374.9)	(61494.6-72621.1)	(93124.2-121241.2)	(39075.2-50873.2)	(-1.41~-1.3)
Chile	70111.8	12976.4	172549	11077.6	-0.58
	(58332.1-85406.3)	(10796.2-15807.2)	(142907-210504.9)	(9174.6-13514.3)	(-0.62~-0.54)
China	7703528.3	20526.5	16894046.6	14160.5	-1.3
	(7340066.4-8111876.4)	(19558.1-21614.6)	(15955653.6-17938952.4)	(13373.9-15036.3)	(-1.37~-1.23)
Colombia	131437.8	15713	293950.4	9224.1	-1.74
	(114168.2-154207.3)	(13648.4-18435)	(248787-342641.7)	(7806.9-10752)	(-1.77~-1.72)
Comoros	4251.9	52477.2	7449.3	32831.4	-1.5
	(3691.5-4779.2)	(45560.5-58985.1)	(6470-8578)	(28515.2-37805.9)	(-1.56~-1.44)

Congo	19675.3 (17222-22146.5)	45124.5 (39497.9-50792.2)	37599.5 (33023.6-42751)	39095.8 (34337.8-44452.3)	-0.26 (-0.53~0)
Cook Islands	161.1 (133.9-196)	27394.4 (22764.3-33315.2)	379.5 (283.3-496.6)	24366.9 (18189.4-31890.4)	-0.32 (-0.37~-0.26)
Costa Rica	12584.5 (10812.2-14818.1)	13285.6 (11414.6-15643.7)	30392 (25613.2-35669.5)	9691.3 (8167.4-11374.2)	-1.01 (-1.04~-0.97)
Côte d’Ivoire	46560.2 (39956.4-53911.5)	34797.3 (29861.9-40291.3)	109346.5 (92878.2-131080.1)	26224.6 (22275-31437)	-0.67 (-0.78~-0.56)
Croatia	46688.7 (39419.6-56372)	16472 (13907.4-19888.3)	81720.4 (69934.6-95762.4)	13291.6 (11374.7-15575.5)	-1.01 (-1.14~-0.88)
Cuba	71425.2 (54841.5-97566.8)	11414.3 (8764.1-15591.9)	139594.5 (107945.8-191046.8)	11241.8 (8693.1-15385.4)	-0.18 (-0.27~-0.09)
Cyprus	4243.7 (3341-5427.6)	8229 (6478.4-10524.7)	8483.4 (7060.3-9976.1)	6266.2 (5215-7368.8)	-0.79 (-0.9~-0.67)
Czechia	125685.4 (105178-150498)	15770 (13196.9-18883.3)	201424.8 (173888.9-231455.2)	13042.4 (11259.4-14986.9)	-0.34 (-0.51~-0.17)
Democratic People's Republic of Korea	137703.1 (120815.7-155989.1)	21031.1 (18451.9-23823.9)	319002.9 (269922.8-371796.5)	18228 (15423.5-21244.6)	-1.75 (-2.18~-1.31)
Democratic Republic of the Congo	286078.1 (254439.5-317853.7)	50555.3 (44964.2-56170.7)	512220 (451345.9-570001.4)	40509.5 (35695.2-45079.2)	-0.54 (-0.85~-0.24)
Denmark	38445.4 (30860.4-49102.2)	6877.6 (5520.7-8784)	58576.4 (46604.3-73370)	6813.7 (5421.1-8534.5)	0.02 (-0.13~0.18)
Djibouti	2337.5 (2107.4-2552.1)	54997.9 (49584.2-60047.4)	8882.1 (7881.3-9992.9)	40556.2 (35986.4-45628)	-1.03 (-1.16~-0.9)
Dominica	668.5 (528.6-862)	17334.3 (13707-22352.8)	690.4 (520.8-920)	15299.7 (11540.3-20385.8)	-0.42 (-0.46~-0.37)
Dominican Republic	45355.9 (37307.3-54809.2)	24598.7 (20233.5-29725.7)	91485.6 (71179.5-120695.2)	16486.4 (12827.1-21750.1)	-1.27 (-1.4~-1.14)
Ecuador	28411.3 (19671.2-43154.8)	10491.2 (7263.8-15935.4)	66633.6 (46890-106826.4)	7127.4 (5015.5-11426.6)	-1.06 (-1.17~-0.96)
Egypt	270743.3 (229040.2-325748.4)	27544.7 (23301.9-33140.7)	514748.1 (423337.5-659216.4)	22673.6 (18647.1-29037.1)	-0.39 (-0.46~-0.32)
El Salvador	37770.8 (32884.2-43303)	23838.8 (20754.6-27330.4)	53128.7 (46251.6-60466.5)	13874.4 (12078.5-15790.6)	-1.8 (-1.93~-1.66)
Equatorial Guinea	4241.9 (3824.3-4618.1)	56530.6 (50966.3-61544.3)	3708.3 (3038.2-4366)	19369.6 (15869.3-22804.8)	-4.13 (-4.53~-3.73)
Eritrea	16511.3 (14631.6-18425.7)	58628.9 (51954.4-65426.6)	33262.3 (28619.5-38083.5)	36232.6 (31175.2-41484.4)	-1.45 (-1.56~-1.33)
Estonia	12579.6 (10114.1-15150.8)	10602 (8524.1-12769)	15651.4 (12281.8-20292.3)	8179.7 (6418.7-10605.1)	-1.12 (-1.27~-0.97)
Eswatini	2555.7	21616.4	3654.5	16254.6	-0.81

	(2175.9-2961.9)	(18404.3-25051.9)	(2943.3-4454.1)	(13091.3-19811.2)	(-0.89~-0.72)
Ethiopia	562118.7	72733.1	810132.9	41090.7	-2.01
	(518304.9-602451.6)	(67064-77951.8)	(724598.4-902935.4)	(36752.3-45797.7)	(-2.23~-1.79)
Fiji	4262.3	31519.6	11147.7	34648.5	0.44
	(3826.8-4783.2)	(28298.5-35371.6)	(9195.7-14085.2)	(28581.5-43778.8)	(0.39~0.5)
Finland	31204.2	6822.7	55342.3	5907.2	-0.37
	(25368.1-37365.2)	(5546.7-8169.8)	(45670.9-67303.2)	(4874.9-7183.9)	(-0.45~-0.29)
France	510837.3	9817.7	1019127.7	10260.6	0.38
	(439105.5-589301.4)	(8439.1-11325.6)	(832130.8-1314484.4)	(8377.9-13234.2)	(0.29~0.48)
Gabon	8731.7	30938.5	9018.2	22256.3	-1.18
	(7465.4-9963.8)	(26451.9-35304.1)	(7342.4-10659.5)	(18120.5-26307.1)	(-1.37~-0.99)
Gambia	8245.1	53439.5	15712.2	35050.2	-1.39
	(7292.2-9174.2)	(47263.3-59461.5)	(14035.7-17668.1)	(31310.3-39413.4)	(-1.42~-1.37)
Georgia	41384	12536.4	47835.7	12824.4	0.06
	(32869.2-50465.8)	(9957-15287.5)	(38020.9-59003.5)	(10193.1-15818.4)	(-0.04~0.16)
Germany	533787.1	6602.7	1003495.2	7402.9	0.79
	(405901.7-642913)	(5020.8-7952.5)	(785216.5-1203102.7)	(5792.6-8875.4)	(0.62~0.97)
Ghana	125697	49603.2	228786.9	33234.2	-1.31
	(111694.6-139591)	(44077.5-55086.1)	(202572.4-259727.1)	(29426.2-37728.7)	(-1.34~-1.27)
Greece	70257.4	7416.4	130687.2	7548.6	0.23
	(57902.9-83229.6)	(6112.3-8785.8)	(104387.6-162241.4)	(6029.5-9371.2)	(0.05~0.4)
Greenland	108.4	8736.3	214.6	7135.6	-0.71
	(84-137.7)	(6764.8-11097.1)	(159.3-277)	(5297.4-9208.8)	(-0.75~-0.68)
Grenada	1076.2	21631.9	923.1	15616	-1.15
	(913.3-1243.9)	(18358.1-25002.7)	(648.3-1317.1)	(10967-22280.5)	(-1.21~-1.09)
Guam	630.2	22635.5	3265.6	26687.7	0.59
	(492-798.7)	(17670-28686.4)	(2598.8-4135.7)	(21238.3-33798.2)	(0.48~0.7)
Guatemala	46616.4	31771.8	92510.5	15693.9	-2.22
	(40948.8-52876)	(27909-36038.1)	(79649.7-106066.3)	(13512.2-17993.6)	(-2.34~-2.1)
Guinea	95216.3	57423.2	94417.2	36381.8	-1.41
	(86706.5-103756.5)	(52291.1-62573.7)	(83048.1-105910.2)	(32000.9-40810.4)	(-1.43~-1.39)
Guinea-Bissau	9149.5	55942.1	9105.7	35870.3	-1.25
	(8153.5-10046.5)	(49852.7-61427)	(8019.9-10316.5)	(31593.1-40640.1)	(-1.31~-1.19)
Guyana	4286.7	24428	5589.4	19317.2	-0.56
	(3658.9-5098.2)	(20850.3-29051.9)	(4411.1-7097.2)	(15245.2-24528.5)	(-0.64~-0.48)
Haiti	47620.3	36009.3	87645.5	30838.1	-0.57
	(41231.1-54481.1)	(31177.9-41197.2)	(74788.6-103463.7)	(26314.4-36403.8)	(-0.62~-0.52)
Honduras	23891.8	24486	49413.2	15679.9	-1.41
	(20517.5-27559.3)	(21027.8-28244.7)	(42662.2-56851.9)	(13537.7-18040.4)	(-1.45~-1.38)
Hungary	140174.1	16534.2	169554.1	12589.6	-0.88
	(117656.1-167589.8)	(13878.1-19768)	(144447.5-198038.7)	(10725.4-14704.6)	(-0.93~-0.83)
Iceland	1235.1	6760	2551.4	6743.8	0.12
	(999.2-1465.9)	(5468.6-8023.1)	(1954.2-3213.8)	(5165.2-8494.7)	(0.01~0.23)
India	11579237.2	64287	33199320.6	55718.6	-0.48

	(11067167.3-12145612.7)	(61444.1-67431.5)	(32033378.7-34286954.5)	(53761.8-57544)	(-0.49~-0.47)
Indonesia	1571924.1	41278.8	3397534.2	35222.5	-0.54
	(1399128.3-1752633)	(36741.1-46024.2)	(2899841.2-4045598.2)	(30062.9-41941.1)	(-0.75~-0.33)
Iran (Islamic Republic of)	237759.7 (192146.8-293897.4)	24503.6 (19802.7-30289.2)	595978.2 (492815.5-742842.9)	16320.7 (13495.7-20342.6)	-1 (-1.15~-0.85)
Iraq	97312.2	24359.9	152175.2	15343.1	-1.56
	(84916.7-110914.1)	(21257-27764.8)	(130185.1-180495.5)	(13125.9-18198.5)	(-1.63~-1.48)
Ireland	18789.1	6992.4	27365.1	5264	-0.77
	(14757.2-23261.3)	(5492-8656.8)	(21673.8-33976.4)	(4169.2-6535.8)	(-0.9~-0.64)
Israel	28058.4	9229.3	75284.6	9118.5	0.2
	(22415-34745.3)	(7373-11428.8)	(61191-91840.2)	(7411.5-11123.8)	(0.12~0.28)
Italy	702937.8	12690.4	1069558	10170.2	-0.65
	(610195.3-806599.6)	(11016.1-14561.9)	(928321.2-1216010.4)	(8827.2-11562.8)	(-0.77~-0.53)
Jamaica	18915.6	16259.6	28469.2	15652.8	-0.09
	(15027.6-24345.3)	(12917.6-20927)	(21734.6-38006.1)	(11950-20896.3)	(-0.13~-0.04)
Japan	951212.5	9630.3	3378394.4	11689.1	0.74
	(745688.8-1192927.7)	(7549.6-12077.5)	(2758744.2-4255405.1)	(9545.1-14723.5)	(0.55~0.93)
Jordan	12693.3	25880.5	58585.5	19076.5	-1.1
	(11178.6-14294.2)	(22792.3-29144.6)	(50811-68290.2)	(16545-22236.5)	(-1.2~-1)
Kazakhstan	144616.4	24103.9	136459.3	16778.5	-1.43
	(124085-166005.3)	(20681.8-27668.9)	(111554.7-164624.9)	(13716.4-20241.7)	(-1.6~-1.26)
Kenya	216273.2	58367.6	348031	37298.4	-1.3
	(192199.8-240974.8)	(51870.7-65034.1)	(307310.5-393776.6)	(32934.4-42201)	(-1.41~-1.19)
Kiribati	561.1	38906.5	855	34360.7	-0.37
	(483.8-643.9)	(33549.6-44649.9)	(724.2-1021.4)	(29103.6-41046.2)	(-0.4~-0.34)
Kuwait	2489.4	12467	10155.9	10286.4	-0.58
	(2114.9-2916.1)	(10591.6-14603.6)	(8485.4-11918.3)	(8594.4-12071.4)	(-0.66~-0.5)
Kyrgyzstan	25644.2	17733.1	29299.8	15265.1	-0.58
	(21615.3-30409.5)	(14947.2-21028.4)	(23125.8-36154.1)	(12048.4-18836.1)	(-0.73~-0.44)
Lao People's Democratic Republic	39552.1 (34841.3-44990.9)	44570.9 (39262.3-50699.9)	57716.5 (49776.7-68883.9)	29962.3 (25840.5-35759.6)	-1.39 (-1.45~-1.32)
Latvia	21770.7	10493	24643.1	8751.7	-0.64
	(17635.7-26352.6)	(8500-12701.4)	(19310-31436.5)	(6857.7-11164.3)	(-0.68~-0.59)
Lebanon	18109.7	18936.3	55246.5	14367	-0.91
	(15855-20658.6)	(16578.7-21601.5)	(48252.6-63522.5)	(12548.2-16519.2)	(-0.95~-0.87)
Lesotho	14823.6	34556.8	10127.4	21064.6	-1.36
	(13392.4-16270.6)	(31220.4-37930.1)	(8502.1-11682.2)	(17684.1-24298.6)	(-1.49~-1.23)
Liberia	25668.3	43141.4	22288.6	27948.3	-1.53
	(22008.3-29462.1)	(36989.9-49517.8)	(18969.9-26220.1)	(23786.9-32878.1)	(-1.61~-1.44)
Libya	15504.2	17840	32268.1	14724.7	-0.53
	(13559.8-17708.9)	(15602.6-20376.8)	(27675.3-37847.5)	(12628.9-17270.7)	(-0.71~-0.35)
Lithuania	27662.8	10899.2	34831.9	8672.4	-0.84
	(22428.9-34457.5)	(8837.1-13576.4)	(26365.6-44702.4)	(6564.5-11130)	(-0.92~-0.76)

Luxembourg	2614.8	7656.8	5160.2	7764.2	0.1
	(2198.9-3093.8)	(6438.9-9059.5)	(4249.2-6151.4)	(6393.5-9255.6)	(0.06~0.15)
Madagascar	113841.6	53030.8	145050.8	39878.6	-0.91
	(101356-126362.8)	(47214.7-58863.6)	(126091.9-163925)	(34666.3-45067.7)	(-1.02~-0.81)
Malawi	99757.2	64140.6	126565.6	40684.6	-1.56
	(89323.9-110045.4)	(57432.3-70755.6)	(111552.1-144679.4)	(35858.5-46507.3)	(-1.67~-1.46)
Malaysia	114959.6	26418.5	305226.9	21584.8	-0.77
	(105793.3-125067.9)	(24312.1-28741.5)	(258074-380276.6)	(18250.3-26892.1)	(-0.82~-0.72)
Maldives	905.5	30182.1	2633.2	19360.5	-1.55
	(765-1127.1)	(25498.9-37568.9)	(1990.9-3518)	(14638.2-25865.7)	(-1.75~-1.35)
Mali	107278.4	66020.2	155162.7	42028.5	-1.49
	(98550.4-116357.9)	(60648.9-71607.8)	(136840.1-175357.8)	(37065.5-47498.7)	(-1.51~-1.46)
Malta	2226.8	8969	5130.3	7146.1	-0.48
	(1818-2688.1)	(7322.5-10826.9)	(4185.1-6269.9)	(5829.5-8733.5)	(-0.59~-0.37)
Marshall Islands	371.1	50547.7	426.6	37155.5	-1
	(325.3-420)	(44308.5-57210.1)	(358.9-518.2)	(31260.4-45135.8)	(-1.09~-0.91)
Mauritania	21406.1	41026.4	27712.8	27223.5	-1.18
	(19026.1-24359.7)	(36464.8-46687.1)	(24226.8-31747.9)	(23799.1-31187.3)	(-1.24~-1.11)
Mauritius	9117	27685.5	26685.1	26610.9	0.04
	(7471.6-11347.8)	(22688.8-34459.6)	(20988.5-34219.6)	(20930.1-34124.4)	(-0.01~0.09)
Mexico	291127.3	14018.2	813138.9	12034.4	-0.24
	(265045.6-320078.6)	(12762.3-15412.2)	(756427.5-874820.6)	(11195.1-12947.3)	(-0.36~-0.11)
Micronesia (Federated States of)	1275.1	54287.9	1223.7	48440.6	-0.33
	(1118.4-1423)	(47613.9-60584.3)	(1064.4-1398.8)	(42134-55371.2)	(-0.39~-0.28)
Monaco	261.2	5272.2	423	5941.5	0.57
	(206.9-323)	(4176-6519.7)	(330.3-553.6)	(4639.7-7776.9)	(0.46~0.69)
Mongolia	9485.8	18459.4	10481.1	12457.2	-1.43
	(8099.9-11041.3)	(15762.5-21486.3)	(8357.6-12786)	(9933.4-15196.7)	(-1.51~-1.36)
Montenegro	5298.9	16557.2	7978.9	13715.2	-1.03
	(4435.4-6342.7)	(13859.2-19818.7)	(6874.4-9399.3)	(11816.8-16156.8)	(-1.29~-0.76)
Morocco	212620.7	29973.5	332539.2	20122.7	-1.2
	(185202.9-240791.1)	(26108.4-33944.8)	(289702-380330.3)	(17530.5-23014.6)	(-1.24~-1.16)
Mozambique	163764.2	67383.8	185583.3	43170.8	-1.5
	(150523.9-176746.4)	(61935.8-72725.6)	(158807-210929.3)	(36942-49066.8)	(-1.55~-1.44)
Myanmar	333930.4	34141.9	598011.1	26760.3	-0.83
	(293038-388265.3)	(29961-39697.2)	(500265.8-719035.1)	(22386.3-32176)	(-0.87~-0.79)
Namibia	5534.7	20843	9273.4	15490.3	-0.92
	(4413.3-6687.1)	(16620-25183)	(7659.4-11359.9)	(12794.2-18975.6)	(-1.01~-0.84)
Nauru	50.9	32264.1	72.8	32208.4	0.03
	(42.4-63.9)	(26873.7-40503.3)	(60.3-93.7)	(26672.6-41440.6)	(-0.07~0.13)
Nepal	169401.1	46459.3	408815.9	35090.8	-0.84
	(154809.6-187749)	(42457.5-51491.4)	(366032.6-463689.2)	(31418.5-39800.9)	(-0.93~-0.76)
Netherlands	102542.5	7993.6	197832	8055.2	0.07

	(84304.5-123530.1)	(6571.8-9629.6)	(157100.9-238860.1)	(6396.7-9725.7)	(-0.07~0.22)
New Zealand	18405.7	7438.7	38517.7	6884.8	-0.19
	(15771.3-21621.2)	(6374-8738.3)	(31286.4-47431.8)	(5592.2-8478.1)	(-0.24~-0.13)
Nicaragua	15690	21318.8	32933.9	13190.3	-1.64
	(13427.5-18084.4)	(18244.6-24572.3)	(28141.5-38419.1)	(11270.9-15387.2)	(-1.81~-1.48)
Niger	78255.4	76120.2	182627	53599.1	-1.12
	(72758.4-83012.6)	(70773.3-80747.7)	(160487.2-203797.7)	(47101.4-59812.5)	(-1.16~-1.08)
Nigeria	603797.7	28818.8	888553.3	23730.8	-0.67
	(508473.3-695522.3)	(24269-33196.7)	(768418.4-1035045)	(20522.3-27643.2)	(-0.74~-0.59)
Niue	45.7	31138.8	36.1	30467.4	-0.07
	(39.4-54.8)	(26847.1-37318)	(29.6-44.7)	(24919-37679.1)	(-0.08~-0.06)
North	25601.1	29049.4	35145.5	18498.3	-1.79
Macedonia	(21630.4-30116.2)	(24543.9-34172.6)	(29376.4-41406.3)	(15461.8-21793.6)	(-1.92~-1.66)
Northern	120.9	25158.7	576.4	27215.2	0.48
Mariana Islands	(96.5-156.5)	(20088.9-32571.9)	(430.8-812.4)	(20341.3-38359.7)	(0.36~0.6)
Norway	32609.2	6674.1	51953.5	7504.7	0.88
	(26333.9-40314.8)	(5389.7-8251.2)	(41636.6-64848.1)	(6014.4-9367.3)	(0.66~1.1)
Oman	6975.8	24171.3	9278.4	13437.9	-1.62
	(5389.6-9219.6)	(18675.3-31946.1)	(7189.7-13428.8)	(10412.9-19449)	(-1.77~-1.48)
Pakistan	1273241.5	45038.2	2039920.4	40285	-0.31
	(1160338.6-1388692.1)	(41044.5-49122)	(1840702.4-2269855.8)	(36350.8-44825.9)	(-0.35~-0.26)
Palau	137	28661.9	271.1	29729.9	0.16
	(114.1-167.1)	(23879.1-34972.5)	(211.7-365.7)	(23218.8-40108.8)	(0.03~0.3)
Palestine	12546	29747.9	17805.3	17151.6	-1.62
	(10881-14264.5)	(25799.9-33822.5)	(15455.8-20705)	(14888.3-19944.8)	(-1.73~-1.52)
Panama	12426.4	15399	29331.1	11239	-0.92
	(10712.1-14375.9)	(13274.6-17814.8)	(25059.2-33868.9)	(9602.1-12977.8)	(-0.99~-0.85)
Papua New	18493.7	30802.9	48638	27948.1	-0.09
Guinea	(16188.6-20884.3)	(26963.5-34784.5)	(41992.8-56874.6)	(24129.7-32681)	(-0.16~-0.02)
Paraguay	19877.8	17142.4	40091.5	13280.2	-0.72
	(16133.5-23694.2)	(13913.3-20433.6)	(32551.2-48488.8)	(10782.5-16061.7)	(-0.77~-0.66)
Peru	112032.1	18686.6	230483.6	12242.4	-1.56
	(91800.7-138401)	(15312.1-23084.9)	(193107.6-278185.8)	(10257.1-14776.1)	(-1.65~-1.47)
Philippines	385103.2	29715.7	824977.4	22805.4	-0.93
	(337253.6-433741.8)	(26023.5-33468.8)	(729483-914889.7)	(20165.6-25291)	(-0.98~-0.88)
Poland	470122.2	19537	615438.7	13176.5	-1.31
	(398888.9-554511.7)	(16576.7-23044)	(530828.2-720131.9)	(11365-15417.9)	(-1.36~-1.27)
Portugal	99664.3	11594.2	174227.5	9845.8	-0.38
	(87589.9-114059.7)	(10189.5-13268.8)	(149803.6-200404.6)	(8465.6-11325.1)	(-0.46~-0.3)
Puerto Rico	24802.9	11096.8	59511.6	11298.8	-0.13
	(18757.4-35421.1)	(8392.1-15847.4)	(45012.5-83937)	(8546-15936.2)	(-0.22~-0.03)
Qatar	505	20924.6	2842.8	16188.1	-0.99
	(405-640.6)	(16781.2-26542.8)	(2274.9-3863.9)	(12954.2-22002.2)	(-1.07~-0.9)

Republic of Korea	143799.5 (102932.8-180948.7)	11328.4 (8108.9-14254.9)	357113 (281256.1-451555.4)	6445.9 (5076.7-8150.5)	-1.91 (-2.17~-1.65)
Republic of Moldova	29102.2 (23918.4-34648.4)	13855.2 (11387.2-16495.6)	39815.8 (31927.5-49108.3)	11425.5 (9161.9-14092.1)	-0.62 (-0.74~-0.5)
Romania	282801.9 (236367.9-337538.6)	19818.5 (16564.5-23654.4)	394229.7 (338986.5-452558.6)	15573.3 (13391-17877.5)	-0.96 (-1.06~-0.85)
Russian Federation	1098589 (888063.9-1372828.1)	11377.5 (9197.2-14217.7)	1530873 (1249666.8-1903047)	10700 (8734.5-13301.3)	-0.37 (-0.47~-0.26)
Rwanda	55520.8 (49033.2-62140.4)	50281.1 (44405.9-56276.1)	82506 (71710.8-94733.9)	33390.8 (29021.9-38339.5)	-1.52 (-1.66~-1.39)
Saint Kitts and Nevis	537.8 (425.3-676)	19895.2 (15735-25009.5)	421.8 (314.6-584.6)	15178.7 (11321.9-21037.1)	-0.79 (-0.82~-0.76)
Saint Lucia	987 (768.5-1284.6)	18883.2 (14702.7-24576)	2224.6 (1671.6-2948.2)	16377.2 (12306.3-21704.4)	-0.31 (-0.4~-0.22)
Saint Vincent and the Grenadines	844.6 (686.9-1048.3)	19356.8 (15742-24026.4)	1303.5 (997.8-1767.8)	16383.5 (12540.7-22219.2)	-0.54 (-0.59~-0.49)
Samoa	1270.9 (1087-1521.2)	33539.8 (28686.5-40144.5)	2181.6 (1788.9-2664.9)	31931.7 (26183.3-39005)	-0.08 (-0.12~-0.04)
San Marino	143.8 (118.5-172.6)	6154.8 (5075.5-7389.8)	395.9 (306.2-497.7)	7598.2 (5876.8-9553.4)	0.93 (0.83~1.03)
Sao Tome and Principe	1336.3 (1163.3-1512.7)	40448.4 (35211.5-45788.3)	1201.3 (1036.7-1379.9)	26204.3 (22614-30100.5)	-1.43 (-1.5~-1.37)
Saudi Arabia	52937.4 (43125.3-69463.9)	21324.6 (17372-27981.9)	91035.3 (73161.1-117233)	17541.4 (14097.3-22589.4)	-0.53 (-0.6~-0.46)
Senegal	84319.5 (77325.9-90937.7)	55757.7 (51133-60134.1)	108166.2 (95418-122905.1)	30549.4 (26948.9-34712.1)	-1.63 (-1.82~-1.44)
Serbia	163574.3 (137561.7-192055.5)	32934.7 (27697.2-38669.2)	221812.2 (192286.9-253630.3)	20515.8 (17785-23458.7)	-1.83 (-1.98~-1.69)
Seychelles	943.4 (800.7-1139.9)	27574.6 (23402.7-33317.2)	1383.5 (1112-1771.6)	25050.8 (20135.2-32079.2)	-0.31 (-0.32~-0.29)
Sierra Leone	55067.8 (48760.1-60401.5)	50973.6 (45134.9-55910.8)	56487.8 (49527.2-64641.9)	32943.8 (28884.4-37699.3)	-1.44 (-1.57~-1.32)
Singapore	10990.6 (8186.5-15126.6)	10500.3 (7821.2-14451.8)	33443.7 (26116.1-42720.1)	6921.5 (5405-8841.3)	-1.26 (-1.35~-1.18)
Slovakia	58621.1 (50265.8-68711.3)	17733 (15205.5-20785.3)	80541.6 (69887.2-93588.8)	13396.6 (11624.4-15566.7)	-0.94 (-0.99~-0.9)
Slovenia	18933.1 (16055.4-22502.1)	13825.5 (11724.1-16431.7)	32499.5 (27048.5-38252.4)	10816 (9001.9-12730.6)	-1.09 (-1.21~-0.97)
Solomon Islands	1878.1 (1608.3-2179)	40006.8 (34259.7-46417.5)	4448.3 (3829-5266.3)	33565.9 (28892.9-39737.9)	-0.46 (-0.53~-0.4)
Somalia	65468.7 (63881.1-66806.7)	93290.3 (91028-95196.9)	166944.5 (158697.1-173330.2)	87626.4 (83297.5-90978.1)	-0.2 (-0.21~-0.19)
South Africa	155064.7	15283.6	266035.5	12024.4	-0.78

	(128967.8-188542)	(12711.5-18583.3)	(232181.3-301682.2)	(10494.2-13635.6)	(-0.81~-0.75)
South Sudan	77700.3	61830.4	68353.2	49104.6	-0.86
	(69741.9-85391)	(55497.5-67950.3)	(60483.3-77498.5)	(43450.9-55674.5)	(-0.91~-0.81)
Spain	275948.6	8006.3	516562	7633.9	0.01
	(227465.6-324995.8)	(6599.6-9429.3)	(422733.4-628962.9)	(6247.3-9295)	(-0.09~0.11)
Sri Lanka	158172.7	32074.5	372447.8	24479	-0.97
	(135071.2-186477.3)	(27389.9-37814.2)	(302593.4-477865.6)	(19887.9-31407.6)	(-0.99~-0.94)
Sudan	186072.6	39481.9	223110.1	26058.8	-1.34
	(159376.9-217916.2)	(33817.4-46238.6)	(191167-262876.5)	(22327.9-30703.4)	(-1.4~-1.28)
Suriname	2617.3	21426.9	5952.2	17889.5	-0.66
	(2068-3216.3)	(16930.5-26331.4)	(4685.2-7567.7)	(14081.4-22744.7)	(-0.72~-0.6)
Sweden	78981.3	7235.8	112432.8	7049.9	0.08
	(65428.5-93463)	(5994.1-8562.5)	(91603.3-142045)	(5743.8-8906.6)	(0.01~0.14)
Switzerland	38732.3	5552.8	86375.6	6923.2	0.95
	(31116-46892.8)	(4460.9-6722.7)	(68072.4-109147.3)	(5456.2-8748.4)	(0.86~1.04)
Syrian Arab	64555.2	28976.3	126795.3	21472.1	-0.88
Republic	(56391.8-76296.7)	(25312.1-34246.6)	(107738.5-153219.5)	(18244.9-25946.9)	(-0.97~-0.79)
Taiwan	122589.1	16809	358324.6	14188.2	-0.45
(Province of China)	(102784.9-147829.1)	(14093.5-20269.8)	(307727.3-431273.7)	(12184.8-17076.7)	(-0.62~-0.29)
Tajikistan	29738.1	23220	45770.5	23006.2	-0.06
	(25396-34468.1)	(19829.6-26913.3)	(38507.3-54653.2)	(19355.4-27471.1)	(-0.21~0.1)
Thailand	479209.1	31349.9	1542932.5	24765.6	-0.67
	(412776.3-555544.1)	(27003.9-36343.7)	(1171502-2105568.4)	(18803.8-33796.5)	(-0.72~-0.62)
Timor-Leste	3103.2	36369.5	12271.6	27137.7	-1.02
	(2675.8-3605.6)	(31360.2-42257.6)	(10209.6-15310.5)	(22577.7-33858)	(-1.13~-0.91)
Togo	22820.7	45014.4	47138.8	32615.6	-0.94
	(19987.8-25884.9)	(39426.4-51058.7)	(40280-53882.5)	(27869.9-37281.5)	(-0.99~-0.88)
Tokelau	24.1	31995.6	27.7	30282.8	-0.11
	(20.8-29)	(27641-38530.5)	(23-34.7)	(25107.9-37916)	(-0.14~-0.09)
Tonga	812.3	31276.4	1341.7	30624.5	0
	(687.2-965.9)	(26458-37189.3)	(1121.2-1662.3)	(25591.6-37943.4)	(-0.02~0.02)
Trinidad and	7732	16378.7	15090	13486	-0.88
Tobago	(5777.7-10407.6)	(12239-22046.4)	(10894.5-21875.8)	(9736.5-19550.5)	(-0.98~-0.77)
Tunisia	48791.8	20182.6	112512.2	16159.7	-0.62
	(42285.8-55992.5)	(17491.4-23161.1)	(95205.8-130750.7)	(13674-18779.2)	(-0.66~-0.57)
Türkiye	416200.8	27746.4	881864.9	17346.2	-1.49
	(364886.1-468945.6)	(24325.4-31262.6)	(781568.9-1005894.9)	(15373.4-19785.9)	(-1.51~-1.47)
Turkmenistan	12908.6	15246.7	21247.8	13564.2	-0.41
	(10898.5-15363)	(12872.5-18145.7)	(16759.5-26819.3)	(10699-17120.9)	(-0.49~-0.32)
Tuvalu	95.9	35542.7	162.1	31808.5	-0.24
	(82.5-112.3)	(30554.3-41593.6)	(134.4-199.5)	(26374.6-39164.9)	(-0.28~-0.19)
Uganda	151870.2	51889	162403.3	26990.1	-2.24
	(133772.5-168652.5)	(45705.6-57622.9)	(138975.6-187780.1)	(23096.6-31207.5)	(-2.32~-2.17)

Ukraine	429187.4 (350927.6-530663.3)	10707.2 (8754.8-13238.8)	512196.5 (421851.2-619235.9)	10503 (8650.4-12698)	-0.15 (-0.25~-0.04)
United Arab Emirates	2787.3 (2248.2-3748.6)	22904.4 (18473.9-30803.3)	9135.8 (7337.8-11782.3)	18923.5 (15199.2-24405.5)	-0.39 (-0.54~-0.24)
United Kingdom	362678 (299904.7-448521.6)	5828.5 (4819.7-7208)	536336.8 (423628.2-705460.5)	5862.3 (4630.3-7710.8)	0.26 (0.15~0.37)
United Republic of Tanzania	219393.4 (187140-249136.7)	45032.9 (38412.6-51138.1)	349102.1 (305627-404258.7)	31431.3 (27517-36397.3)	-1.19 (-1.3~-1.08)
United States of America	1358061.4 (1172735.4-1524882.7)	6403.3 (5529.4-7189.8)	2740182.6 (2276556.3-3228648.9)	7120 (5915.3-8389.2)	0.61 (0.49~0.72)
United States Virgin Islands	455.1 (337.7-646)	11358 (8428.6-16123.9)	1325.5 (946.3-1913.3)	10278.2 (7338.1-14836.9)	-0.49 (-0.57~-0.41)
Uruguay	29854.3 (24393.8-35943.1)	12162.4 (9937.8-14642.9)	41626.5 (35424.2-48863.5)	11077.7 (9427.1-13003.6)	-0.24 (-0.3~-0.19)
Uzbekistan	114232.4 (98835.8-130772.1)	20028.5 (17329-22928.4)	161821 (133365.8-198035.2)	16971.2 (13986.9-20769.2)	-0.79 (-0.89~-0.69)
Vanuatu	845.8 (713.9-1005.4)	36748.8 (31016.3-43684.6)	2296.7 (1963.2-2677.8)	34498.8 (29489.9-40222.8)	-0.21 (-0.24~-0.17)
Venezuela (Bolivarian Republic of)	91533.7 (79653.2-104446.8)	19281.8 (16779.1-22002)	247742 (211981.9-287682.9)	15426.7 (13199.9-17913.7)	-0.79 (-0.9~-0.67)
Viet Nam	545806.7 (479675.4-623298)	26768.1 (23524.8-30568.5)	948789.5 (818261.3-1107274)	21236.6 (18315.1-24784)	-0.77 (-0.82~-0.73)
Yemen	98541.3 (87587-110592.1)	50512.7 (44897.5-56690)	199358.8 (179833.4-223992.8)	33514.1 (30231.7-37655.3)	-1.38 (-1.49~-1.28)
Zambia	59870.7 (52693.9-67460.1)	51685.6 (45490-58237.5)	93261.6 (76454.1-112975.7)	34658.9 (28412.7-41985.3)	-1.42 (-1.57~-1.28)
Zimbabwe	50225.2 (43782.3-57931.2)	28006.1 (24413.5-32303)	70788.5 (60640.6-82491.3)	26666.6 (22843.8-31075.1)	0.33 (0.14~0.51)

eTable 5. The incidence cases and incidence of malnutrition among individuals over 70 in 1990 and 2021, and its temporal trends from 1990 to 2021, at the national level

Abbreviations: EAPC, estimated annual percentage change.

Location	1990		2021		1990-2021
	Case number (95% UI)	Rate (95%UI)	Case number (95% UI)	Rate (95% UI)	EAPC % (95% CI)
Afghanistan	130640.3 (108457.9-154777)	42037.2 (34899.4-49803.9)	71558.4 (55458.1-90522.5)	17688.7 (13708.8-22376.5)	-2.87 (-3.29~-2.45)
Albania	14562.3 (11220.5-18842.2)	14587.8 (11240.2-18875.3)	25763.7 (19882.8-32521.9)	9500.2 (7331.7-11992.3)	-1.94 (-2.28~-1.59)
Algeria	51255.9 (38638.6-69032.7)	8230 (6204.1-11084.4)	59253.2 (47830.1-74350.1)	3424.7 (2764.4-4297.2)	-2.67 (-2.74~-2.6)
American Samoa	39.9 (32.8-49.2)	4549.5 (3732.8-5598.8)	66.7 (58.2-76.5)	3012.3 (2629.3-3453.7)	-1.4 (-1.46~-1.33)
Andorra	51.5 (40.2-65.8)	1607 (1254.5-2053.1)	139.5 (94.6-210.5)	1494.6 (1013.4-2255.6)	0.28 (-0.07~0.62)
Angola	35802.5 (27468.8-44867.3)	27916.9 (21418.7-34985.1)	42187.4 (31808.8-55463.7)	10513 (7926.7-13821.4)	-3.28 (-3.49~-3.06)
Antigua and Barbuda	199.1 (172.7-228.3)	5472.3 (4748.4-6276.6)	291.6 (258.6-337.7)	5191.1 (4603.5-6012)	-0.2 (-0.39~0)
Argentina	132434.6 (101957.1-168634.2)	7161.5 (5513.4-9119)	229537.5 (184300.8-287610.8)	6441 (5171.6-8070.6)	-0.48 (-0.89~-0.07)
Armenia	1915.6 (1568.4-2333.5)	1603.5 (1312.9-1953.3)	2581.8 (2087.6-3197.4)	1056.1 (853.9-1307.9)	-1.67 (-1.83~-1.51)
Australia	18947.9 (15865.2-22647.9)	1566 (1311.2-1871.8)	53286.8 (45919.6-62532.4)	1727.9 (1489-2027.7)	0.43 (0.25~0.6)
Austria	14533.2 (11092.3-19012)	1906.3 (1454.9-2493.7)	13936.1 (9881.9-21238.6)	1120.3 (794.4-1707.4)	-0.83 (-1.33~-0.33)
Azerbaijan	7052.2 (5347.4-9289.2)	3139.3 (2380.4-4135.1)	10050.1 (7922.7-12976.2)	2622.8 (2067.6-3386.4)	-0.95 (-1.46~-0.43)
Bahamas	199.1 (166.4-234.6)	2463.2 (2058.2-2901.7)	336.8 (285.5-405.1)	1737.8 (1473-2090.2)	-1.25 (-1.39~-1.11)
Bahrain	346.7 (282.2-444.3)	6060.2 (4933.2-7766.7)	983.2 (828.7-1153.8)	3902.6 (3289.6-4580)	-1.23 (-1.38~-1.08)
Bangladesh	374448.2 (294117.7-469755.1)	16457 (12926.5-20645.7)	348917.5 (294670.9-422933.9)	4734.5 (3998.4-5738.9)	-3.98 (-4.08~-3.89)
Barbados	678.3 (576.2-823.1)	3174.1 (2696.1-3851.5)	899.7 (769.6-1053.3)	2807.5 (2401.6-3287)	-0.48 (-0.65~-0.31)
Belarus	14965.3 (11498.4-20080.3)	2151.1 (1652.8-2886.3)	11898.9 (9415.7-15043.5)	1247 (986.8-1576.6)	-2.07 (-2.25~-1.9)
Belgium	21556.3 (17824.6-25843)	2243.3 (1855-2689.4)	58776.1 (45456.4-89089.5)	3655.5 (2827.1-5540.8)	1.97 (1.72~2.22)
Belize	412.1	7822.8	560.8	4016.9	-2.08

	(324.1-519.8)	(6151.8-9866.6)	(443.9-700.8)	(3179.8-5019.2)	(-2.21~-1.96)
Benin	40128.2	39118	35410.3	15821.8	-2.76
	(33663.3-46853.6)	(32815.8-45674.1)	(27096.1-45534.7)	(12106.9-20345.6)	(-2.84~-2.68)
Bermuda	86.3	2482.6	190.1	2109.8	-0.81
	(71.8-105.6)	(2066.9-3040.1)	(163.7-222.7)	(1816.7-2471.5)	(-0.97~-0.65)
Bhutan	1900.2	21192.5	1642.4	5094.6	-4.69
	(1452.5-2412.5)	(16198.9-26905.5)	(1305.7-2030.5)	(4050.3-6298.4)	(-4.92~-4.46)
Bolivia	4753	3166.4	15160.2	3276.1	-0.41
(Plurinational State of)	(3793.1-6077.1)	(2526.9-4048.5)	(12169.4-19492.5)	(2629.8-4212.3)	(-0.9~0.07)
Bosnia and	25854.3	15420.6	34554.4	8892.3	-2.5
Herzegovina	(19481.5-33922.1)	(11619.6-20232.6)	(26138.6-45297.5)	(6726.5-11656.9)	(-2.74~-2.26)
Botswana	2827	12307.5	2038.9	3482.2	-3.82
	(2131.2-3657.2)	(9278.1-15922.1)	(1573.2-2583.2)	(2686.8-4411.8)	(-4.02~-3.61)
Brazil	841113.7	19789.4	1350984.5	9607.7	-2.24
	(655111.1-1046194)	(15413.2-24614.5)	(1092736.4-1699913.5)	(7771.2-12089.2)	(-2.33~-2.14)
Brunei	120.8	2713.1	254.9	1868.2	-1.25
Darussalam	(100.2-144.7)	(2250.6-3248.4)	(213.6-294.5)	(1565.6-2158)	(-1.47~-1.02)
Bulgaria	66738.1	10119.4	81362.8	7903.8	-0.67
	(49345.7-87432)	(7482.2-13257.2)	(63153.3-104350.6)	(6134.9-10136.9)	(-0.97~-0.37)
Burkina	108435.6	55233.8	86626	20566.5	-3.23
Faso	(95651.4-121864.8)	(48722-62074.2)	(65527.4-109553.4)	(15557.4-26009.9)	(-3.31~-3.16)
Burundi	50734.4	44839.5	47261.3	26235.3	-1.92
	(41782.3-60052.6)	(36927.6-53075)	(36309.6-59121.8)	(20155.9-32819.3)	(-2.06~-1.78)
Cabo	3111.5	20809.1	916.2	4473.1	-4.98
Verde	(2510.8-3833.7)	(16792-25639.4)	(737-1140.1)	(3598-5565.9)	(-5.19~-4.77)
Cambodia	19785	10609.2	14799.3	2705.3	-4.55
	(16047.2-24352)	(8605-13058.2)	(12454.9-17665.3)	(2276.7-3229.1)	(-4.95~-4.15)
Cameroon	104314	56661.5	81818.3	16354	-3.72
	(94700.7-114434.4)	(51439.7-62158.7)	(60388.7-107019.4)	(12070.6-21391.3)	(-3.97~-3.46)
Canada	46659.7	2319.9	143206.4	2971.8	0.88
	(39168.8-55903.6)	(1947.4-2779.4)	(89733.6-206108.2)	(1862.2-4277.2)	(0.55~1.21)
Central	11884.4	31986.5	12905.4	20226.7	-1.46
African Republic	(9334.3-14562.1)	(25123-39193.4)	(9763.8-16422.1)	(15302.8-25738.4)	(-1.52~-1.4)
Chad	89616.2	59502	69920.5	29338.9	-2.26
	(78929-99409.7)	(52406.1-66004.5)	(54956.4-86962.6)	(23059.9-36489.8)	(-2.35~-2.16)
Chile	15485.6	2866.1	40701.4	2613	-0.35
	(12468.9-18954.2)	(2307.8-3508.1)	(30297.3-54254)	(1945.1-3483.1)	(-0.64~-0.06)
China	1186962.6	3162.7	3232243.2	2709.2	0.24
	(958209.2-1489617.7)	(2553.2-3969.2)	(2668102.2-3925403.8)	(2236.4-3290.3)	(-0.08~0.56)
Colombia	61206	7317	99042.5	3107.9	-2.95
	(46681.6-78366.1)	(5580.6-9368.4)	(80238.9-122991.9)	(2517.9-3859.5)	(-3.09~-2.8)
Comoros	3285	40544.1	3526.7	15543.1	-3.07

	(2721-3927.8)	(33583.1-48478)	(2724.6-4529.5)	(12008-19963.1)	(-3.11~-3.03)
Congo	11559.6	26511.4	19741.1	20526.7	-0.47
	(9095.7-14476.7)	(20860.5-33201.8)	(15230.3-25132.9)	(15836.4-26133.1)	(-0.86~-0.07)
Cook Islands	26.5 (20.1-33.5)	4508.9 (3416-5692.6)	29.8 (25.1-35.4)	1911.5 (1611.3-2271.2)	-2.65 (-2.83~-2.47)
Costa Rica	5365.9 (4114.9-6923.8)	5664.8 (4344.2-7309.6)	12727 (10221.4-15594.8)	4058.3 (3259.4-4972.8)	-0.89 (-0.99~-0.78)
Côte d’Ivoire	27726.6	20721.8	35805.8	8587.3	-2.47
	(21238.5-35303.4)	(15872.8-26384.3)	(26782.6-47206.8)	(6423.3-11321.6)	(-2.59~-2.35)
Croatia	25334.9	8938.3	35312.2	5743.4	-1.87
	(18453.9-34623.3)	(6510.6-12215.2)	(26485.7-46976.9)	(4307.8-7640.7)	(-2.17~-1.57)
Cuba	21843.7	3490.8	31509.2	2537.5	-1.48
	(16635.3-27905)	(2658.4-4459.4)	(25394.3-40554)	(2045.1-3265.9)	(-1.77~-1.19)
Cyprus	1537.6	2981.5	3005.3	2219.9	-0.12
	(1226.5-1933.9)	(2378.3-3750)	(2598.8-3483)	(1919.6-2572.7)	(-0.47~0.24)
Czechia	65140.1	8173.2	93422.3	6049.1	-0.57
	(47758.3-89500.4)	(5992.3-11229.8)	(76194.9-112680.4)	(4933.7-7296.1)	(-0.76~-0.37)
Democratic People's Republic of Korea	21394.6 (17423.8-25833.7)	3267.6 (2661.1-3945.5)	37998.1 (32365.2-45125.4)	2171.2 (1849.4-2578.5)	-6.44 (-8.06~-4.8)
Democratic Republic of the Congo	138592.8 (101865.3-174778)	24491.9 (18001.5-30886.5)	212693.8 (163243.8-269327.4)	16821.1 (12910.3-21300.1)	-1.1 (-1.7~-0.49)
Denmark	8415.5	1505.5	17010.9	1978.7	1.25
	(6477.8-10676)	(1158.8-1909.9)	(14557.7-23357.7)	(1693.4-2717)	(0.75~1.75)
Djibouti	1421.3	33440.1	3338.5	15243.6	-2.61
	(1152.3-1738.5)	(27111.2-40904.5)	(2523.3-4358.3)	(11521.4-19900.3)	(-2.75~-2.47)
Dominica	160	4148.1	101.5	2249.3	-2.07
	(129-196.1)	(3344.2-5084)	(84-124.1)	(1861.2-2750.6)	(-2.16~-1.97)
Dominican Republic	21902.6 (17211.9-28267.5)	11878.9 (9334.9-15330.8)	25009.6 (19608.2-31516.6)	4506.9 (3533.5-5679.5)	-3.31 (-3.42~-3.2)
Ecuador	5436.9	2007.6	14028.7	1500.6	-0.86
	(4752.3-6241.6)	(1754.9-2304.8)	(12299.5-15959.9)	(1315.6-1707.1)	(-1.07~-0.64)
Egypt	68981.9	7018	63801.3	2810.3	-2.12
	(52519.9-91201.6)	(5343.2-9278.6)	(48788.4-82424.4)	(2149-3630.6)	(-2.43~-1.81)
El Salvador	22585.3	14254.5	21385.6	5584.8	-3.14
	(17705.1-28624.3)	(11174.5-18066)	(17133.1-26483.3)	(4474.3-6916)	(-3.27~-3.01)
Equatorial Guinea	1969.7 (1526.9-2440.8)	26249.5 (20348.7-32528.6)	673.7 (520.1-871)	3518.7 (2716.6-4549.5)	-6.9 (-7.22~-6.57)
Eritrea	13828.7	49103.6	18073.6	19687.6	-2.77
	(11463.8-16285.3)	(40705.9-57826.3)	(14154-22506.3)	(15417.9-24516.1)	(-2.95~-2.58)
Estonia	2227.7	1877.5	1917	1001.8	-2.11
	(1744.8-2851.6)	(1470.5-2403.3)	(1538.5-2339)	(804-1222.4)	(-2.3~-1.93)

Eswatini	777.9	6579.8	621.1	2762.7	-2.53
	(572.8-1040.3)	(4844.7-8799.2)	(464.4-837.9)	(2065.5-3727)	(-2.63~-2.43)
Ethiopia	467075.2	60435.3	427556.2	21686	-3.68
	(400685.7-530673.9)	(51845.1-68664.4)	(332548.2-535445.5)	(16867.1-27158.3)	(-4.02~-3.33)
Fiji	932.2	6893.2	1160.6	3607.4	-2.03
	(743-1174.8)	(5494.8-8687.9)	(984.6-1364.6)	(3060.2-4241.4)	(-2.08~-1.97)
Finland	6977.2	1525.6	11398	1216.6	-0.12
	(5485.9-8791.2)	(1199.5-1922.2)	(8897.3-14597.6)	(949.7-1558.1)	(-0.4~0.17)
France	168568	3239.7	438000.9	4409.8	1.3
	(146344.8-194072.8)	(2812.6-3729.8)	(353587.5-662202.6)	(3559.9-6667)	(1.12~1.47)
Gabon	2456.6	8704.4	1612.3	3979.1	-2.26
	(1829.3-3289.5)	(6481.7-11655.6)	(1227-2100.1)	(3028.1-5182.8)	(-2.46~-2.05)
Gambia	5935.8	38472.1	7125	15894.1	-2.76
	(4818-7099.2)	(31227.1-46012.5)	(5493.8-9017.6)	(12255.3-20116.2)	(-2.8~-2.73)
Georgia	7118.1	2156.3	7728.3	2071.9	-0.15
	(5414.6-9284)	(1640.2-2812.4)	(6237.5-9741.1)	(1672.2-2611.5)	(-0.63~0.32)
Germany	81987.6	1014.1	366262.5	2702	4.14
	(62664.2-103893)	(775.1-1285.1)	(230748.9-459398)	(1702.3-3389)	(3.78~4.52)
Ghana	84996.9	33541.9	82717.3	12015.8	-3.31
	(69204-100350.5)	(27309.7-39600.9)	(62775-106957.1)	(9118.9-15536.9)	(-3.43~-3.2)
Greece	19272.3	2034.4	22807.1	1317.4	-0.32
	(15346.4-23771.5)	(1620-2509.3)	(17574.3-29988.2)	(1015.1-1732.1)	(-0.74~0.1)
Greenland	17.2	1389.5	41	1362.1	-0.16
	(14.2-20.9)	(1140.8-1684.4)	(33.3-60.1)	(1105.8-1998.4)	(-0.24~-0.07)
Grenada	345.4	6943.5	201.3	3404.7	-2.44
	(272.8-429.2)	(5483.2-8627.8)	(164.5-251.6)	(2783.4-4256.7)	(-2.51~-2.38)
Guam	97.1	3486.2	273.1	2231.8	-1.55
	(81-119.3)	(2909.3-4286.3)	(238.9-318)	(1952-2598.8)	(-1.67~-1.44)
Guatemala	34038.9	23199.5	36443.1	6182.4	-4.31
	(28169.1-41601.3)	(19198.9-28353.7)	(29568.6-44978)	(5016.2-7630.3)	(-4.4~-4.22)
Guinea	71023.5	42833	41098.9	15836.6	-2.99
	(60937.6-82151.7)	(36750.4-49544.2)	(30717.7-52750.8)	(11836.5-20326.5)	(-3.06~-2.92)
Guinea-Bissau	6649	40653.5	4056.2	15978.7	-2.72
	(5425.2-7733.8)	(33171-47286.4)	(3025.3-5268.6)	(11917.6-20754.6)	(-2.81~-2.63)
Guyana	1377.2	7847.7	1165.5	4028.1	-2.34
	(1103.4-1729.2)	(6287.8-9853.8)	(957.4-1405.8)	(3308.8-4858.7)	(-2.46~-2.21)
Haiti	22520.3	17029.3	33636.6	11835.1	-1.42
	(17093.4-29041)	(12925.6-21960.1)	(25573.6-43212.8)	(8998.1-15204.5)	(-1.6~-1.23)
Honduras	13744.8	14086.7	18200.4	5775.4	-2.95
	(10610.4-17430)	(10874.3-17863.6)	(14067.2-23607)	(4463.8-7491)	(-3~-2.91)
Hungary	78485.1	9257.7	81865.9	6078.6	-1.47
	(56398.2-104846.3)	(6652.4-12367.1)	(63003.2-108555.6)	(4678.1-8060.4)	(-1.61~-1.33)
Iceland	373.4	2043.5	804.8	2127.2	0.39
	(308.8-443.4)	(1690.2-2426.9)	(639.9-1212)	(1691.4-3203.4)	(0.21~0.57)

India	4328373.9 (3401429.7-5514583)	24030.8 (18884.5-30616.5)	4896343.1 (3819392.4-6177789.6)	8217.6 (6410.1-10368.2)	-3.69 (-3.89~-3.49)
Indonesia	412806.3 (317491-539209.2)	10840.3 (8337.3-14159.6)	486414.8 (420718.1-564795.4)	5042.7 (4361.6-5855.3)	-2.33 (-2.82~-1.84)
Iran (Islamic Republic of)	74140.7 (53491.1-108022.7)	7641 (5512.8-11132.8)	93547.9 (74466.2-114005.1)	2561.8 (2039.2-3122)	-3.02 (-3.43~-2.6)
Iraq	43154.2 (33034.7-55864.2)	10802.7 (8269.5-13984.3)	31706.9 (24638.9-41677.8)	3196.9 (2484.2-4202.2)	-3.76 (-4.05~-3.48)
Ireland	4773.7 (3875.6-5997.4)	1776.6 (1442.3-2232)	8123.1 (5697.9-11938.2)	1562.6 (1096.1-2296.5)	0.11 (-0.08~0.29)
Israel	6603.4 (5326.6-8052.2)	2172.1 (1752.1-2648.6)	21653.9 (16854-30722.9)	2622.7 (2041.4-3721.2)	1.29 (1.06~1.52)
Italy	300496.3 (243741.8-366130.9)	5425 (4400.4-6609.9)	412053.1 (335579.5-504683.6)	3918.1 (3191-4798.9)	-0.53 (-0.71~-0.35)
Jamaica	5303.4 (4343.7-6443.8)	4558.7 (3733.8-5539.1)	4975.7 (4130.7-6033.8)	2735.7 (2271.1-3317.5)	-1.88 (-2.01~-1.74)
Japan	148470.6 (115032.5-190453.1)	1503.2 (1164.6-1928.2)	469114.1 (390887.5-571611.9)	1623.1 (1352.5-1977.7)	0.07 (-0.16~0.3)
Jordan	5275.8 (4084.8-6873.2)	10757 (8328.5-14013.8)	15518.2 (12036.7-19838.5)	5053 (3919.4-6459.8)	-2.46 (-2.77~-2.14)
Kazakhstan	67350.9 (53872.6-86098.4)	11225.7 (8979.2-14350.4)	56056.4 (42447.3-72803.8)	6892.5 (5219.2-8951.7)	-1.49 (-1.6~-1.39)
Kenya	183106.8 (153107.6-214457)	49416.7 (41320.6-57877.5)	210579.8 (159670.7-263957.9)	22567.8 (17111.9-28288.3)	-2.34 (-2.51~-2.17)
Kiribati	191.2 (146-249.7)	13259.7 (10123.4-17316.8)	151.7 (123.7-187.5)	6098.3 (4971.6-7535.3)	-2.5 (-2.63~-2.36)
Kuwait	534.6 (413.7-710.9)	2677.3 (2071.8-3560)	1004.2 (799.4-1243.5)	1017.1 (809.7-1259.5)	-3.08 (-3.17~-2.98)
Kyrgyzstan	6269.7 (4653.7-8285.1)	4335.5 (3218.1-5729.2)	7487.2 (5633-10093.3)	3900.8 (2934.8-5258.6)	-0.02 (-0.35~0.3)
Lao People's Democratic Republic	20203.2 (15686.6-25086)	22766.9 (17677.1-28269.2)	11623.6 (9074.6-15188.4)	6034.2 (4710.9-7884.7)	-4.59 (-4.77~-4.41)
Latvia	3312.9 (2496.6-4354.9)	1596.7 (1203.3-2099)	2744.5 (2187-3420.4)	974.7 (776.7-1214.7)	-1.65 (-1.8~-1.49)
Lebanon	5377.1 (4083.6-6984.7)	5622.5 (4270-7303.5)	10217.6 (8454.6-12481.7)	2657.1 (2198.6-3245.9)	-2.45 (-2.6~-2.3)
Lesotho	4398.3 (3369.9-5573.8)	10253.4 (7856-12993.7)	2128.3 (1588.1-2875.5)	4426.7 (3303.1-5981)	-2.41 (-2.52~-2.29)
Liberia	17888.3 (13801.6-22128.1)	30065.4 (23196.7-37191.4)	9631.4 (7049-13123)	12077.1 (8838.9-16455.3)	-3.14 (-3.32~-2.96)
Libya	5040.4 (3960.5-6375.1)	5799.8 (4557.1-7335.6)	5067.4 (4033.3-6174.9)	2312.4 (1840.5-2817.8)	-2.49 (-2.75~-2.23)
Lithuania	4270.1	1682.4	3353.6	835	-2.43

	(3220.4-5791.5)	(1268.9-2281.9)	(2666.4-4157.8)	(663.9-1035.2)	(-2.57~-2.29)
Luxembourg	764.9	2239.7	1720.2	2588.3	0.73
	(649.9-916.6)	(1903.1-2684)	(1466.1-2056.9)	(2205.9-3094.9)	(0.57~0.9)
Madagascar	80185	37352.6	74531.6	20490.9	-1.95
	(65151.4-97617.7)	(30349.5-45473.2)	(57019.3-96297)	(15676.3-26474.8)	(-2.19~-1.71)
Malawi	82563.1	53085.4	72362.5	23261	-2.8
	(70376.3-94775.8)	(45249.7-60937.7)	(55496.9-91753.5)	(17839.5-29494.2)	(-3~-2.6)
Malaysia	13363.4	3071	37167.4	2628.4	-0.62
	(11306.1-15969.9)	(2598.2-3670)	(32025-43352.5)	(2264.7-3065.8)	(-0.71~-0.53)
Maldives	285	9500.3	358.5	2636	-3.71
	(224.2-357.7)	(7474.1-11921.6)	(310.7-422.6)	(2284.2-3107.2)	(-4.27~-3.14)
Mali	91221.7	56138.7	86744.6	23496.3	-2.77
	(80129.4-101773.2)	(49312.4-62632.3)	(67228.7-107354.7)	(18210.1-29078.9)	(-2.85~-2.69)
Malta	620.5	2499.2	1221.3	1701.2	-0.68
	(486.6-767.6)	(1960-3091.8)	(1001.5-1536.7)	(1394.9-2140.5)	(-0.88~-0.48)
Marshall Islands	231.7	31565.3	124.2	10816.1	-3.54
	(183.5-290.5)	(24996.3-39575.5)	(91.7-169.3)	(7986.7-14749.2)	(-3.85~-3.24)
Mauritania	10814.4	20726.5	8445.5	8296.4	-2.61
	(8532.5-13448.7)	(16353.1-25775.4)	(6695.7-10643.7)	(6577.4-10455.8)	(-2.73~-2.5)
Mauritius	1583.4	4808.1	2581.8	2574.6	-1.39
	(1368.9-1841.5)	(4156.8-5592)	(2214.1-3056.3)	(2207.9-3047.8)	(-1.64~-1.15)
Mexico	130630.3	6290	297526.8	4403.4	-0.68
	(106102.7-161261.6)	(5109-7765)	(254482.6-348642.8)	(3766.3-5159.9)	(-0.99~-0.38)
Micronesia (Federated States of)	857.6	36510	671.9	26596.7	-0.96
	(694.5-1021.4)	(29569.3-43484.8)	(524.6-842.4)	(20768.4-33349.2)	(-1.06~-0.86)
Monaco	75.6	1526.7	122.6	1722.3	0.84
	(56.1-114.5)	(1132.8-2311.5)	(83.7-184.3)	(1176.4-2589.1)	(0.57~1.12)
Mongolia	2257.6	4393.2	1487.2	1767.6	-2.83
	(1685.5-3071.6)	(3279.9-5977.3)	(1110.5-2016.6)	(1319.8-2396.8)	(-2.97~-2.69)
Montenegro	2617.3	8178.1	3308.8	5687.7	-1.85
	(1956.9-3518.1)	(6114.7-10992.8)	(2452.4-4335.4)	(4215.5-7452.2)	(-2.32~-1.38)
Morocco	107441.3	15146.2	91437.7	5533.1	-3.05
	(80201.7-136338.7)	(11306.2-19219.9)	(71568.7-114440.3)	(4330.8-6925)	(-3.23~-2.87)
Mozambique	140827.5	57946.1	110148.6	25623	-2.73
	(124111.5-158190.9)	(51068-65090.6)	(85054.4-138892.3)	(19785.5-32309.4)	(-2.86~-2.59)
Myanmar	80484.9	8229	46506.8	2081.1	-4.65
	(61479-104447.5)	(6285.8-10679)	(39920.2-54364.9)	(1786.4-2432.8)	(-5.01~-4.29)
Namibia	1661.7	6257.9	1835.2	3065.5	-2.18
	(1292.3-2165)	(4866.8-8153.1)	(1506.6-2320.9)	(2516.6-3876.9)	(-2.28~-2.07)
Nauru	7.9	4978.8	8.2	3618.6	-1.14
	(5.9-10.7)	(3753.1-6776.3)	(6.7-10.5)	(2949.7-4634.5)	(-1.37~-0.9)
Nepal	81775.7	22427.5	75405.2	6472.4	-4.07
	(64023.9-103307.8)	(17559-28332.8)	(60385.3-93678)	(5183.2-8040.9)	(-4.25~-3.9)

Netherlands	32424.1 (24694.8-48652.7)	2527.6 (1925-3792.7)	74409.1 (48458.6-103870.8)	3029.7 (1973.1-4229.3)	0.69 (0.25~1.13)
New Zealand	3272.2 (2551.6-4136.3)	1322.5 (1031.2-1671.7)	6576.1 (5112.6-8973.8)	1175.4 (913.8-1604)	-0.12 (-0.25~0.01)
Nicaragua	8480.7 (6612.9-10826.9)	11523.1 (8985.3-14711.1)	8425.8 (6728.6-10392.7)	3374.6 (2694.9-4162.4)	-4.32 (-4.68~-3.96)
Niger	71455.3 (65215.3-77341.9)	69505.7 (63436-75231.7)	135224.2 (111064.9-159945.8)	39686.9 (32596.4-46942.4)	-1.75 (-1.83~-1.68)
Nigeria	257370.7 (195565.2-343314.1)	12284.1 (9334.2-16386.1)	214433.8 (157178.4-283467.4)	5726.9 (4197.8-7570.6)	-2.6 (-2.91~-2.29)
Niue	7 (5.8-8.7)	4794.8 (3974.8-5896.5)	3.4 (3-3.9)	2868.3 (2501.7-3306.5)	-1.66 (-1.77~-1.54)
North Macedonia	17689.7 (13797-22370.4)	20072.3 (15655.4-25383.6)	20499 (15324.3-27115.4)	10789.4 (8065.7-14271.8)	-2.48 (-2.73~-2.24)
Northern Mariana Islands	16.7 (14.1-19.9)	3479.5 (2934.9-4130.9)	67.1 (58.4-77.1)	3167.7 (2758.5-3641)	-0.25 (-0.31~-0.19)
Norway	6804.4 (5314.7-8405.2)	1392.6 (1087.8-1720.3)	18744.2 (14475.2-24791.7)	2707.6 (2090.9-3581.2)	3.47 (3.03~3.9)
Oman	2994.3 (2263.5-3943)	10375.4 (7843-13662.5)	1777.2 (1459.6-2119.1)	2574 (2113.9-3069.2)	-4.04 (-4.42~-3.66)
Pakistan	349963.2 (259152.8-466476.2)	12379.2 (9167-16500.6)	171718.2 (134440.6-218723.1)	3391.1 (2655-4319.4)	-3.78 (-4.08~-3.48)
Palau	17.1 (14-21.4)	3575.4 (2925.7-4479.7)	23 (19.8-26.9)	2525.3 (2171-2948.3)	-0.97 (-1.06~-0.88)
Palestine	7174.5 (5391.4-9163.8)	17011.4 (12783.5-21728.4)	4438.9 (3453.2-5742.8)	4275.9 (3326.4-5532)	-4.09 (-4.23~-3.96)
Panama	4499.9 (3487.4-5822.2)	5576.3 (4321.6-7215)	6893.3 (5553.5-8634.8)	2641.3 (2128-3308.6)	-2.42 (-2.65~-2.19)
Papua New Guinea	6915.1 (5286.4-8890.1)	11517.7 (8805-14807.2)	10549.4 (8134.4-13366.1)	6061.8 (4674.2-7680.4)	-1.8 (-1.9~-1.69)
Paraguay	7845.2 (6079.7-10234)	6765.6 (5243.1-8825.7)	14003.4 (10772.1-17445.8)	4638.6 (3568.2-5778.9)	-1.16 (-1.34~-0.98)
Peru	35417.5 (27124.5-45777.1)	5907.5 (4524.3-7635.5)	87657.4 (69103.6-108862.1)	4656 (3670.5-5782.3)	-1.24 (-1.6~-0.87)
Philippines	86886.9 (68226-110936.3)	6704.4 (5264.5-8560.2)	88541.8 (74830.9-104431.5)	2447.6 (2068.6-2886.9)	-3.06 (-3.23~-2.89)
Poland	256951.1 (187819.2-344784.9)	10678.2 (7805.2-14328.3)	255160.5 (197695.9-330076.7)	5462.9 (4232.6-7066.9)	-2.25 (-2.42~-2.08)
Portugal	32032.4 (26670.7-38599.5)	3726.4 (3102.7-4490.4)	49128.3 (43347.4-56673.8)	2776.3 (2449.6-3202.7)	-0.54 (-0.7~-0.39)
Puerto Rico	6735.9 (5650.6-8037.7)	3013.7 (2528.1-3596.1)	13311.2 (11601.8-15239.1)	2527.2 (2202.7-2893.3)	-0.73 (-0.97~-0.5)
Qatar	83.3 (65.8-104.6)	3451.8 (2724.8-4333.7)	507 (423.6-605.3)	2887 (2411.9-3446.9)	-0.29 (-0.43~-0.15)

Republic of Korea	32713 (27777.6-38864.9)	2577.1 (2188.3-3061.7)	88861.9 (75265.7-108205.8)	1603.9 (1358.5-1953.1)	-2.01 (-2.25~-1.76)
Republic of Moldova	4804.4 (3597.8-6362.4)	2287.3 (1712.8-3029.1)	5113.6 (3859.5-6961)	1467.4 (1107.5-1997.5)	-1.59 (-1.89~-1.29)
Romania	150015.7 (107690.8-201071.5)	10513 (7546.9-14090.9)	177743.2 (132760.8-234214.6)	7021.4 (5244.5-9252.2)	-1.54 (-1.81~-1.28)
Russian Federation	77749.8 (59921-100119.4)	805.2 (620.6-1036.9)	105214.6 (83406.2-132510.7)	735.4 (583-926.2)	-0.15 (-0.4~0.1)
Rwanda	37100.3 (29426.8-45149.1)	33599.1 (26649.8-40888.3)	36842.1 (27775.2-47577.7)	14910.3 (11240.8-19255)	-3 (-3.28~-2.72)
Saint Kitts and Nevis	128 (106.3-155.4)	4736.5 (3930.8-5750.5)	87.7 (75.8-100.3)	3155.2 (2726.8-3609.3)	-1.39 (-1.47~-1.31)
Saint Lucia	296.3 (238.2-371.1)	5667.9 (4556.2-7099.1)	522.2 (441.2-620.7)	3844.4 (3248.2-4569.7)	-1.32 (-1.39~-1.25)
Saint Vincent and the Grenadines	295.5 (240.5-363.9)	6771.5 (5511.8-8341.2)	329.8 (273.4-391.9)	4145.5 (3436.2-4926.1)	-1.71 (-1.81~-1.61)
Samoa	306.4 (243.2-395.5)	8085 (6419.1-10437.9)	313 (260.8-383.6)	4581 (3817.5-5614.7)	-1.88 (-2.01~-1.75)
San Marino	40.8 (32.2-52.9)	1746.7 (1378.3-2262.9)	101.1 (77.7-153.7)	1940.2 (1490.9-2950.7)	0.68 (0.44~0.92)
Sao Tome and Principe	815.6 (644.6-997.5)	24689.2 (19513.1-30194.1)	352.4 (266-461.7)	7687.6 (5803.4-10072.1)	-3.91 (-4.14~-3.67)
Saudi Arabia	8000.8 (6571.4-9718.6)	3222.9 (2647.1-3914.9)	11060.5 (9391.3-13490.5)	2131.2 (1809.6-2599.5)	-0.73 (-0.97~-0.48)
Senegal	60380.4 (51003.2-69362.5)	39927.6 (33726.7-45867.2)	29653.5 (22208.8-39380.7)	8375 (6272.4-11122.3)	-4.39 (-4.72~-4.06)
Serbia	122650.7 (92585.3-153135.6)	24695 (18641.5-30832.9)	121662.1 (96345.3-150682.4)	11252.8 (8911.2-13936.9)	-2.99 (-3.28~-2.69)
Seychelles	98.4 (81.4-117.5)	2876.2 (2379.2-3435.1)	85.6 (75-97.9)	1549.7 (1357.6-1773.6)	-1.72 (-1.86~-1.57)
Sierra Leone	41111.6 (33286.9-47462.8)	38055 (30812.1-43934)	24241.2 (18377.6-31179.4)	14137.5 (10717.9-18183.9)	-3.2 (-3.45~-2.94)
Singapore	2598.3 (2105.1-3195)	2482.4 (2011.2-3052.5)	7057.5 (5363.2-9247.6)	1460.6 (1110-1913.9)	-0.92 (-1.31~-0.53)
Slovakia	30449.8 (22368.7-39956.5)	9211.1 (6766.6-12086.9)	33358.6 (25271.1-44800.3)	5548.6 (4203.4-7451.7)	-1.73 (-1.83~-1.63)
Slovenia	8704.7 (6335.5-11725.2)	6356.4 (4626.3-8562)	12362.8 (9465.8-15685.2)	4114.4 (3150.3-5220.1)	-1.63 (-1.8~-1.46)
Solomon Islands	794.7 (599.5-1061.2)	16929.3 (12769.7-22605.6)	956.6 (749.4-1208.1)	7218.3 (5655.1-9116)	-2.57 (-2.74~-2.41)
Somalia	65023.5 (62410.9-67253.9)	92655.9 (88933.1-95834.1)	154184.6 (140343.6-164709.6)	80928.9 (73664-86453.3)	-0.41 (-0.46~-0.35)
South Africa	27300.3	2690.8	36626.5	1655.5	-1.44

	(21233.9-34792)	(2092.9-3429.2)	(30416-44055.8)	(1374.8-1991.3)	(-1.59~-1.28)
South Sudan	66909.4	53243.5	50308	36141	-1.49
	(57810.3-76953.9)	(46002.8-61236.5)	(40823.4-60722.1)	(29327.3-43622.4)	(-1.59~-1.39)
Spain	87198.5	2529.9	168457.6	2489.5	0.43
	(72333.7-105280.4)	(2098.7-3054.6)	(146051.9-197524.2)	(2158.4-2919.1)	(0.26~0.6)
Sri Lanka	39175.9	7944.2	49935.3	3282	-2.89
	(31122.8-47883.8)	(6311.1-9710)	(42579.5-58632.9)	(2798.5-3853.6)	(-3.14~-2.65)
Sudan	108408	23002.6	57896.7	6762.2	-3.95
	(83635.3-139300.5)	(17746.2-29557.5)	(44690-76253)	(5219.7-8906.2)	(-4.19~-3.71)
Suriname	671.9	5500.6	1228.9	3693.6	-1.52
	(528.8-838)	(4328.9-6860.2)	(990.2-1525.9)	(2976-4586.1)	(-1.71~-1.33)
Sweden	19168.6	1756.1	33388.4	2093.6	1.04
	(15326-24044.7)	(1404.1-2202.8)	(25229.2-49024.7)	(1581.9-3074)	(0.81~1.26)
Switzerland	9480.5	1359.2	30503.7	2444.9	2.34
	(7296-12089.8)	(1046-1733.2)	(23006.7-46483)	(1844-3725.7)	(2.07~2.6)
Syrian Arab Republic	22807.5	10237.4	27271.4	4618.3	-2.47
	(17742-28781.9)	(7963.7-12919.1)	(21796.7-34204.6)	(3691.2-5792.4)	(-2.64~-2.31)
Taiwan (Province of China)	25377.4	3479.7	58062.1	2299	-1.07
	(22164-29027.5)	(3039-3980.1)	(48336.9-70162.9)	(1913.9-2778.2)	(-1.3~-0.85)
Tajikistan	12806.2	9999.3	16034.4	8059.6	-0.7
	(9600.5-16373.6)	(7496.2-12784.8)	(12095.9-21296.5)	(6079.9-10704.5)	(-1.1~-0.3)
Thailand	138850.1	9083.6	227509.8	3651.8	-2.88
	(111899.5-174074.7)	(7320.5-11388)	(193398.2-264068.8)	(3104.2-4238.6)	(-3.07~-2.7)
Timor-Leste	1188.7	13932	1555.5	3439.8	-4.36
	(981.7-1448.9)	(11505.6-16981.3)	(1329.9-1820.4)	(2941.1-4025.6)	(-4.64~-4.08)
Togo	14863	29317.6	16006.8	11075.2	-2.89
	(11841.6-18130.2)	(23357.9-35762.3)	(12235.2-20582.4)	(8465.6-14241.1)	(-3.01~-2.76)
Tokelau	4.7	6205.3	2.9	3146	-2.28
	(3.7-6)	(4918-7901.5)	(2.5-3.3)	(2707.3-3627.4)	(-2.39~-2.16)
Tonga	183.3	7059.2	154.2	3518.6	-2.13
	(141.7-237.1)	(5455.1-9128.3)	(130.2-184.2)	(2971.4-4204.1)	(-2.33~-1.93)
Trinidad and Tobago	1896.8	4018	2660.7	2377.8	-1.95
	(1631.1-2239.6)	(3455.1-4744.2)	(2287.5-3122.9)	(2044.3-2791)	(-2.02~-1.87)
Tunisia	18290.9	7566	18097.4	2599.3	-3.35
	(14153.2-24668.3)	(5854.4-10204)	(14604.7-22162.1)	(2097.6-3183)	(-3.46~-3.23)
Türkiye	199300.2	13286.5	223221.6	4390.8	-3.47
	(154983.1-254350.6)	(10332.1-16956.5)	(178473.1-287377.6)	(3510.6-5652.7)	(-3.54~-3.4)
Turkmenistan	3209.7	3791.1	4343.1	2772.5	-1.13
	(2379.3-4178)	(2810.2-4934.8)	(3409.4-5413.6)	(2176.5-3456)	(-1.55~-0.71)
Tuvalu	28.9	10691	22.2	4350.8	-2.69
	(22-38)	(8151-14079.3)	(18-28)	(3530.2-5504.7)	(-2.89~-2.5)
Uganda	115126.5	39334.9	59782.6	9935.4	-4.7
	(95445.5-135829)	(32610.5-46408.2)	(44915.8-76697.2)	(7464.6-12746.4)	(-4.83~-4.56)

Ukraine	85917<br>(66907.2-114589.1)	2143.4<br>(1669.2-2858.7)	76538.8<br>(59691.6-97302.1)	1569.5<br>(1224-1995.3)	-1.09<br>(-1.28~-0.9)
United Arab Emirates	625.8<br>(495.2-791.3)	5142.4<br>(4069-6502.2)	1326.3<br>(1031.3-1635.5)	2747.3<br>(2136.2-3387.7)	-0.79<br>(-1.24~-0.34)
United Kingdom	93788.8<br>(75304-116950.2)	1507.2<br>(1210.2-1879.5)	103387.8<br>(81240-129865.7)	1130<br>(888-1419.5)	-0.26<br>(-0.52~0)
United Republic of Tanzania	148452.6<br>(117806.4-180666.4)	30471.5<br>(24181.1-37083.8)	135817.3<br>(105069.2-173593.9)	12228.3<br>(9459.9-15629.5)	-3.02<br>(-3.29~-2.76)
United States of America	475184.6<br>(360456.8-628842.1)	2240.5<br>(1699.6-2965)	862660.2<br>(680632.6-1072606.8)	2241.5<br>(1768.5-2787)	0.14<br>(-0.1~0.37)
United States Virgin Islands	124<br>(104.6-146.9)	3094.3<br>(2611.8-3667.5)	298.3<br>(258.5-341.9)	2313.5<br>(2004.8-2651.6)	-1.2<br>(-1.32~-1.08)
Uruguay	7495.5<br>(5930.5-9306.8)	3053.6<br>(2416-3791.5)	9827.5<br>(8502.6-11347.2)	2615.3<br>(2262.7-3019.7)	-0.42<br>(-0.7~-0.14)
Uzbekistan	28439.1<br>(21868.5-36869)	4986.3<br>(3834.2-6464.3)	27046.6<br>(20926.5-33578.3)	2836.6<br>(2194.7-3521.6)	-1.88<br>(-2.03~-1.74)
Vanuatu	355.3<br>(265.7-476.1)	15437.9<br>(11545.6-20688.3)	454.4<br>(352.7-586.3)	6826.1<br>(5298.1-8806.2)	-2.62<br>(-2.71~-2.52)
Venezuela (Bolivarian Republic of)	44932.1<br>(35032.1-56859.9)	9465<br>(7379.6-11977.7)	99034.1<br>(76084.2-127315.8)	6166.8<br>(4737.7-7927.8)	-1.59<br>(-1.79~-1.4)
Viet Nam	29446.8<br>(22802.8-37684.8)	1444.2<br>(1118.3-1848.2)	43644<br>(33799.4-55146.8)	976.9<br>(756.5-1234.3)	-0.84<br>(-1.02~-0.65)
Yemen	68385.2<br>(56112.8-83401.7)	35054.6<br>(28763.7-42752.1)	63208.8<br>(48287.5-81496.5)	10626<br>(8117.6-13700.3)	-3.99<br>(-4.1~-3.88)
Zambia	40554<br>(32355.9-49007.2)	35009.8<br>(27932.4-42307.4)	31198.1<br>(22772.8-41017)	11594.2<br>(8463.1-15243.2)	-3.82<br>(-4.19~-3.46)
Zimbabwe	22197.3<br>(16887.3-29023.1)	12377.4<br>(9416.6-16183.6)	27881.3<br>(20751.2-37334.4)	10503.1<br>(7817.2-14064.2)	0.05<br>(-0.23~0.33)

eTable 6. The DALYs number and DALYs rate of malnutrition among individuals over 70 in 1990 and 2021, and its temporal trends from 1990 to 2021, at the national level

Abbreviations: EAPC, estimated annual percentage change.

Location	1990		2021		1990-2021
	Case number (95% UI)	Rate (95% UI)	Case number (95% UI)	Rate (95% UI)	EAPC% (95% CI)
Afghanistan	2461.6 (1670-3420.7)	792.1 (537.4-1100.7)	2524.9 (1677.8-3615.5)	624.1 (414.7-893.7)	-0.95 (-1.15~-0.74)
Albania	430.4 (297.7-600)	431.1 (298.2-601.1)	670.2 (447.9-939.3)	247.1 (165.2-346.4)	-2 (-2.09~-1.91)
Algeria	2922.1 (1936.8-4341)	469.2 (311-697)	5774.5 (3766.5-8674.9)	333.8 (217.7-501.4)	-1.29 (-1.38~-1.2)
American Samoa	13.7 (10.9-16.6)	1555.1 (1246.9-1890.2)	29.4 (22.8-38.9)	1326.7 (1028.2-1755)	-0.44 (-0.66~-0.22)
Andorra	1.9 (1.1-3)	58.6 (33.4-94.8)	8.5 (3.1-16.9)	90.7 (33.7-181.2)	2.07 (1.72~2.42)
Angola	5487.2 (4113.3-7022.4)	4278.6 (3207.3-5475.7)	5877.6 (4630.1-7342.8)	1464.7 (1153.8-1829.8)	-3.85 (-4.02~-3.69)
Antigua and Barbuda	37.5 (33.3-42.3)	1029.5 (915.9-1163.1)	32.3 (28.3-37.3)	574.9 (504.2-663.8)	-2.5 (-3.21~-1.79)
Argentina	10672 (9132.8-12776.9)	577.1 (493.9-690.9)	14570.4 (12084.8-17831.1)	408.9 (339.1-500.4)	-1.21 (-1.76~-0.66)
Armenia	344.4 (232.2-510.5)	288.3 (194.4-427.3)	418.1 (244.2-689.5)	171 (99.9-282)	-2.32 (-2.54~-2.1)
Australia	1522 (1149.6-1988.6)	125.8 (95-164.4)	3416.6 (2474.8-4747.8)	110.8 (80.2-154)	-0.51 (-0.64~-0.38)
Austria	629.6 (393.2-957.2)	82.6 (51.6-125.6)	1128.6 (675.5-2063.4)	90.7 (54.3-165.9)	0.72 (0.4~1.03)
Azerbaijan	517.7 (322.7-770.7)	230.5 (143.7-343.1)	589.7 (347.7-944.7)	153.9 (90.7-246.6)	-1.62 (-1.87~-1.36)
Bahamas	44.9 (38.1-53.8)	555.2 (471.9-666)	67.8 (52.3-86.4)	349.7 (269.7-445.8)	-1.52 (-2.03~-1)
Bahrain	32.3 (25.2-42.8)	565.2 (440.5-747.4)	72.8 (50.3-105.9)	289.1 (199.6-420.2)	-2.47 (-2.65~-2.29)
Bangladesh	111896.1 (86720-139361.8)	4917.8 (3811.3-6124.9)	84677.1 (57451.9-116653)	1149 (779.6-1582.9)	-5.28 (-5.52~-5.03)
Barbados	136.7 (120.8-157.8)	639.6 (565.4-738.5)	92.7 (72.7-117.1)	289.1 (226.8-365.3)	-3.18 (-3.44~-2.92)
Belarus	1238.3 (791.3-1838.7)	178 (113.7-264.3)	1321.1 (883.4-1918.3)	138.5 (92.6-201)	-1.2 (-1.41~-0.99)
Belgium	1458.5 (1134.4-1901.2)	151.8 (118.1-197.9)	4581.5 (2792.4-9375.1)	284.9 (173.7-583.1)	2.52 (2.28~2.76)
Belize	66.6	1263.5	112.7	807.2	-1.82

	(58.8-75.6)	(1116.8-1435.5)	(95.3-133)	(682.5-952.7)	(-2.4~-1.24)
Benin	2185.2	2130.1	2493.7	1114.2	-2.02
	(1724.5-2711.9)	(1681.1-2643.6)	(1838.2-3330.7)	(821.3-1488.2)	(-2.1~-1.94)
Bermuda	8	229.2	11.9	131.6	-2
	(6.5-9.9)	(187.8-285)	(8.7-17.3)	(97-192)	(-2.65~-1.34)
Bhutan	132.5	1477.8	330.4	1024.9	-1.37
	(91.2-183)	(1017.6-2041.1)	(213.2-485.8)	(661.4-1507)	(-1.44~-1.3)
Bolivia	5439.8	3623.9	6747.4	1458.1	-2.86
(Plurinational State of)	(4101.1-6953.6)	(2732.1-4632.4)	(5196-8553.2)	(1122.8-1848.3)	(-3.02~-2.69)
Bosnia and	437.1	260.7	806.6	207.6	-1.03
Herzegovina	(276-645.4)	(164.6-385)	(528.5-1178.4)	(136-303.3)	(-1.24~-0.81)
Botswana	215.9	939.9	296.4	506.2	-1.98
	(157.5-283.6)	(685.6-1234.5)	(221.8-396)	(378.9-676.3)	(-2.12~-1.84)
Brazil	43215.3	1016.8	70795.8	503.5	-2.08
	(38456.8-49609.7)	(904.8-1167.2)	(60087.5-85659)	(427.3-609.2)	(-2.36~-1.8)
Brunei	13.2	296.5	25.4	185.9	-1.38
Darussalam	(9.2-18.7)	(206.9-420.3)	(16.9-37.5)	(124-274.7)	(-1.55~-1.21)
Bulgaria	1476.6	223.9	2316.9	225.1	0.01
	(910.6-2333.9)	(138.1-353.9)	(1503.4-3357.8)	(146-326.2)	(-0.05~0.07)
Burkina Faso	4700.3	2394.2	5819.7	1381.7	-1.89
	(3815.3-5869.7)	(1943.4-2989.8)	(4427.8-7276.9)	(1051.2-1727.7)	(-2.18~-1.61)
Burundi	7038.3	6220.5	4055.9	2251.5	-4.17
	(4902.3-10193.7)	(4332.7-9009.3)	(3083.9-5252.2)	(1711.9-2915.5)	(-4.54~-3.79)
Cabo Verde	211.4	1413.8	138.6	676.8	-2.44
	(169.4-256.2)	(1133.2-1713.1)	(107.5-181.3)	(525-884.9)	(-3.02~-1.87)
Cambodia	7347	3939.7	6623.4	1210.7	-4.22
	(5322.1-10002.4)	(2853.8-5363.5)	(5151.9-8335.7)	(941.7-1523.7)	(-4.4~-4.05)
Cameroon	1968.7	1069.3	3893.3	778.2	-1.07
	(1472.2-2541.5)	(799.7-1380.5)	(2715.1-5377.2)	(542.7-1074.8)	(-1.22~-0.92)
Canada	2957.6	147	14391.6	298.7	2.42
	(2353.3-3708.1)	(117-184.4)	(6683.6-24518.9)	(138.7-508.8)	(2~2.84)
Central African	794.3	2137.8	1561.9	2447.9	0.51
Republic	(543.1-1116.3)	(1461.6-3004.4)	(1177.3-1985.3)	(1845.2-3111.6)	(0.23~0.79)
Chad	4029.1	2675.2	3696.1	1550.9	-1.69
	(3172.6-5026)	(2106.5-3337.1)	(2774.5-4799.7)	(1164.2-2014)	(-1.91~-1.47)
Chile	2808.3	519.8	6813.4	437.4	-0.56
	(2366.4-3316.5)	(438-613.8)	(5059.6-9453.6)	(324.8-606.9)	(-1.24~0.13)
China	293516.1	782.1	425248	356.4	-2.71
	(236949.5-366835.7)	(631.4-977.5)	(329188.6-548937.7)	(275.9-460.1)	(-2.92~-2.51)
Colombia	9877.1	1180.8	14116.4	443	-3.4
	(8977-10901.1)	(1073.2-1303.2)	(12002.4-16697.9)	(376.6-524)	(-3.6~-3.2)
Comoros	285.8	3527.2	325.9	1436.4	-3.25
	(223.1-356.5)	(2753.5-4400.3)	(251.2-422.7)	(1107.2-1862.9)	(-3.48~-3.01)

Congo	730.1<br>(549.3-923.5)	1674.4<br>(1259.9-2118)	1247.1<br>(976-1599.9)	1296.7<br>(1014.8-1663.6)	-1.02<br>(-1.31~-0.73)
Cook Islands	2.7<br>(1.7-4)	451.7<br>(289.2-679.5)	5.2<br>(3.2-7.9)	332.3<br>(207.2-505.3)	-0.91<br>(-1.05~-0.78)
Costa Rica	442.7<br>(369.1-546.6)	467.4<br>(389.6-577.1)	895.1<br>(609.8-1153.3)	285.4<br>(194.5-367.8)	-1.65<br>(-1.75~-1.56)
Côte d’Ivoire	1778.8<br>(1342.5-2276.2)	1329.4<br>(1003.3-1701.1)	3345.3<br>(2473.1-4371.4)	802.3<br>(593.1-1048.4)	-1.54<br>(-1.65~-1.43)
Croatia	511<br>(324.3-758.2)	180.3<br>(114.4-267.5)	974.5<br>(634.3-1476.5)	158.5<br>(103.2-240.1)	-0.54<br>(-0.62~-0.45)
Cuba	1198.5<br>(885.7-1640.7)	191.5<br>(141.5-262.2)	2196.2<br>(1540.5-3176.5)	176.9<br>(124.1-255.8)	-0.68<br>(-1.05~-0.31)
Cyprus	66.3<br>(46.5-97.2)	128.5<br>(90.2-188.5)	115.2<br>(83-154.9)	85.1<br>(61.3-114.4)	-1.42<br>(-1.57~-1.28)
Czechia	1599<br>(1075.9-2311.4)	200.6<br>(135-290)	4036.2<br>(3217.7-5240.5)	261.3<br>(208.3-339.3)	1.16<br>(0.72~1.6)
Democratic People's Republic of Korea	6482.7<br>(4621.1-8880.6)	990.1<br>(705.8-1356.3)	11102.7<br>(7804.8-15251.9)	634.4<br>(446-871.5)	-8.77<br>(-13.09~-4.23)
Democratic Republic of the Congo	11767.7<br>(8726.2-15214.1)	2079.6<br>(1542.1-2688.6)	17438.3<br>(11979.8-24090.9)	1379.1<br>(947.4-1905.3)	-1.48<br>(-1.85~-1.1)
Denmark	1353.2<br>(1150.7-1585.7)	242.1<br>(205.9-283.7)	1524.7<br>(1224.8-2578.6)	177.4<br>(142.5-299.9)	-1<br>(-1.26~-0.75)
Djibouti	153.6<br>(112.6-217.5)	3614.7<br>(2650.3-5117.2)	415.5<br>(320.7-561.3)	1897.2<br>(1464.5-2562.9)	-2.22<br>(-2.39~-2.05)
Dominica	32.3<br>(26.8-39)	836.5<br>(695.9-1010.2)	20.6<br>(16.1-26.6)	457.6<br>(356.1-589.2)	-2.1<br>(-2.43~-1.77)
Dominican Republic	2028.6<br>(1717.3-2423.7)	1100.2<br>(931.4-1314.5)	4174.1<br>(3222.9-5369.2)	752.2<br>(580.8-967.6)	-0.83<br>(-1.09~-0.57)
Ecuador	6557.6<br>(5977.8-7047.2)	2421.5<br>(2207.4-2602.2)	5264.7<br>(4384.9-6256.5)	563.1<br>(469-669.2)	-4.27<br>(-4.6~-3.93)
Egypt	10714.1<br>(8436.5-13634.3)	1090<br>(858.3-1387.1)	9268.1<br>(5753.7-14510.4)	408.2<br>(253.4-639.2)	-3.34<br>(-3.61~-3.08)
El Salvador	2358.6<br>(2017.8-2685.5)	1488.6<br>(1273.5-1694.9)	3231.1<br>(2658.3-3947)	843.8<br>(694.2-1030.7)	-1.94<br>(-2.14~-1.75)
Equatorial Guinea	166.1<br>(120.7-216.3)	2213.9<br>(1608.3-2882.8)	164.4<br>(120.5-215.7)	858.9<br>(629.5-1126.9)	-3.83<br>(-4.2~-3.45)
Eritrea	2712.8<br>(1892.8-3815.1)	9632.6<br>(6721-13546.9)	2240.9<br>(1794.5-2732.2)	2441<br>(1954.8-2976.2)	-4.48<br>(-4.73~-4.23)
Estonia	215.4<br>(147.3-319.1)	181.6<br>(124.1-268.9)	233.6<br>(151.2-368.5)	122.1<br>(79-192.6)	-1.64<br>(-1.94~-1.33)
Eswatini	104.7	885.5	140.9	626.7	-0.97

	(77.5-136.8)	(655.3-1157.2)	(100.6-189.5)	(447.3-842.8)	(-1.29~-0.64)
Ethiopia	49318.8	6381.4	32367.2	1641.7	-5.07
	(38296.6-60857.4)	(4955.2-7874.4)	(27013.5-39176.6)	(1370.1-1987.1)	(-5.35~-4.79)
Fiji	153.1	1132	265	823.8	-1.29
	(119.3-202.7)	(881.8-1498.8)	(189-365.2)	(587.6-1135.1)	(-1.44~-1.15)
Finland	447.8	97.9	848.6	90.6	-0.2
	(299-630.4)	(65.4-137.8)	(605.8-1293)	(64.7-138)	(-0.37~-0.02)
France	27781.4	533.9	59728.6	601.3	0.54
	(24494.3-31589.6)	(470.7-607.1)	(44188.7-96627.3)	(444.9-972.8)	(0.34~0.75)
Gabon	444.9	1576.3	432.4	1067.2	-1.38
	(333.4-586)	(1181.4-2076.4)	(321.7-577.7)	(793.9-1425.7)	(-1.63~-1.13)
Gambia	297.4	1927.9	572.9	1278	-1.52
	(240.7-365.7)	(1559.9-2370.1)	(434.9-731.5)	(970.2-1631.8)	(-1.62~-1.42)
Georgia	772.9	234.1	696.9	186.8	-0.87
	(435-1223.8)	(131.8-370.7)	(345.8-1181.1)	(92.7-316.6)	(-0.95~-0.79)
Germany	8104.9	100.3	31815.3	234.7	4.19
	(5114.2-12070.7)	(63.3-149.3)	(12105.5-47260)	(89.3-348.6)	(3.78~4.6)
Ghana	6796.3	2682	11016.4	1600.3	-1.47
	(5362.1-8567.3)	(2116-3380.9)	(8525-14341.2)	(1238.4-2083.2)	(-1.55~-1.39)
Greece	746.5	78.8	1550.7	89.6	0.62
	(444.4-1236.7)	(46.9-130.6)	(891.4-2831.6)	(51.5-163.6)	(0.42~0.81)
Greenland	4.6	373.1	7.9	262.7	-0.95
	(3.8-5.8)	(302.4-470.9)	(6-11.4)	(198-379.4)	(-1.36~-0.54)
Grenada	44.5	894.5	38.1	645.3	-1.76
	(38.5-52.3)	(774.2-1051.8)	(32.1-44.4)	(543.4-750.7)	(-2.4~-1.11)
Guam	22.6	812.2	66	539.1	-1.13
	(18.1-27.5)	(648.8-986.8)	(45.3-91.3)	(370.6-746.2)	(-1.49~-0.76)
Guatemala	27785.8	18937.6	8894.7	1508.9	-8.36
	(26117.8-29244.6)	(17800.8-19931.9)	(7781.7-10302.2)	(1320.1-1747.7)	(-8.66~-8.05)
Guinea	2611.8	1575.1	3197	1231.9	-0.72
	(1954.3-3366.8)	(1178.6-2030.5)	(2368.5-4167.9)	(912.7-1606)	(-0.93~-0.51)
Guinea-Bissau	391.5	2393.9	309.2	1218.1	-2.1
	(310.2-512)	(1896.9-3130.5)	(225.7-409)	(889.3-1611.3)	(-2.12~-2.08)
Guyana	489.3	2788.2	306.5	1059.1	-3.04
	(440.2-540.9)	(2508.4-3082.3)	(251.9-376.5)	(870.5-1301.2)	(-3.44~-2.65)
Haiti	1517.6	1147.5	1934.5	680.7	-1.67
	(1135.2-1975.8)	(858.4-1494.1)	(1378.9-2659.4)	(485.2-935.7)	(-1.74~-1.6)
Honduras	1330.8	1363.9	3489.3	1107.2	-0.69
	(1058.4-1630.1)	(1084.7-1670.6)	(2708.4-4355.8)	(859.4-1382.2)	(-0.9~-0.47)
Hungary	1811.5	213.7	2739.3	203.4	0.06
	(1182.1-2580.3)	(139.4-304.4)	(1887.6-3822.9)	(140.2-283.9)	(-0.14~0.26)
Iceland	17.8	97.5	52.2	137.9	1.22
	(13.2-24.7)	(72.3-135)	(36.6-116.8)	(96.9-308.8)	(0.94~1.5)
India	472949.5	2625.8	1134460.2	1904	-1

	(370970.1-602707.4)	(2059.6-3346.2)	(823993.4-1512117.5)	(1382.9-2537.8)	(-1.05~-0.96)
Indonesia	144394.3	3791.8	243460.7	2524	-0.87
	(117664.2-173198.3)	(3089.9-4548.2)	(201813.5-279609.2)	(2092.2-2898.7)	(-1.09~-0.66)
Iran (Islamic	4133.1	426	9369.2	256.6	-1.24
Republic of)	(2750.4-6096.2)	(283.5-628.3)	(6425.8-14191.6)	(176-388.6)	(-1.42~-1.07)
Iraq	2146.2	537.2	3227.4	325.4	-1.92
	(1625.7-2896.1)	(407-725)	(2332-4549.2)	(235.1-458.7)	(-2.02~-1.81)
Ireland	302.3	112.5	552.5	106.3	0.13
	(218.3-421)	(81.2-156.7)	(299.7-1025.1)	(57.7-197.2)	(-0.12~0.38)
Israel	424.9	139.8	1518.8	184	1.34
	(290.8-607.9)	(95.6-199.9)	(867.3-2983.4)	(105-361.4)	(1.16~1.51)
Italy	5847.3	105.6	18792.6	178.7	2.05
	(3580.9-9342.8)	(64.6-168.7)	(12205.9-27596.7)	(116.1-262.4)	(1.93~2.18)
Jamaica	1075.4	924.4	987.3	542.8	-2.16
	(952.8-1224.5)	(819.1-1052.5)	(789.6-1243.1)	(434.2-683.5)	(-2.61~-1.71)
Japan	13632.9	138	59303.8	205.2	1.34
	(9801.5-19113)	(99.2-193.5)	(41737.8-84527.7)	(144.4-292.5)	(1.21~1.46)
Jordan	268.6	547.6	1163.8	379	-1.42
	(179.9-392.1)	(366.8-799.5)	(748.4-1690)	(243.7-550.3)	(-1.53~-1.32)
Kazakhstan	2353.6	392.3	2083.2	256.1	-2.24
	(1380.9-3711.4)	(230.2-618.6)	(1213.9-3333.1)	(149.3-409.8)	(-2.58~-1.91)
Kenya	6984.5	1885	11921.3	1277.6	-0.99
	(5720.7-8331.2)	(1543.9-2248.4)	(9319-15066.8)	(998.7-1614.7)	(-1.17~-0.81)
Kiribati	54.3	3764.3	72.8	2924.3	-0.85
	(40.8-70.4)	(2828.9-4881.8)	(56.3-94.3)	(2264.5-3788.6)	(-0.9~-0.81)
Kuwait	37.8	189.3	138.8	140.6	-0.87
	(24.8-56.8)	(124-284.6)	(86.2-210.4)	(87.4-213.1)	(-1.1~-0.63)
Kyrgyzstan	511	353.4	452.3	235.6	-1.84
	(301.2-768.4)	(208.3-531.3)	(227.6-761)	(118.6-396.5)	(-2.05~-1.63)
Lao People's	2441.1	2750.8	2238.6	1162.1	-2.88
Democratic	(1858.3-3129)	(2094.1-3526)	(1690.1-2881.4)	(877.4-1495.8)	(-2.97~-2.79)
Republic					
Latvia	376.8	181.6	395.8	140.6	-0.96
	(245.5-557.7)	(118.3-268.8)	(254.8-585.5)	(90.5-207.9)	(-1.16~-0.76)
Lebanon	344.5	360.2	932.8	242.6	-1.12
	(238.5-473.8)	(249.4-495.4)	(639.8-1380.9)	(166.4-359.1)	(-1.21~-1.04)
Lesotho	561.7	1309.3	455.5	947.4	-0.68
	(417.7-722.9)	(973.8-1685.3)	(347.1-600.4)	(721.9-1248.8)	(-0.92~-0.43)
Liberia	1347.3	2264.4	1055.7	1323.8	-2.34
	(1079.9-1680.1)	(1815.1-2823.7)	(805.4-1359.7)	(1009.9-1705)	(-2.61~-2.07)
Libya	280.6	322.9	645.4	294.5	-0.14
	(194.5-392.9)	(223.8-452.1)	(430.4-909.9)	(196.4-415.2)	(-0.34~0.06)
Lithuania	430.3	169.5	521.9	129.9	-0.93
	(265.7-641.3)	(104.7-252.7)	(319-806.7)	(79.4-200.9)	(-1.1~-0.75)

Luxembourg	61.7	180.7	141.5	212.9	0.82
	(49.6-78.3)	(145.4-229.2)	(116.3-168.8)	(175-253.9)	(0.55~1.09)
Madagascar	13206.1	6151.8	10026.4	2756.5	-2.34
	(11317.1-15433.6)	(5271.9-7189.4)	(7720.6-12751.1)	(2122.6-3505.6)	(-2.44~-2.24)
Malawi	4973.7	3197.9	5453.6	1753.1	-2.38
	(3928.2-6236)	(2525.7-4009.5)	(4372.3-6694.5)	(1405.5-2151.9)	(-2.59~-2.18)
Malaysia	4208.5	967.1	8899.8	629.4	-1.75
	(3173.7-5428.2)	(729.3-1247.4)	(6507.8-11619.7)	(460.2-821.7)	(-1.86~-1.63)
Maldives	84.5	2818.1	81.6	599.9	-5.26
	(62.6-114)	(2086.3-3799.6)	(62.5-103.1)	(459.7-758)	(-5.96~-4.55)
Mali	4693.8	2888.6	10508.4	2846.4	0.01
	(3747.8-5820.9)	(2306.5-3582.2)	(8309.1-13256.7)	(2250.7-3590.8)	(-0.35~0.36)
Malta	26	104.8	60.6	84.4	-0.51
	(15.8-41.4)	(63.6-166.9)	(35.8-102.8)	(49.9-143.2)	(-0.62~-0.41)
Marshall Islands	11.7	1589.2	12.9	1126.5	-1.22
	(8.7-15.2)	(1180.7-2071.4)	(9.6-17.2)	(840.4-1495.5)	(-1.32~-1.12)
Mauritania	1445.1	2769.6	918.6	902.4	-3.4
	(1039-2128.4)	(1991.3-4079.2)	(671-1251.5)	(659.1-1229.4)	(-3.6~-3.2)
Mauritius	425.3	1291.4	624.8	623.1	-0.9
	(352.5-517.6)	(1070.6-1571.8)	(436.2-876.9)	(435-874.4)	(-1.32~-0.48)
Mexico	76476.8	3682.5	66247	980.5	-4.32
	(72339.1-79193.3)	(3483.2-3813.3)	(59598.8-73038.4)	(882.1-1081)	(-4.46~-4.18)
Micronesia (Federated States of)	40.2	1713.6	29.2	1157.4	-1.09
	(30.4-51.7)	(1292.8-2202.1)	(22-39)	(872.2-1543.9)	(-1.18~-1)
Monaco	3.6	73.6	7.5	104.8	1.41
	(1.8-9.4)	(36.7-190.3)	(2.9-15)	(40.7-210.3)	(1.07~1.75)
Mongolia	172.3	335.4	158.5	188.4	-2.25
	(98.9-270.7)	(192.4-526.8)	(86.8-262.8)	(103.2-312.3)	(-2.43~-2.07)
Montenegro	61	190.6	108.7	186.8	-0.3
	(39.2-89.2)	(122.4-278.6)	(68.3-162.6)	(117.4-279.4)	(-0.42~-0.17)
Morocco	4010.5	565.4	6769.2	409.6	-0.96
	(2888.8-5541.3)	(407.2-781.2)	(4621.1-9741.7)	(279.6-589.5)	(-1.01~-0.91)
Mozambique	8096.5	3331.5	8421.1	1958.9	-1.89
	(6067.1-10575.5)	(2496.4-4351.5)	(6375.7-10768.7)	(1483.1-2505)	(-2.02~-1.76)
Myanmar	27275.8	2788.7	22565.7	1009.8	-3.54
	(20427.7-34846)	(2088.6-3562.7)	(16456.1-29905.4)	(736.4-1338.2)	(-3.63~-3.44)
Namibia	361.4	1360.9	415.5	694	-2.35
	(285-449.2)	(1073.2-1691.7)	(320.1-527.7)	(534.6-881.4)	(-2.53~-2.16)
Nauru	2	1257.9	2.4	1054.6	-0.57
	(1.5-2.6)	(948.1-1652.5)	(1.8-3.3)	(773.9-1449.1)	(-0.87~-0.28)
Nepal	13571.6	3722.1	17692.2	1518.6	-2.83
	(10392.1-17118.2)	(2850.1-4694.8)	(13350.8-23161)	(1146-1988)	(-3.2~-2.46)
Netherlands	2310.4	180.1	6769.2	275.6	1.56

	(1405.2-4749.5)	(109.5-370.2)	(3056.2-11286.3)	(124.4-459.5)	(1.17~1.96)
New Zealand	341.6	138.1	558	99.7	-0.61
	(251.6-462)	(101.7-186.7)	(369.5-915)	(66.1-163.6)	(-0.94~-0.28)
Nicaragua	893.6	1214.2	1338.1	535.9	-2.78
	(741.5-1055.9)	(1007.5-1434.7)	(1031.9-1687.2)	(413.3-675.7)	(-2.95~-2.61)
Niger	1566	1523.2	2909.5	853.9	-2.18
	(1213.6-2006.8)	(1180.4-1952.1)	(2077.3-4001)	(609.7-1174.3)	(-2.32~-2.04)
Nigeria	15023.2	717	19461.8	519.8	-1.1
	(10827.4-20587.4)	(516.8-982.6)	(12790.4-28020.1)	(341.6-748.3)	(-1.17~-1.03)
Niue	2.1	1437.2	1.1	947.4	-1.49
	(1.7-2.7)	(1148.2-1809)	(0.8-1.5)	(715.5-1259.6)	(-1.67~-1.31)
North	227.2	257.8	383.4	201.8	-0.93
Macedonia	(143.9-336.1)	(163.3-381.4)	(247.7-569.4)	(130.4-299.7)	(-1.05~-0.81)
Northern	4.5	933.9	17.2	813.5	-0.04
Mariana Islands	(3.6-5.6)	(741.3-1166.5)	(13.5-22)	(637.1-1038.2)	(-0.28~0.19)
Norway	444	90.9	2548	368.1	6.51
	(305.3-638.2)	(62.5-130.6)	(2014.2-3385.5)	(290.9-489)	(5.06~7.98)
Oman	302.6	1048.4	297.4	430.8	-2.3
	(241.5-381.2)	(836.9-1320.8)	(235.8-375.7)	(341.5-544.1)	(-2.59~-2)
Pakistan	54948.5	1943.7	72812.2	1437.9	-1.12
	(42385.1-71234.6)	(1499.3-2519.8)	(51162.2-100595.7)	(1010.4-1986.6)	(-1.27~-0.97)
Palau	3.8	800.6	5.9	646.4	-0.39
	(2.9-5.2)	(599.1-1089.9)	(4.2-8.2)	(456.6-903.6)	(-0.58~-0.19)
Palestine	209.4	496.6	338.2	325.7	-1.29
	(146.8-289.7)	(348.1-686.9)	(221.5-502.1)	(213.4-483.7)	(-1.38~-1.21)
Panama	1125.5	1394.7	1346.9	516.1	-3.3
	(1005.4-1250.8)	(1245.9-1550)	(1080.2-1664.1)	(413.9-637.6)	(-3.44~-3.16)
Papua New	877.1	1460.8	2026.5	1164.4	-0.53
Guinea	(665.6-1135.9)	(1108.6-1892)	(1522.9-2716.5)	(875.1-1560.9)	(-0.63~-0.43)
Paraguay	732	631.2	2049.2	678.8	0.63
	(596.7-936.1)	(514.6-807.3)	(1552.1-2594.5)	(514.1-859.4)	(0.53~0.74)
Peru	10896.6	1817.5	17117.8	909.2	-2.98
	(9102.3-12907.5)	(1518.2-2152.9)	(13511-21345.6)	(717.7-1133.8)	(-3.42~-2.54)
Philippines	18058.1	1393.4	46179.7	1276.6	0.04
	(15272.8-21649.1)	(1178.5-1670.5)	(37711.9-56367.6)	(1042.5-1558.2)	(-0.08~0.17)
Poland	5653.9	235	9246.2	198	-0.63
	(3610.3-7993.1)	(150-332.2)	(6381.9-13300.2)	(136.6-284.8)	(-0.8~-0.47)
Portugal	1790.2	208.3	3358.7	189.8	-0.21
	(1370.9-2458.4)	(159.5-286)	(2623.4-4492.1)	(148.3-253.9)	(-0.44~0.02)
Puerto Rico	899	402.2	1559.5	296.1	-1.37
	(778.9-1054.6)	(348.5-471.8)	(1223.3-1984.5)	(232.2-376.8)	(-1.88~-0.85)
Qatar	8.5	353	40.7	231.7	-1.65
	(5.7-12.3)	(236-511.6)	(28.9-56.3)	(164.7-320.4)	(-1.96~-1.34)

Republic of Korea	4742.7 (3906.2-5751.5)	373.6 (307.7-453.1)	5962.8 (4477.2-7940.8)	107.6 (80.8-143.3)	-4.41 (-4.92~-3.9)
Republic of Moldova	563.9 (329.2-898.9)	268.5 (156.7-428)	649.1 (352.8-1055.7)	186.3 (101.2-302.9)	-1.37 (-1.47~-1.26)
Romania	3613 (2283.3-5335.2)	253.2 (160-373.9)	5451.3 (3734.1-7925.2)	215.3 (147.5-313.1)	-0.58 (-0.74~-0.41)
Russian Federation	18672.8 (11781-28697.7)	193.4 (122-297.2)	24623.2 (16492.9-37697.5)	172.1 (115.3-263.5)	-0.69 (-0.88~-0.5)
Rwanda	3706.7 (2966-4561.2)	3356.8 (2686.1-4130.8)	3344.8 (2566.5-4237.4)	1353.7 (1038.7-1714.9)	-4.21 (-4.69~-3.72)
Saint Kitts and Nevis	64.4 (58.8-70.5)	2381.9 (2174.6-2609.7)	36.3 (32.1-40.9)	1306.7 (1156.4-1472.4)	-1.87 (-2.58~-1.16)
Saint Lucia	63.7 (57.6-71.6)	1218.8 (1102-1369)	84.2 (69.5-101.6)	620.2 (511.9-748.2)	-2.85 (-3.71~-1.97)
Saint Vincent and the Grenadines	73.4 (66.8-80.5)	1682.6 (1530.6-1845.2)	96.6 (85.8-108.4)	1213.7 (1078.8-1362.8)	-1.49 (-2.31~-0.66)
Samoa	47 (37.1-59.8)	1241.3 (978.7-1577.6)	64.8 (48.7-86.4)	948.1 (712.2-1264)	-0.65 (-0.84~-0.46)
San Marino	2.3 (1.7-3.7)	98.9 (72.1-159.1)	5.5 (3.1-12.4)	106.5 (59-237.5)	0.75 (0.5~1)
Sao Tome and Principe	59.8 (48.9-72.1)	1809.4 (1481.6-2182.9)	55.4 (42.9-71.1)	1207.8 (935.5-1550)	-1.12 (-1.22~-1.02)
Saudi Arabia	1727.6 (1299-2297.9)	695.9 (523.3-925.7)	1674.2 (1165-2341.2)	322.6 (224.5-451.1)	-2.39 (-2.43~-2.36)
Senegal	2120.8 (1619.8-2744.1)	1402.4 (1071.1-1814.6)	2970.9 (2001.5-4176.7)	839.1 (565.3-1179.6)	-1.77 (-1.82~-1.72)
Serbia	1317.7 (853.7-1948.2)	265.3 (171.9-392.3)	2385.4 (1599-3407)	220.6 (147.9-315.1)	-0.67 (-0.75~-0.58)
Seychelles	41.3 (33.1-51.7)	1206.5 (968.7-1511.6)	42 (32.9-54.2)	760.9 (596.1-980.8)	-1.23 (-1.4~-1.06)
Sierra Leone	2263.8 (1823.8-2779.3)	2095.5 (1688.2-2572.7)	5130.2 (4063.3-6489.6)	2991.9 (2369.7-3784.8)	2.11 (1.74~2.49)
Singapore	144 (86.7-224.8)	137.6 (82.9-214.7)	316.8 (184-518.7)	65.6 (38.1-107.3)	-2.26 (-2.5~-2.01)
Slovakia	697.8 (457.3-1033.1)	211.1 (138.3-312.5)	1049.5 (664.4-1568.4)	174.6 (110.5-260.9)	-0.54 (-0.59~-0.5)
Slovenia	230.6 (140.7-345.3)	168.4 (102.8-252.2)	445.4 (309.5-632.1)	148.2 (103-210.4)	-0.66 (-0.83~-0.49)
Solomon Islands	75 (57-98.3)	1598.1 (1214.3-2094.6)	155.6 (113.5-211)	1174.1 (856.6-1592.1)	-0.85 (-0.98~-0.71)
Somalia	4611.3 (3034.8-6218.6)	6570.9 (4324.5-8861.2)	7017.9 (5033.1-9672.7)	3683.6 (2641.8-5077)	-0.79 (-3.34~1.84)
South Africa	6071.9	598.5	10163.8	459.4	-1.13

	(4761.9-7655)	(469.3-754.5)	(8123.2-12666.7)	(367.2-572.5)	(-1.45~-0.8)
South Sudan	7043.5	5604.9	3924.6	2819.4	-2.86
	(5340.8-9028.1)	(4250-7184.2)	(2961.9-5046.2)	(2127.8-3625.1)	(-3.09~-2.62)
Spain	4230.5	122.7	8142.2	120.3	0.09
	(3215.3-5794.9)	(93.3-168.1)	(6304.9-10830.5)	(93.2-160.1)	(-0.1~0.29)
Sri Lanka	6064.8	1229.8	8221.5	540.4	-2.62
	(4908.8-7626.9)	(995.4-1546.6)	(5641.8-11917.7)	(370.8-783.3)	(-2.69~-2.55)
Sudan	5067.1	1075.2	5292.5	618.2	-1.72
	(3706.8-6909.2)	(786.5-1466)	(3574.2-7587.3)	(417.5-886.2)	(-1.84~-1.59)
Suriname	112.9	924.6	135	405.8	-2.56
	(93.7-137.7)	(767.5-1127.3)	(97.2-179.6)	(292-539.9)	(-2.82~-2.3)
Sweden	1289	118.1	2674	167.7	1.28
	(942.2-1694.6)	(86.3-155.2)	(1803.1-4847.9)	(113.1-304)	(1.11~1.44)
Switzerland	1320.1	189.2	2428.7	194.7	1.04
	(1130.7-1576.2)	(162.1-226)	(1505.7-4801.2)	(120.7-384.8)	(0.43~1.65)
Syrian Arab Republic	1751.7	786.3	2860.7	484.4	-1.39
	(1345.8-2293.8)	(604.1-1029.6)	(2066.3-4057.1)	(349.9-687)	(-1.62~-1.15)
Taiwan (Province of China)	4498.5	616.8	7618.2	301.7	-2.14
	(3602.4-5687)	(493.9-779.8)	(5241.6-11022.5)	(207.5-436.4)	(-2.8~-1.47)
Tajikistan	465.3	363.3	717.4	360.6	-0.22
	(284.6-717.7)	(222.3-560.4)	(390.4-1162.9)	(196.2-584.5)	(-0.32~-0.13)
Thailand	21427.2	1401.8	33805	542.6	-3.54
	(16674.4-26784.7)	(1090.8-1752.3)	(24793.4-44666.9)	(398-716.9)	(-3.91~-3.16)
Timor-Leste	364.4	4270.4	791.4	1750.1	-2.91
	(235-584.6)	(2754-6851.9)	(583.5-1088.3)	(1290.3-2406.6)	(-3.09~-2.72)
Togo	787.6	1553.5	1468.3	1015.9	-1.45
	(593.7-1009.1)	(1171.1-1990.4)	(1069.4-2022.3)	(740-1399.2)	(-1.53~-1.37)
Tokelau	1.1	1464.1	0.9	928.7	-1.35
	(0.9-1.4)	(1170.9-1829.6)	(0.7-1.1)	(716-1218.3)	(-1.53~-1.17)
Tonga	31.9	1228.4	53.7	1225.9	0.19
	(24.9-40.4)	(958.5-1556.6)	(42.7-69.2)	(973.6-1579.6)	(0.11~0.26)
Trinidad and Tobago	397.3	841.6	344.2	307.6	-3.69
	(346.7-463.1)	(734.4-981.1)	(262-466.4)	(234.1-416.9)	(-4~-3.38)
Tunisia	913.8	378	2144.2	308	-0.57
	(602.3-1315.1)	(249.1-544)	(1374.2-2995.9)	(197.4-430.3)	(-0.63~-0.5)
Türkiye	8695	579.7	24229.4	476.6	-0.14
	(6485.4-11659)	(432.4-777.3)	(17926.9-33656.5)	(352.6-662)	(-0.49~0.21)
Turkmenistan	217.3	256.7	274.3	175.1	-1.42
	(134.3-329.7)	(158.6-389.4)	(153.4-441.9)	(97.9-282.1)	(-1.49~-1.35)
Tuvalu	4.4	1644.7	5.3	1041.8	-1.18
	(3.4-5.6)	(1259.1-2078.7)	(4-6.9)	(787.3-1358.4)	(-1.29~-1.06)
Uganda	6771.4	2313.6	7904.9	1313.7	-2.33
	(5264.8-8616.9)	(1798.8-2944.1)	(6225-10212.9)	(1034.5-1697.3)	(-2.55~-2.11)

Ukraine	7047.3<br>(4302.7-10834.7)	175.8<br>(107.3-270.3)	7500.4<br>(4495.5-11545.6)	153.8<br>(92.2-236.8)	-0.55<br>(-0.69~-0.42)
United Arab Emirates	56.1<br>(39.5-77.5)	460.6<br>(324.7-637.2)	164.9<br>(107.4-256.2)	341.5<br>(222.4-530.7)	-0.08<br>(-0.42~0.26)
United Kingdom	5718.2<br>(3885.3-8335.9)	91.9<br>(62.4-134)	7123.5<br>(4455-10767.8)	77.9<br>(48.7-117.7)	-0.3<br>(-0.48~-0.13)
United Republic of Tanzania	13888.9<br>(11321-16880.9)	2850.9<br>(2323.8-3465)	14782.6<br>(11592.1-18575.6)	1331<br>(1043.7-1672.4)	-2.98<br>(-3.17~-2.78)
United States of America	39217.9<br>(31576.3-50614.2)	184.9<br>(148.9-238.6)	187335.9<br>(153124.6-218808.8)	486.8<br>(397.9-568.5)	2.77<br>(2.41~3.13)
United States Virgin Islands	21.1<br>(16.7-27)	527.6<br>(417.3-674.8)	33.1<br>(25.1-43.1)	256.6<br>(194.5-334.4)	-2.73<br>(-2.93~-2.52)
Uruguay	1018.3<br>(836.1-1247)	414.8<br>(340.6-508)	1389.3<br>(1179.6-1641.7)	369.7<br>(313.9-436.9)	-0.35<br>(-0.74~0.05)
Uzbekistan	2414.6<br>(1524.8-3541.1)	423.4<br>(267.3-620.9)	2657.8<br>(1527.5-4387.3)	278.7<br>(160.2-460.1)	-1.88<br>(-2.08~-1.68)
Vanuatu	36.6<br>(26.8-46.9)	1589.5<br>(1166.5-2036.3)	87.9<br>(66.5-118.7)	1320<br>(998.8-1782.7)	-0.57<br>(-0.64~-0.5)
Venezuela (Bolivarian Republic of)	4868<br>(4294.9-5584.2)	1025.4<br>(904.7-1176.3)	7983.7<br>(6405.9-9935.7)	497.1<br>(398.9-618.7)	-2.9<br>(-3.22~-2.57)
Viet Nam	27217.9<br>(20138.1-35682.4)	1334.9<br>(987.6-1750)	28625.2<br>(20771.2-38778.3)	640.7<br>(464.9-868)	-2.44<br>(-2.6~-2.29)
Yemen	3002.3<br>(2253.1-4006)	1539<br>(1155-2053.5)	7392.8<br>(5413.9-9771.5)	1242.8<br>(910.1-1642.7)	-0.88<br>(-0.96~-0.8)
Zambia	2925.5<br>(2386.7-3588.4)	2525.6<br>(2060.4-3097.9)	3736.6<br>(2828.8-4843.4)	1388.6<br>(1051.3-1800)	-2.4<br>(-2.86~-1.94)
Zimbabwe	3730.7<br>(2911.3-4657.9)	2080.3<br>(1623.4-2597.3)	6233<br>(4922.9-7629.3)	2348<br>(1854.5-2874)	0.75<br>(0.27~1.23)

eTable 7. Changes in prevalence cases of malnutrition in overall population at all ages at according to population-level determinants including aging, population growth and epidemiological change from 1990 to 2019 at the global level and by SDI quintiles stratified by sexes

Abbreviations: SDI: Socio-demographic Index.

Location	Sex	Overll difference	Aging	Population	Epidemiological change	Percent		
						Aging	Population	Epidemiological change
Global	Both	80135795	-70152030	720105605	-569817780	-87.54	898.61	-711.07
Middle SDI	Both	-76740242	-13620266	179511316	-242631293	17.75	-233.92	316.17
Low-middle SDI	Both	45601741	-5856826	329682378	-278223811	-12.84	722.96	-610.12
High-middle SDI	Both	-59159728	-2582546	38432092	-95009273	4.37	-64.96	160.60
Low SDI	Both	175492614	-1611543	334591931	-157487774	-0.92	190.66	-89.74
High SDI	Both	-4867893	879131	16806111	-22553135	-18.06	-345.24	463.30
Global	Female	190479448	-34343484	395801564	-170978631	-18.03	207.79	-89.76
Middle SDI	Female	19692035	-5701734	103923072	-78529304	-28.95	527.74	-398.79
High-middle SDI	Female	-15287429	-1954119	22161129	-35494438	12.78	-144.96	232.18
High SDI	Female	-2115352	-903836	10411544	-11623060	42.73	-492.19	549.46
Low SDI	Female	108915456	186751	168698147	-59969442	0.17	154.89	-55.06
Low-middle SDI	Female	79305072	738973	178653887	-100087787	0.93	225.27	-126.21
Global	Male	-110343653	-35647750	325007747	-399703651	32.31	-294.54	362.24
Middle SDI	Male	-96432277	-8251457	76331918	-164512739	8.56	-79.16	170.60
Low-middle SDI	Male	-33703332	-6904592	151507226	-178305966	20.49	-449.53	529.05
Low SDI	Male	66577158	-1851217	165863973	-97435598	-2.78	249.13	-146.35
High-middle SDI	Male	-43872299	-951863	16108904	-59029340	2.17	-36.72	134.55
High SDI	Male	-2752541	1854191	6117047	-10723778	-67.36	-222.23	389.60

eTable 8. Changes in global incidence cases and incidence over the next 25 years, based on Nordpred

Year	Both		Female		Male	
	Number	Rate	Number	Rate	Number	Rate
2022	20752216	5492.102	10728490	5033.142	10023726	6086.098
2023	20833664	5332.986	10843695	4924.063	9989969	5861.343
2024	20925402	5184.767	10969497	4826.817	9955905	5646.103
2025	21241752	5098.757	11186951	4774.142	10054801	5516.048
2026	21519349	5008.857	11390601	4717.939	10128748	5382.07
2027	21731500	4910.13	11566812	4652.591	10164688	5240.208
2028	21876734	4804.41	11713276	4579.16	10163459	5093.148
2029	21979795	4694.837	11841082	4500.98	10138713	4943.503
2030	22273298	4625.107	12045293	4449.44	10228006	4850.64
2031	22567375	4549.049	12251957	4391.846	10315418	4751.034
2032	22874558	4465.656	12467709	4327.547	10406850	4643.182
2033	23223991	4368.274	12707566	4250.122	10516425	4520.113
2034	23612771	4259.909	12969922	4161.842	10642849	4385.852
2035	24244083	4192.907	13339983	4104.914	10904101	4305.826
2036	24865583	4133.796	13705554	4055.123	11160029	4234.692
2037	25448228	4088.515	14051061	4018.321	11397167	4178.503
2038	26009968	4059.778	14384299	3997.499	11625670	4139.573
2039	26573989	4041.705	14717322	3987.224	11856667	4111.437
2040	27128835	4028.382	15043768	3981.169	12085067	4088.741
2041	27648873	4012.754	15353288	3972.877	12295586	4063.687
2042	28117030	3989.78	15638028	3957.219	12479002	4031.349
2043	28538877	3958.596	15900126	3932.819	12638751	3991.509
2044	28931902	3922.503	16147554	3902.943	12784348	3947.491
2045	29297956	3883.092	16381574	3869.687	12916382	3900.228
2046	29638914	3841.662	16603424	3834.802	13035490	3850.435

eTable 9. The population of individuals over 70 and overall population in 1990 and 2021

Location	Sex	Age	Year	Number
Global	Male	All ages	1990	2685743010
Global	Female	All ages	1990	2647880415
Global	Both	All ages	1990	5333623426
Global	Male	70+ years	1990	82718035.76
Global	Female	70+ years	1990	119288906.9
Global	Both	70+ years	1990	202006942.6
Global	Male	All ages	2021	3959391439
Global	Female	All ages	2021	3931961862
Global	Both	All ages	2021	7891353301
Global	Male	70+ years	2021	217423198.1
Global	Female	70+ years	2021	276944957.1
Global	Both	70+ years	2021	494368155.2

eTable 10. The prevalence cases of malnutrition among individuals over 70 and overall population in 1990 and 2021

Abbreviations: SDI: Socio-demographic Index.

Location	Sex	Age	Year	
			1990	2021
Middle SDI	Male	All ages	261643340.5	165211063.5
Middle SDI	Female	All ages	273933765.3	293625800.3
Middle SDI	Both	All ages	535577105.9	458836863.7
Middle SDI	Male	70+ years	5790068.325	13635447.12
Middle SDI	Female	70+ years	6334795.19	17046813.99
Middle SDI	Both	70+ years	12124863.52	30682261.12
Low-middle SDI	Male	All ages	309850505.3	276147173.4
Low-middle SDI	Female	All ages	302298073.2	381603145.6
Low-middle SDI	Both	All ages	612148578.4	657750319.1
Low-middle SDI	Male	70+ years	6818784.093	13329837.09
Low-middle SDI	Female	70+ years	5993197.636	15027588.75
Low-middle SDI	Both	70+ years	12811981.73	28357425.84
Low SDI	Male	All ages	167196312.6	233773470.6
Low SDI	Female	All ages	154262339.2	263177795.1
Low SDI	Both	All ages	321458651.8	496951265.7
Low SDI	Male	70+ years	3033940.677	5051371.212
Low SDI	Female	70+ years	2466000.962	5149303.74
Low SDI	Both	70+ years	5499941.64	10200674.95
High SDI	Male	All ages	27749646.94	24997106.16
High SDI	Female	All ages	51346582.48	49231230.64
High SDI	Both	All ages	79096229.42	74228336.79
High SDI	Male	70+ years	2457836.232	5887300.333
High SDI	Female	70+ years	3308373.444	6800404.974
High SDI	Both	70+ years	5766209.675	12687705.31
Global	Male	All ages	863913112.8	753569459.4
Global	Female	All ages	901197650.6	1091677099
Global	Both	All ages	1765110763	1845246558
Global	Male	70+ years	21612270.98	44275156.43
Global	Female	70+ years	22750113.1	53321103.86
Global	Both	70+ years	44362384.08	97596260.29
High-middle SDI	Male	All ages	96793886.54	52921587.69
High-middle SDI	Female	All ages	118508705	103221276.2
High-middle SDI	Both	All ages	215302591.6	156142863.9
High-middle SDI	Male	70+ years	3493266.514	6340086.124

High-middle SDI	Female	70+ years	4620532.354	9254777.939
High-middle SDI	Both	70+ years	8113798.868	15594864.06

eFigure 1. The study flowchart of worldwide burden of nutritional deficiencies in individuals aged 70 and older from 1990 to 2021.

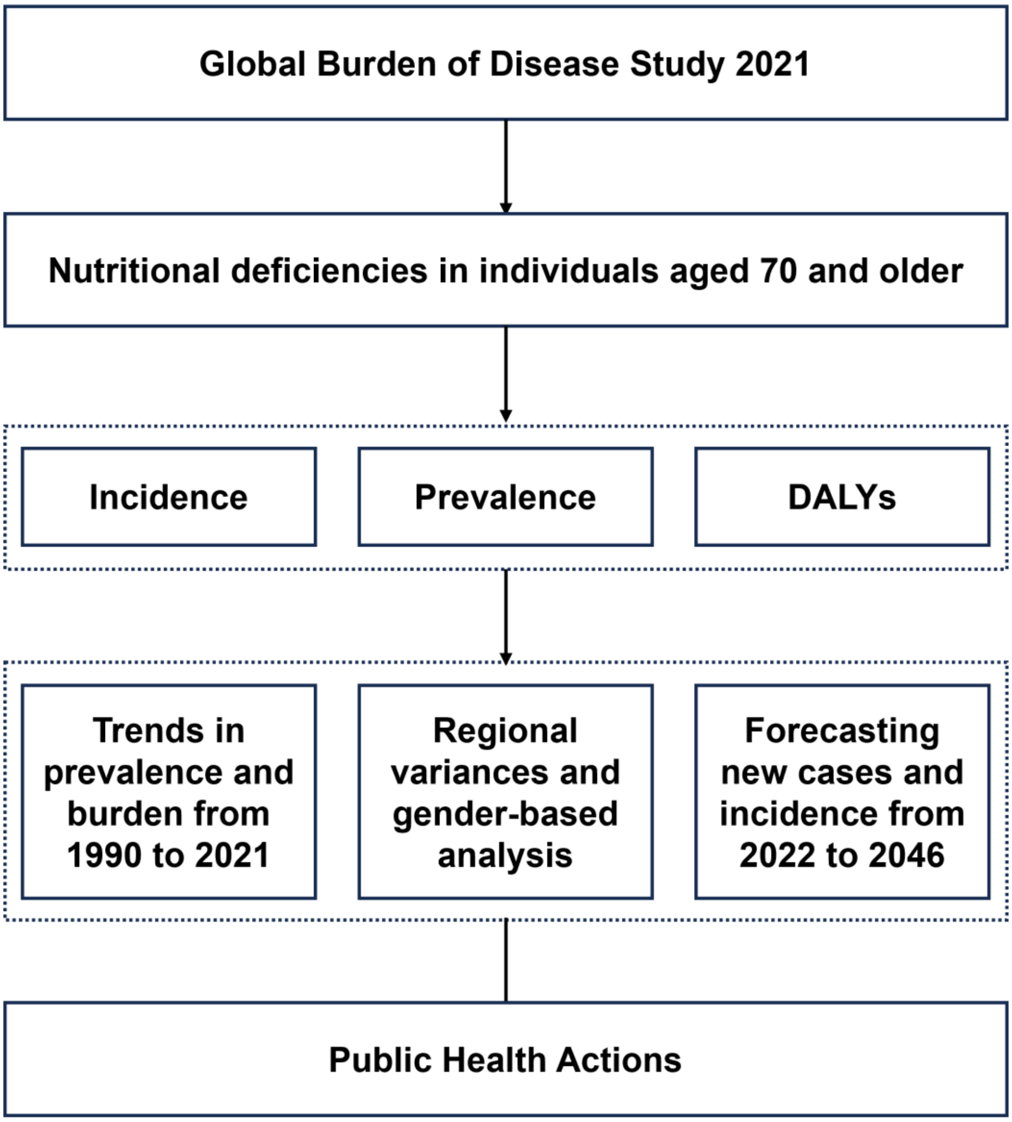

Abbreviations: DALYs: disability-adjusted life years.

eFigure 2. Joinpoint regression analysis of prevalence for nutritional deficiency in the elderly from 1990 to 2021. **(A)** Global; **(B)** high SDI; **(C)** high-middle SDI; **(D)** middle SDI; **(E)** low-middle SDI; **(F)** low SDI.

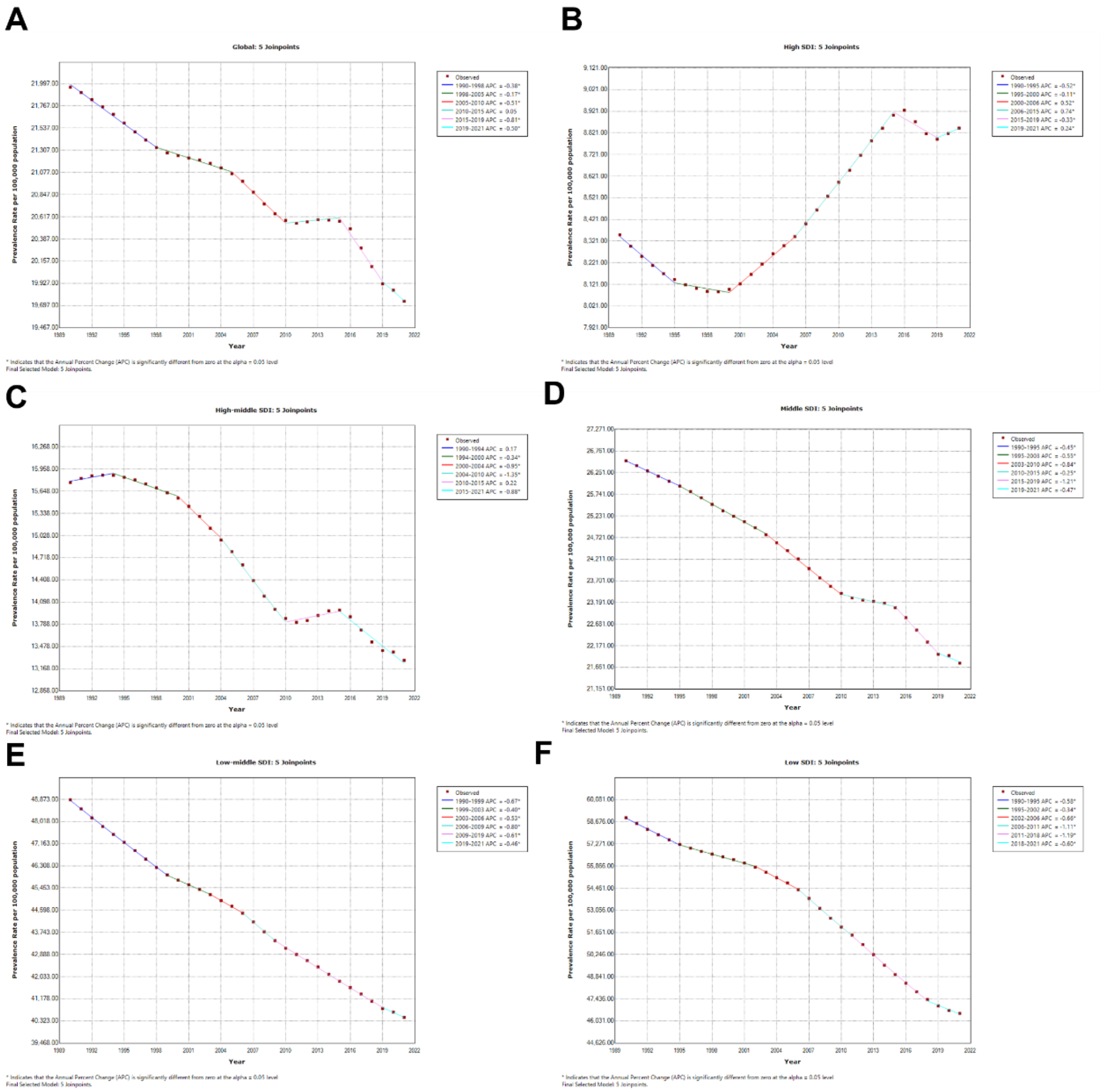

Abbreviations: SDI: Socio-demographic Index.

eFigure 3. Prevalence **(A)** and DALYs rate **(B)** of malnutrition among individuals over 70 by year and sex.

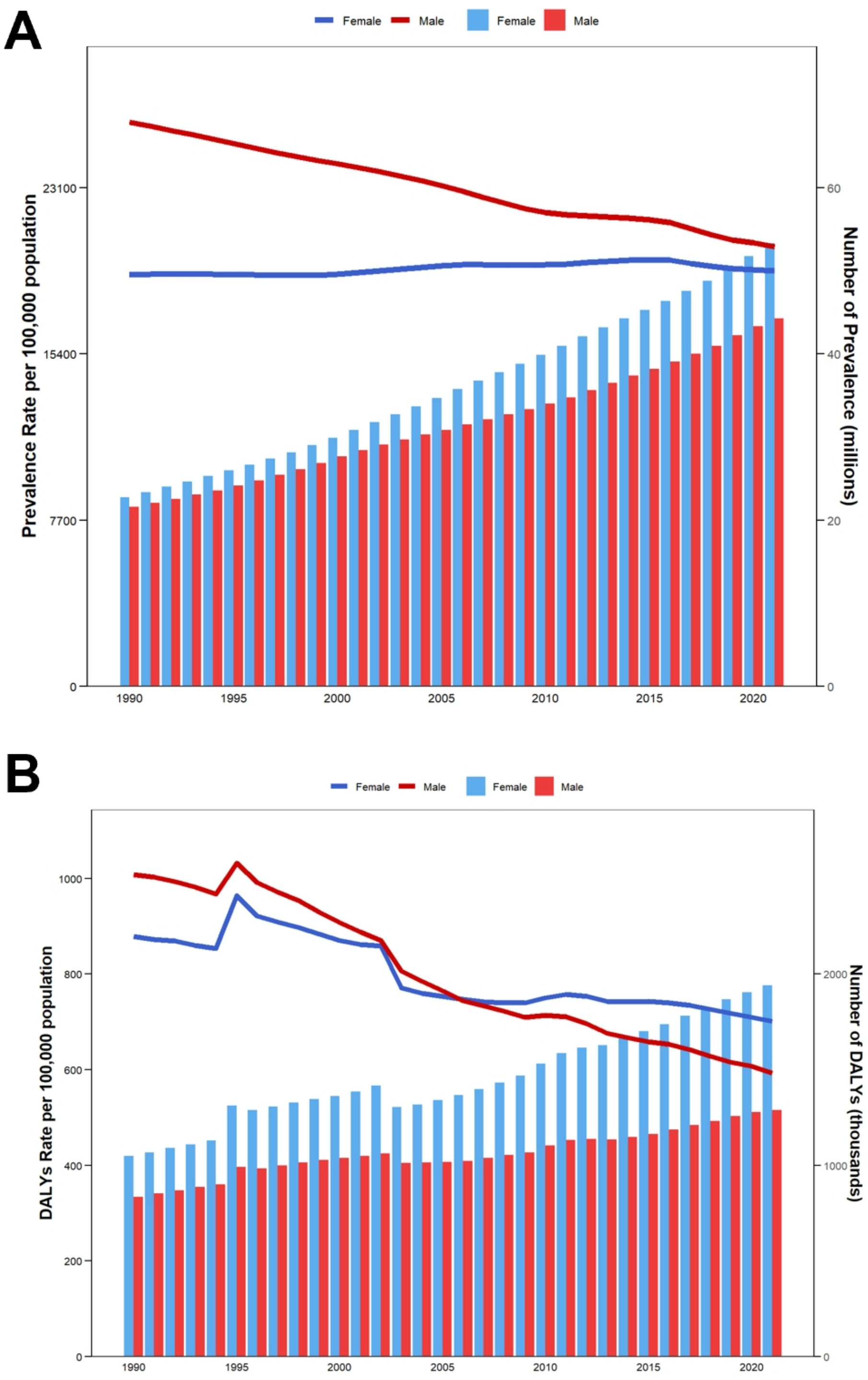

Abbreviations: DALYs: disability-adjusted life years.

eFigure 4. Prevalence **(A)** and DALYs rate **(B)** of malnutrition among individuals over 70 by year and regions with 95% uncertainty intervals.

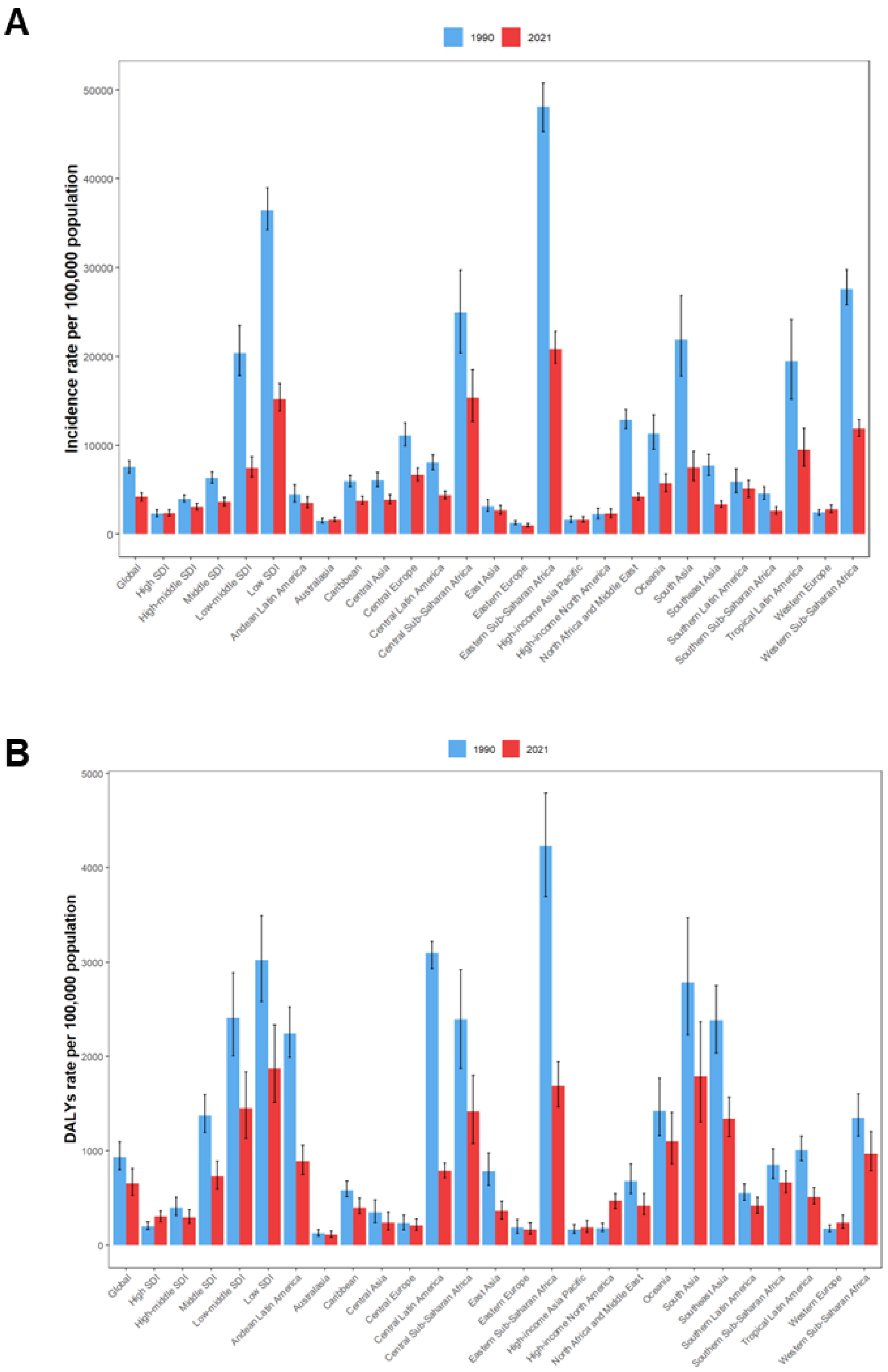

Abbreviations: SDI: Socio-demographic Index; DALYs: disability-adjusted life years.

eFigure 5. (A) The incidence of malnutrition among individuals over 70 in 2021. **(B)** The relative change in case number of incidence of malnutrition among individuals over 70 from 1990 to 2021. **(C)** The EAPC of incidence of malnutrition among individuals over 70 from 1990 to 2021.

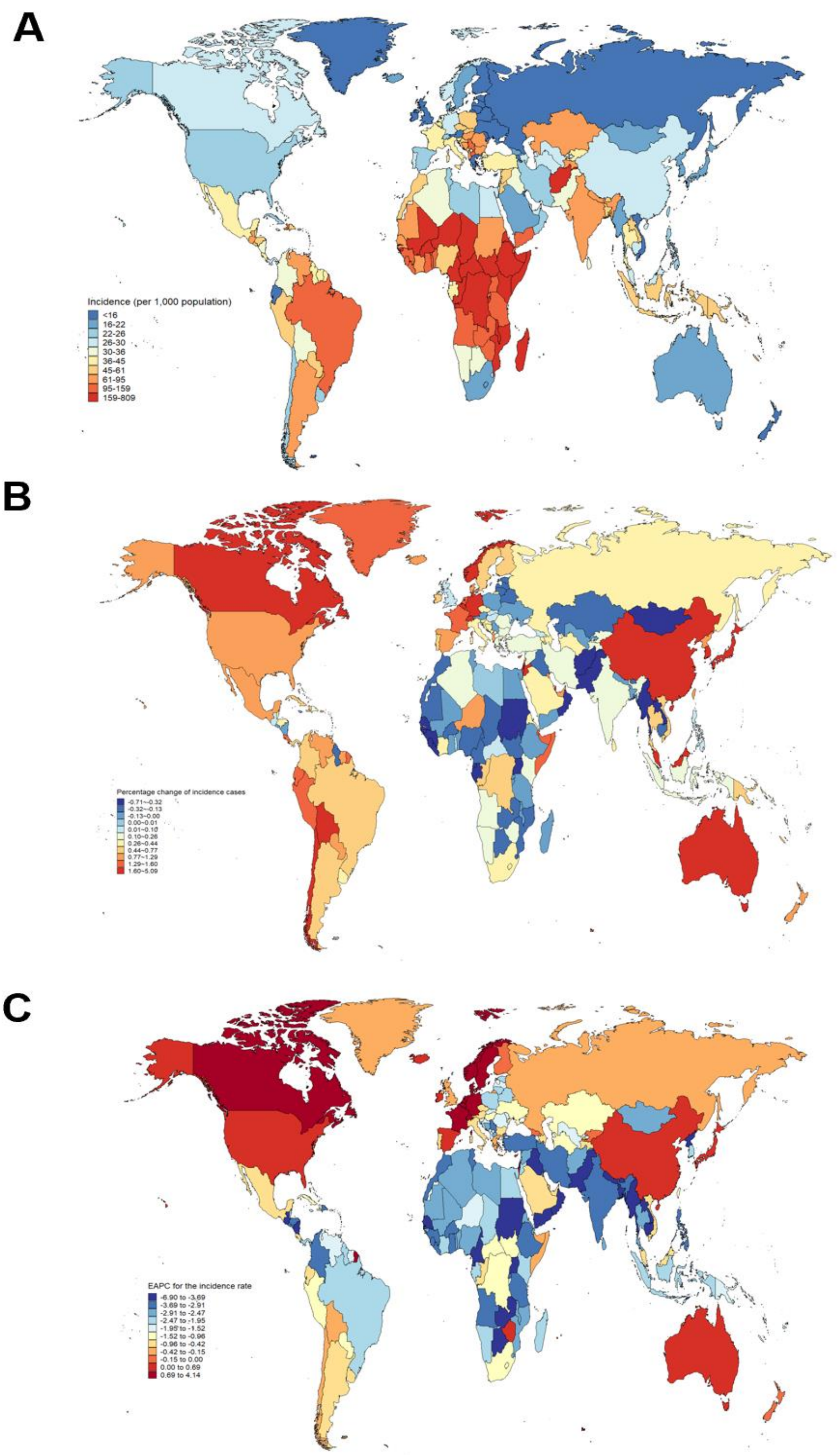

Abbreviations: EAPC, estimated annual percentage change.

eFigure 6. (A) The DALYs rate of malnutrition among individuals over 70 in 2021. **(B)** The relative change in case number of DALYs of malnutrition among individuals over 70 from 1990 to 2021. **(C)** The EAPC of DALYs rate of malnutrition among individuals over 70 from 1990 to 2021.

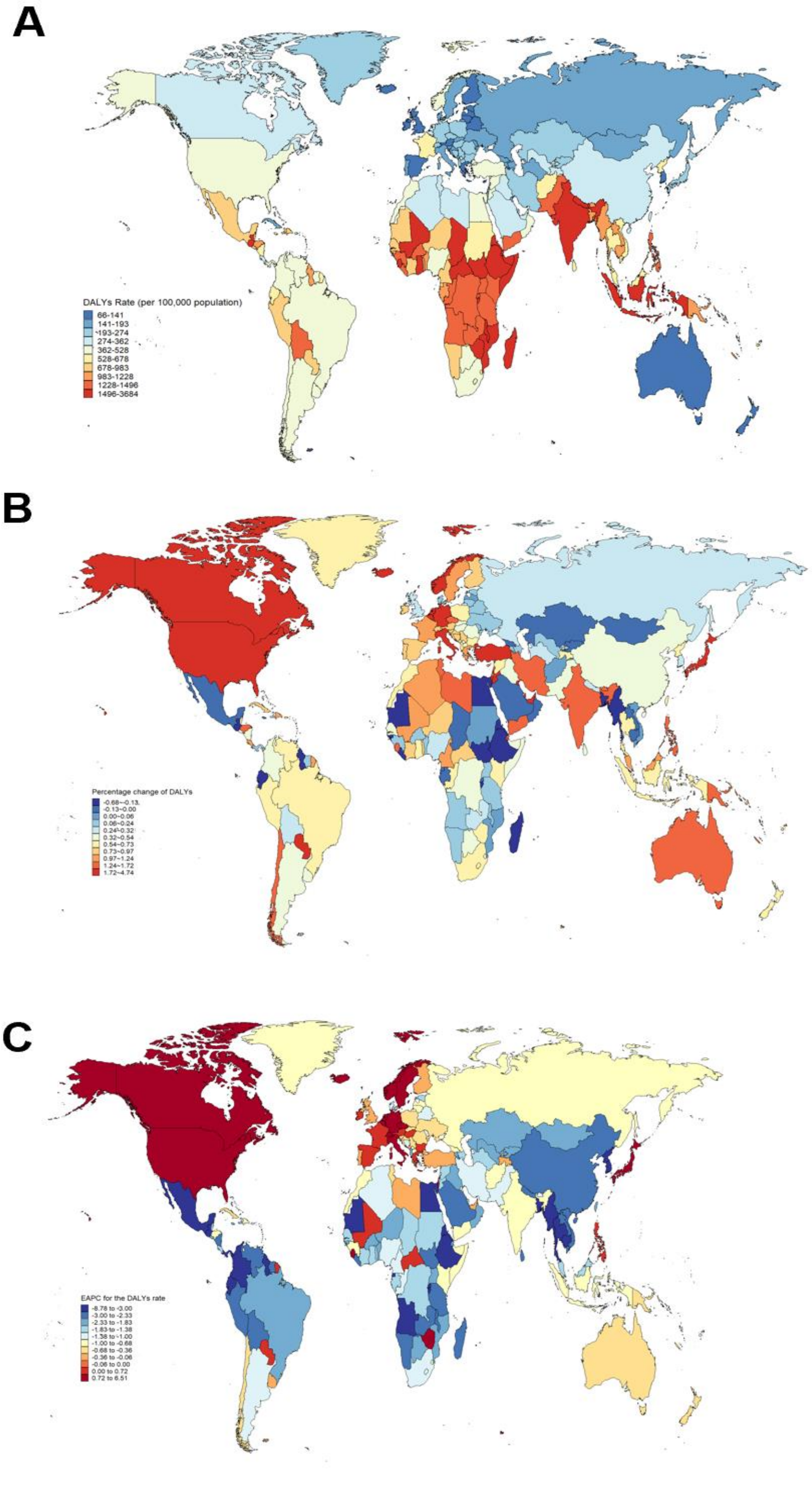

Abbreviations: EAPC, estimated annual percentage change; DALYs: disability-adjusted life years.

eFigure 7. (A) DALYs rates for malnutrition among individuals over 70 for 21 GBD regions by Socio-demographic Index, 1990–2021.Expected values based on Socio-demographic Index and disease rates in all locations are shown as the black line. The correlation between EAPC and **(B)** malnutrition incidence among individuals over 70 in 1990 and **(C)** HDI in 2022. The circles represent countries that were available on HDI data. The size of circle is increased with the cases of malnutrition. The ρ indices and p values presented in **(B)** and **(C)** were derived from Pearson correlation analysis.

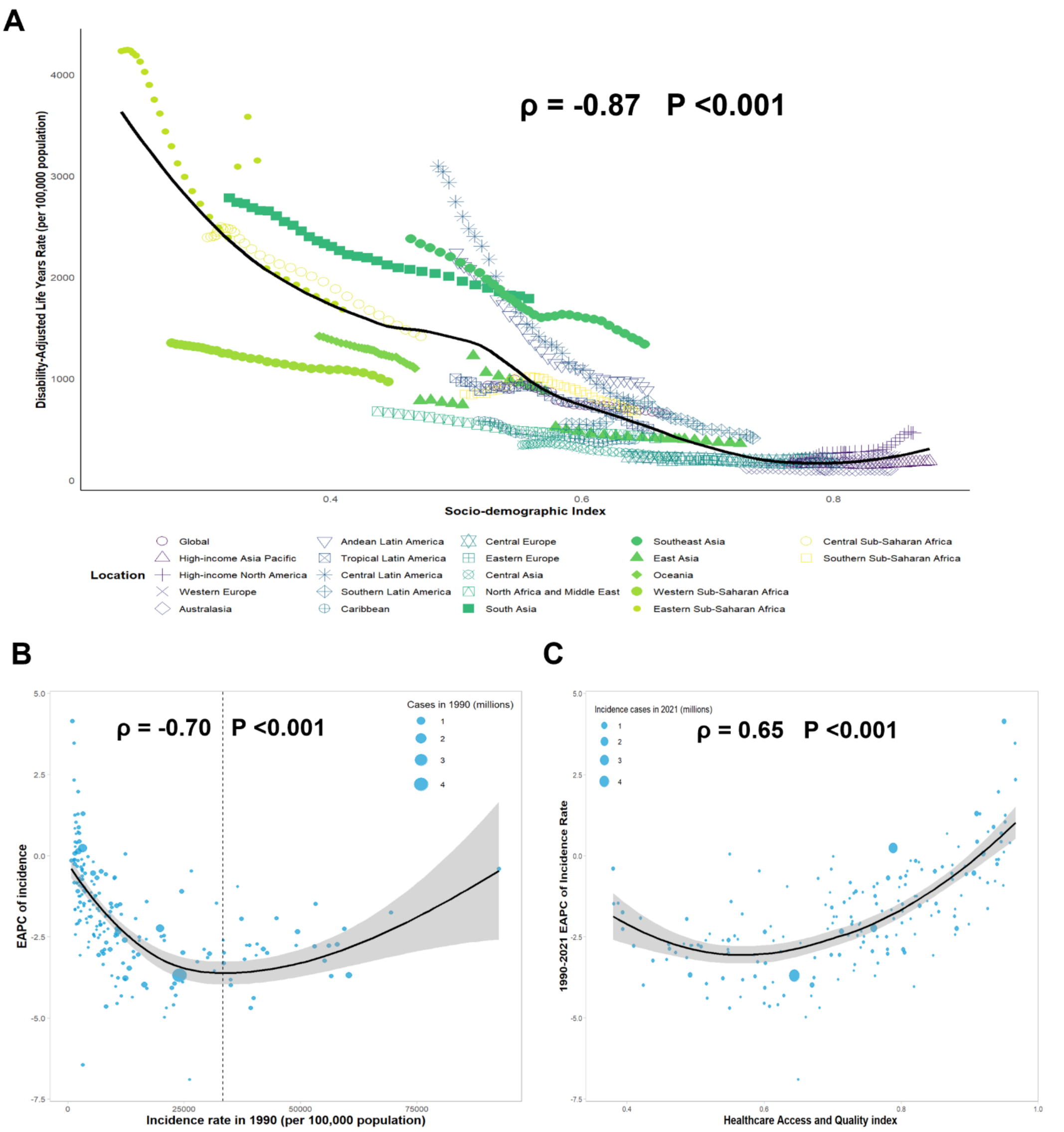

Abbreviations: EAPC, estimated annual percentage change; HDI, human development index; DALYs: disability-adjusted life years. GBD: the Global Burden of Diseases.

eFigure 8. DALYs rates for malnutrition among individuals over 70 for 204 countries and territories by Socio-demographic Index, 1990–2021.Expected values based on Socio-demographic Index and disease rates in all locations are shown as the black line.

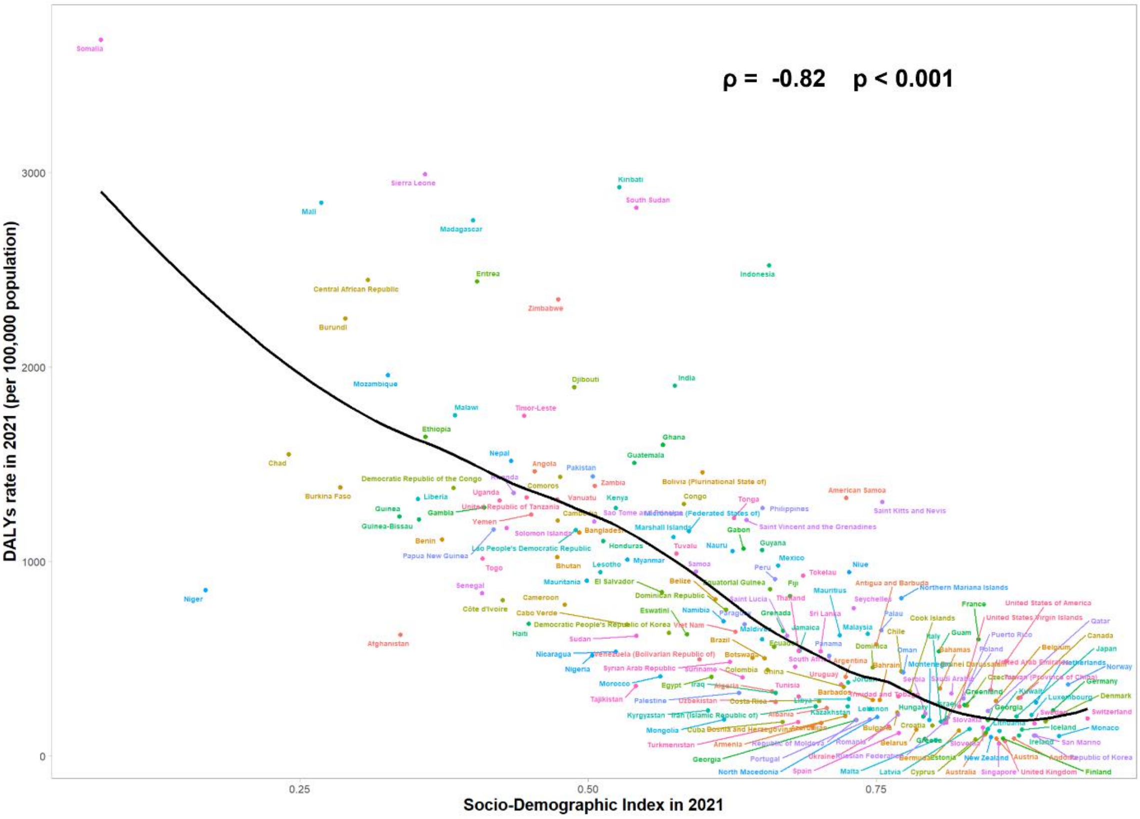

The ρ indices and p values presented in the figure was derived from Pearson correlation analysis.

Abbreviations: DALYs: disability-adjusted life years.
